# Emergence of terpene cyclization in *Artemisia annua*

**DOI:** 10.1038/ncomms7143

**Published:** 2015-02-03

**Authors:** Melissa Salmon, Caroline Laurendon, Maria Vardakou, Jitender Cheema, Marianne Defernez, Sol Green, Juan A. Faraldos, Paul E. O’Maille

**Affiliations:** 1 John Innes Centre, Department of Metabolic Biology, Norwich Research Park, Norwich NR4 7UH, UK; 2 John Innes Centre, Computational and Systems Biology, Norwich Research Park, Norwich NR4 7UH, UK; 3 Institute of Food Research, Analytical Sciences Unit, Norwich Research Park, Norwich NR4 7UA, UK; 4 Plant and Food Research, 120 Mt Albert Road, Sandringham, Auckland 1025, New Zealand; 5 School of Chemistry, Cardiff University, Main Building, Park Place, Cardiff CF10 3AT, UK; 6 Institute of Food Research, Food & Health Programme, Norwich Research Park, Norwich NR4 7UA, UK

## Abstract

The emergence of terpene cyclization was critical to the evolutionary expansion of chemical diversity yet remains unexplored. Here we report the first discovery of an epistatic network of residues that controls the onset of terpene cyclization in *Artemisia annua*. We begin with amorpha-4,11-diene synthase (ADS) and (*E*)-β-farnesene synthase (BFS), a pair of terpene synthases that produce cyclic or linear terpenes, respectively. A library of ~27,000 enzymes is generated by breeding combinations of natural amino-acid substitutions from the cyclic into the linear producer. We discover one dominant mutation is sufficient to activate cyclization, and together with two additional residues comprise a network of strongly epistatic interactions that activate, suppress or reactivate cyclization. Remarkably, this epistatic network of equivalent residues also controls cyclization in a BFS homologue from *Citrus junos.* Fitness landscape analysis of mutational trajectories provides quantitative insights into a major epoch in specialized metabolism.

## Abstract (web summary)

Terpene cyclases are ring-forming enzymes found in many biosynthetic pathways, but the evolutionary origins of the cyclization mechanism is not well understood. Here, the authors use structure-guided breeding to identify an epistatic network that controls the onset of cyclization activity in *Artemisia annua*.

## Graphical abstract

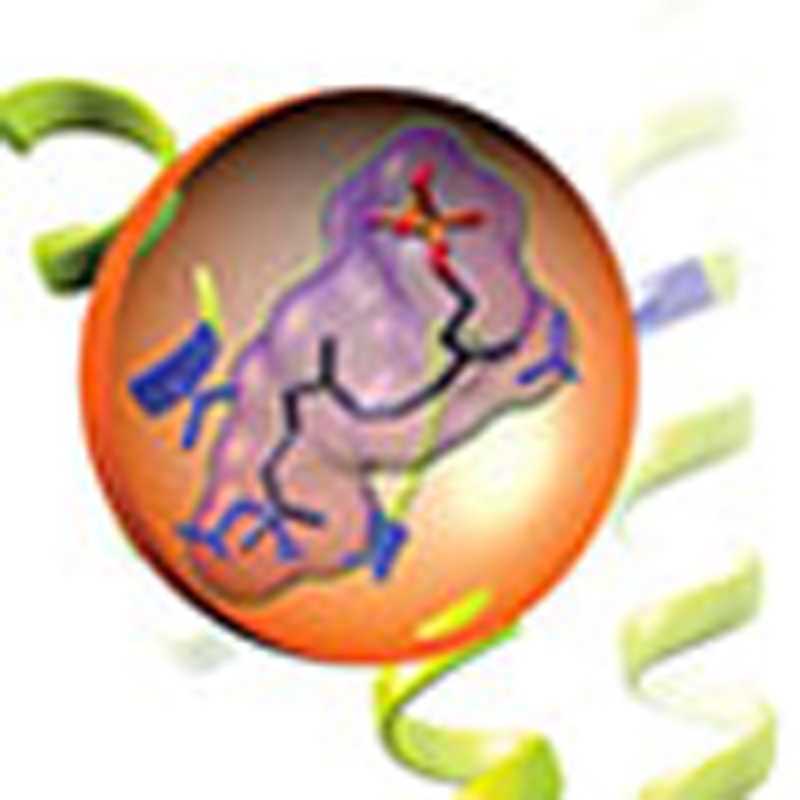

The emergence of novel catalytic function underlies the evolutionary expansion of metabolism with profound biological implications^1^. Ring-forming reactions (cyclization) catalysed by terpene synthases (TPSs) were central to this expansion, giving rise to cyclic terpenes, the most diverse family of specialized (secondary) metabolites. Cyclic terpenes mediate essential interactions between organisms, enabling plants to attract pollinators^2^ and natural enemies of herbivores^3^, repel microbial pathogens^4^, and to conduct symbiotic relations^5^. They also provide a rich source of bioactive compounds for human uses ranging from flavours and fragrances to medicinal compounds such as artemisinin, a naturally occurring terpenoid extracted from *Artemisia annua*, the most effective treatment for malaria^6^. However, despite the intensive study of the TPS enzyme family, little is known about the evolutionary emergence of the cyclization mechanism.

Cyclization is the major gateway to chemical diversity in isoprenoid specialized metabolism; TPSs convert a few universal substrates into hundreds of often stereochemically complex mono- and polycyclic hydrocarbons^7^, which seed the biosynthesis of thousands of derivatives through downstream metabolic pathways. The conserved class I terpenoid synthase fold, as revealed from the first crystal structures^8^,^9^, has evolved the unique catalytic function of cyclization, converting C-10, C-15 and C-20 isoprenyl diphosphate substrates into mono-, sesqui- and diterpenes, respectively. TPS-catalysed reactions can achieve extraordinary mechanistic complexity, often involving numerous electrophilic cyclizations and rearrangements according to well-established chemical rules^10^,^11^, stemming from intrinsic and induced chemical reactivity^12^. In *A. annua*, amorpha-4,11-diene synthase (ADS) catalyses a multi-step electrophilic reaction that converts the C-15 sesquiterpene substrate farnesyl pyrophosphate (FPP) into amorpha-4,11-diene, the bicyclic hydrocarbon precursor of artemisinin. By contrast, (*E)*-β-farnesene synthase (BFS) catalyses one of the simplest TPS reactions where the linear carbocation is quenched before cyclization can happen; formally, this involves release of pyrophosphate followed by proton elimination from either a transoid or cisoid farnesyl carbocation to produce the linear hydrocarbon (*E*)-β-farnesene, an aphid alarm pheromone^13^. Isotope labelling studies suggest that isomerization via a tertiary diphosphate intermediate (nerolidyl diphosphate) may be a more general feature of all 1,4-conjugate elimination reactions catalysed by terpene synthases like BFS^14^,^15^.

From an evolutionary perspective, the emergence of the cyclization mechanism was a critical and defining step of the TPS family, essential for the biogenesis of structurally complex metabolites. While the activities of the earliest TPSs are unknown, the modern TPSs (both linear and cyclic terpene producers) likely arose by numerous distinct paths. According to one possible scenario, a ‘minimalist’ class of enzymes like BFS preceded the more ‘complex’ functions of ADS, thereby involving a gain of cyclization function mutation(s) to unlock ring-forming catalytic activities. To a first approximation, natural variation at positions within the active site and surrounding regions of the protein structure would most strongly influence the onset of cyclization. Thus, identification of key substitutions that unlock cyclization may provide fundamental structural insights and mechanistic clues about how ring formation evolved and hence a basis to explore this phenomenon in the greater TPS family from plants to microbes.

To investigate the emergence of terpene cyclization, we initially focus on the TPS enzymes of *A. annua*. We use structure-based combinatorial protein engineering (SCOPE)^16^ to breed natural variation from ADS into the background of BFS, two related enzymes that share 49% amino-acid identity. Through successive rounds of screening, we identify a dominant mutation that activates cyclization in BFS, and uncover additional mutations that form an extended epistatic network that is able to activate, suppress or reactivate cyclization. We further demonstrate this residue network is able to activate cyclization in a BFS homologue from *Citrus junos*. Finally, we calculate the fitness landscapes of *A. annua* and *C. junos* to directly measure how the protein background shapes the functional roles and pattern of epistatic interactions among residues in the network. These results provide unique insights into the emergence of cyclization across distinct plant lineages that underlie the evolutionary expansion of specialized metabolism.

## Results

### Breeding natural mutations from ADS into BFS

**Figure 1:**
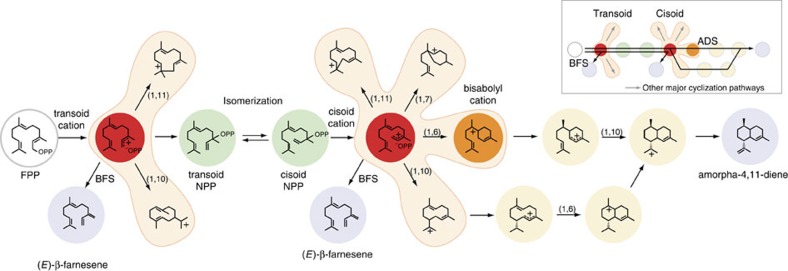
Catalytic mechanisms of TPS enzymes. Terpene synthases are carbon-oxygen lyases as illustrated by the core sesquiterpene synthase mechanism (overview inset). TPSs catalyse the metal-dependent cleavage (ionization) of the carbon-oxygen bond of isoprenoid pyrophosphate substrates, such as the 15-carbon farnesyl pyrophosphate (FPP) leading to numerous potential outcomes. In the BFS-catalysed mechanism, deprotonation of the either the transoid or cisoid farnesyl cation can yield (*E*)-β-farnesene, although BFS from *Mentha x piperita* has been demonstrated to involve isomerization via the tertiary diphosphate intermediate nerolidyl diphosphate (NPP)^14^. Of note, additional linear terpene alcohol products may also form from quenching either the transoid or cisoid cations (not shown). Mostly, all TPSs (referred to as cyclases) promote the intramolecular cyclization of carbocations, often followed by further electrophilic rearrangements including hydride shifts, alkyl shifts, and/or ring closures before quenching (as shown for ADS). Numbers in parentheses above arrows indicate cyclization steps. Cyclization commences from either the transoid or cisoid farnesyl cations (red), bridged by an isomerization step of the intermediate nerolidyl pyrophosphate (green spheres). The ADS reaction mechanism illustrates transit through isomerization, where either 1,6 or 1,10 cyclization pathways (light yellow spheres) lead to energetically viable rearrangement pathways^49^ before converging on the amorpha-4,11-diene final product.

**Figure 2:**
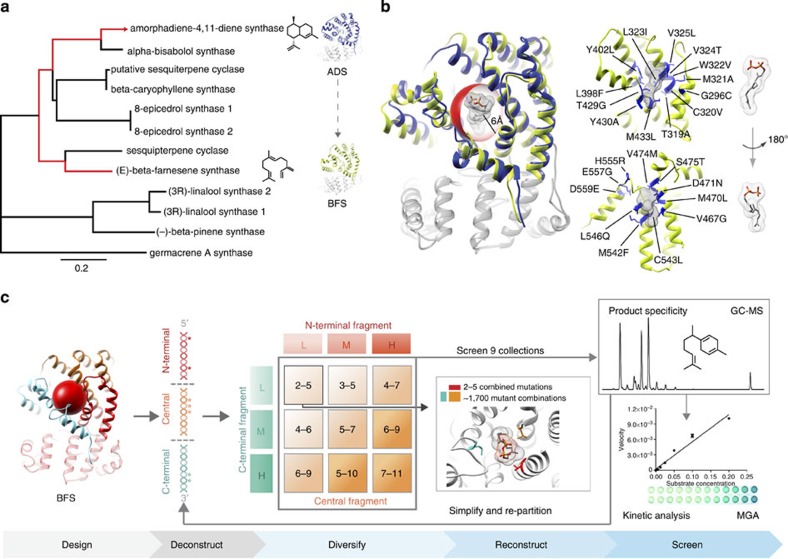
Design and synthesis of BFS gene library. (**a**) A phylogenetic tree was constructed using full-length mono- and sesquiterpene synthase protein sequences from *Artemisia annua*, where the bar at the bottom of the tree corresponds to sequence relatedness. (**b**) Superimposed structural models of BFS (yellow) and ADS (blue) illustrate the conserved terpenoid synthase fold. Identification of variable residues between BFS and ADS within a 6 Å sphere of the active site centre (red sphere) was based on structural analysis. A cross-section of the active site reveals 24 variable residue positions targeted for mutation in breeding experiments (highlighted blue). Numbering is relative to the *A. annua* BFS sequence. (**c**) A schematic of the experimental strategy is illustrated. Variable residues from structure-sequence analysis (design) were encoded into gene fragments of BFS (deconstruct). Combinatorial assembly of fragments in nine discrete pools (grid) was accomplished via structure-based combinatorial engineering (SCOPE) to assort mutation at low (L), medium (M) or high (H) intensities into full-length products (reconstruct) (Supplementary Table 4b–e)). Individual pools were screened for active mutants using gas chromatography—mass spectrometry (GC–MS) and kinetic characterization was performed using the malachite green assay (MGA).

The *A. annua* TPSs provide an ideal starting point to experimentally examine the critical structural features underlying the emergence of cyclization, given the contrasting mechanisms of BFS and ADS (Fig. 1). To identify candidate amino-acid substitutions to incorporate into BFS, we mapped the variable sequence positions between ADS and BFS onto structural models. Through sequence-structure analysis, we localized 24 substitutions within a 6-angstrom radius of the active site centre, which included 5 second-tier positions and 3 positions in a flexible loop that caps the active site (Fig. 2a,b). A complete library encoding this combinatorial complexity would total 2^24^ mutants (that is, 16,777,216). We anticipated that the active site would potentially require significant remodelling to accommodate cyclization; therefore, we designed our library to sample multiple mutational combinations simultaneously in the active site. Given technical limitations to screening throughput (discussed below), we designed oligonucleotides to encode a subset of combinations yielding 27,524 theoretically possible mutations (Supplementary Figs 1 and 2; Supplementary Tables 1 and 2). We used structure-based combinatorial protein engineering (SCOPE)^16^,^17^ to breed this diversity into BFS and create a gene library as nine discrete collections (~3,000 distinct variants each). Each collection contained varying numbers of mutations, ranging from 2–5 to 7–11 positions mutated simultaneously. We conducted three rounds of screening, sampling individual mutants from each collection (totalling 754 mutants). By synthesizing the library as unique subsets, we significantly enhanced screening probabilities^16^, which also gave us flexibility to shift sampling intensity among different collections and further partition our library into subpopulations in response to screening results (described below).

### Discovery of a dominant mutation that activates cyclization

To identify cyclization activities in our libraries, we used gas chromatography-mass spectrometry (GC–MS). While GC–MS is a low-throughput analytical technique, which imposed limitations on the numbers of mutants we were able to characterize in our screening efforts (~20 min per run), it enables resolution of hydrocarbon products and unambiguous identification of cyclic terpenes. To biochemically characterize mutant enzymes, we sampled ~32 mutants from each of the nine mutant pools (282 mutants), expressed recombinant proteins in *E. coli* and assayed purified enzymes by GC–MS (Fig. 2c and Supplementary Fig. 3). Finally, we sequenced selected clones, verified activities and measured the kinetic properties of the encoded proteins^18^ (Supplementary Table 3).

**Figure 3:**
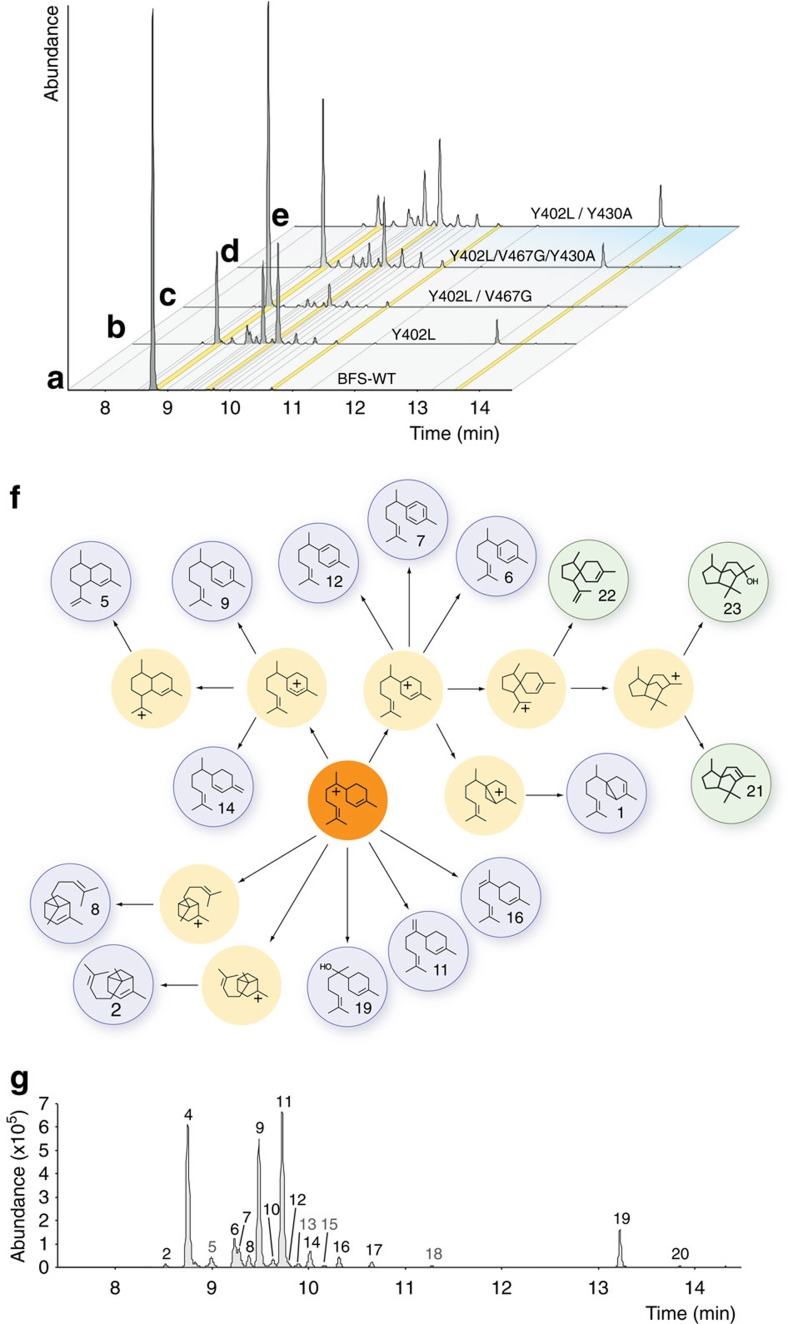
Cyclic terpene product diversity synthesized by mutants of the BFS 6 Å library. Total ion chromatograms of (**a**) the wild-type (WT); (**b**) the dominant mutant Y402L, (**c**) the second-site revertant mutant Y402L/V467G, (**d**) the re-activated cyclic mutant Y402L/V467G/Y430A and (**e**) the super cyclase Y402L/Y430A are shown; linear products are indicated with a yellow line, all other products are cyclic. (**f**) A reaction scheme illustrates the cyclization pathways leading to product diversity observed in BFS mutants (blue circles) from rearrangements (light yellow circles) via the bisabolyl cation (orange circle). Mechanisms for linear product formation (10, α-farnesene; 17, nerolidol; and 20, farnesol) are not shown. (**g**) An annotated reference total ion chromatogram (Y402L). Labelled peaks and chemical structures were identified either by MS comparisons with authentic standards or matched to reference mass spectral databases (Supplementary Fig. 5 and Supplementary Table 5). Products of BFS mutants (blue circles, and also labelled in the total ion chromatogram) are 1, sesquithujene; 2, α-exo-bergamotene; 3, Peak 1; 4, (*E*)-β-farnesene; 5, putative amorphadiene isomer; 6, γ-curcumene; 7, ar-curcumene; 8, cis-α-bergamotene; 9, zingiberene; 10, α-farnesene; 11, β-bisabolene; 12, β-curcumene; 13, Peak 2; 14, β-sesquiphelladrene; 15, Peak 3; 16, α-bisabolene; 17, nerolidol; 18, Peak 4; 19, α-bisabolol; 20, farnesol. Mechanistically related products from *A. annua* (green circles) are 21, α-cedrene; 22, acoradiene; 23, 8-epi-cedrol.

From our initial screen of 282 BFS mutants, most clones produced soluble protein. However, 94% of mutants were inactive, while 5% retained BFS WT-like farnesene synthase activity (Supplementary Fig. 4). Significantly, we identified three mutants (1%) that produced multiple cyclic terpene products (Supplementary Table 4a). Following sequence analysis, further mutagenesis and screening we identified Y402L as a single common substitution among cyclic terpene-producing enzymes. Incorporating the single Y402L substitution into wild-type BFS was sufficient to induce the production of 15 distinct cyclic terpenes comprising ~75% of enzyme-catalysed products, including 1.3% of a putative amorphane sesquiterpene (Fig. 3b,f). We further characterized Y402L by steady-state kinetic measurements, revealing that catalytic efficiency was only slightly reduced (*k*_cat_/*K*_M_=10.5 × 10^−3^ versus 5.9 × 10^−3^ μM^−1^ s^−1^ for BFS WT and Y402L, respectively) (Table 1). Consequently, removing the Y402L mutation abolished cyclization and the three cyclic terpene-producing mutants reverted to enzymes with BFS WT-like product specificity. This result demonstrates that the single Y402L is a dominant natural mutation that provides a viable gateway to cyclization in *A. annua* BFS.

Our discovery of the Y402L mutation prompted us to screen for more functionally diverse cyclases in the Y402L background and to search for an alternative position(s) that may activate cyclization in the wild-type (Y402) background. We also identified a deleterious mutation G296C prevalent among inactive mutants in our initial screen. Therefore, we removed G296C and split our libraries into subsets containing either the Y402L mutation or the wild-type (Y402) background and conducted a second round of screening (Supplementary Table 4a). Throughout our screening, we shifted sampling intensity to library subsets that yielded the highest number of active clones. In total, we sampled 142 mutants from the wild-type background (Y402) group, where all 25 active mutants (18%) maintained wild-type BFS product specificity. This result suggests that cyclization was Y402L-dependent among the diversity that we screened. In the Y402L group (321 mutants), we found a similar percentage (15%) of active mutants (47) with 37 unique mutants that produced multiple (8 to 15) cyclic products. While the vast majority of cyclases were promiscuous, (Supplementary Figs 5 and 6; and Supplementary Tables 5 and 6a), we discovered three mutants with moderate product specificity (~50% single product)—an α-bisabolol synthase (BOS), a β-bisabolene synthase and a cis-α-bergamotene synthase (Supplementary Fig. 7).

To further test the dominance of position 402, we added or removed Y402L from a set of 18 mutants identified in our second screen (Supplementary Table 6). Y402L consistently activated cyclization (by increasing cyclic products by 15-fold from an average of 4 to 58%) across different BFS mutants, each mutant enzyme producing a wide distribution of cyclic products. Conversely, removing Y402L reverted all cyclases to non-cyclizing variants of BFS. Enzyme turnover (*k*_cat_ apparent) was also minimally affected by the addition of Y402L (with an average reduction of 1.5%), which indicates that this mutation is well tolerated in the context of other mutations and primarily affects product specificity (Supplementary Table 8). Taken together, these results suggest a potential role for Y402L as a dominant mutation in the functional divergence of TPSs in *A. annua*.

### An epistatic network controls cyclization

Though Y402L was essential to activate cyclization, we discovered 10 WT-like BFS enzymes in the Y402L background from our second screen, indicating the presence of substitutions that suppress cyclization. Among these, we identified a single common substitution (V467G) that was sufficient to revert cyclase mutants to farnesene-producing synthases (Fig. 3c). The V467G mutation also had deleterious effects on catalytic efficiency (~10-fold reduction in the Y402L/V467G double mutant; Table 1). To evaluate the strength of suppression, we incorporated the V467G mutation into a collection of 10 cyclases (Y402L background) from our second screen. All mutants showed a 4.5-fold decrease in cyclization from an average of 59 to 13% cyclic products. Consequently, removing the V467G mutation from a set of five linear terpene-producing mutants in the Y402L background resulted in a sixfold increase in cyclization from an average of 9 to 55% cyclic products (Supplementary Table 9). Therefore, the V467G mutation displays an epistatic (masking) effect on the Y402L cyclization phenotype according to the classical definition introduced by Bateson^19^.

To identify additional layers of epistatic interactions, we examined our data set and found a natural substitution (Y430A), which overcame second-site suppression by V467G. While the single Y430A substitution alone was unable to activate cyclization in the parent BFS, incorporation of Y430A into the suppressed background (Y402L/V467G) restored cyclization to a total of 55% without impacting catalytic efficiency (*k*_cat_/*K*_M_=0.9 × 10^−3^ μM^−1^ s^−1^) relative to the Y402L/V467G double mutant (Fig. 3d and Table 1). Introduction of the Y430A substitution into seven different Y402L/V467G mutant backgrounds repeatedly stimulated cyclization fourfold from an average of 9 to 37% cyclic products (Supplementary Table 10). Interestingly, pairing Y430A with Y402L both stimulated production of cyclic products to 87% and enhanced catalytic efficiency relative to the single Y402L mutation (*k*_cat_/*K*_M_=7.1 × 10^−3^ μM^−1^ s^−1^) (Fig. 3e and Table 1). This result suggests that the Y402L/Y430A pair of substitutions may have been early critical steps in the emergence of robust cyclase activity in *A. annua*. While previous investigations have shown that epistatic interactions modulate downstream cyclization rearrangements^20^, the current work reveals that positions 402-467-430 form a strongly epistatic residue network that coordinately controls the onset of cyclization, the defining catalytic function of the TPS enzyme family.

**Figure 4:**
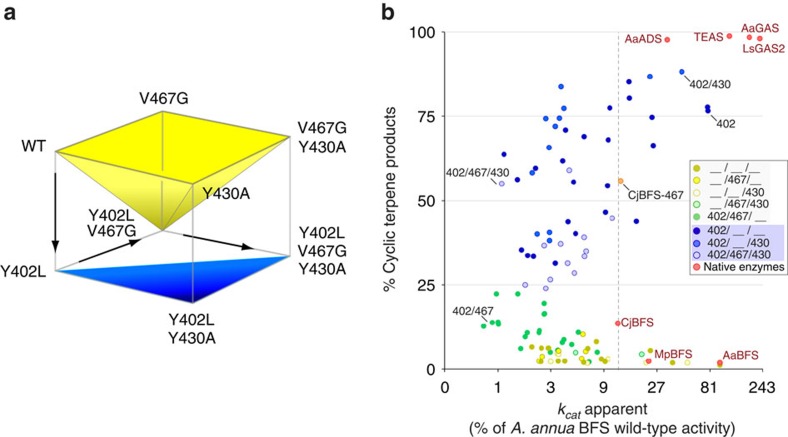
An epistatic network controls cyclization. (**a**) Cube representation of the genotypic landscape of the residue network (402, 467 and 430) that controls the onset of terpene cyclization. Each corner of the cube (node) represents a different mutational combination and is linked to its adjacent corner by the addition or removal of a mutation. Five combinations result in linear products, grouped into the yellow inverted pyramid shape. The remaining three combinations result in cyclic products and form the blue triangular plane. Arrows delineate a path from wild-type BFS to the triple mutant along a fracture plane separating linear from cyclic activities. (**b**) The impact of additional mutations on the 402-467-430 residue network was plotted to show production of cyclic products versus turnover number (*k*_cat_ apparent). The data were grouped according to the eight nodes shown in panel **a**. Yellow and green points denote linear, while blue points denote cyclic product-producing mutants.

The order of mutational steps can have a profound effect on the acquisition or disappearance of novel or existing traits, as exemplified by studies of protein evolution in antibiotic resistance^21^. To consider the functional consequences of alternative mutational pathways, we constructed the genotypic space formed by the 402-467-430 epistatic network, represented as a cube of 2^3^=8 genotypic states (Fig. 4a). While farnesene synthase specificity persists along the top plane, one can trace a hypothetical path from wild-type BFS to the triple mutant where activity oscillates between linear and cyclic products; cyclization can be activated by the dominant mutation (Y402L), suppressed by addition of a second-site suppressor mutation (Y402L, V467G) and reactivated from a suppressed state (Y402L, V467G, Y430A). Under alternative scenarios, early acquisition of V467G blocks cyclization while initial drift to Y430A primes BFS to become a catalytically robust cyclase on subsequent acquisition of the Y402L mutation.

Grouping nodes of the genotype space according to shared phenotypes suggests that this network forms a ‘fracture plane’ in sequence space at the boundary of emergent catalytic function (Fig. 4a). To assess the catalytic efficiency along the fracture plane, we measured the turnover number (*k*_cat_ apparent) of 98 mutant enzymes from our collective screening and mutagenesis studies (Fig. 4b and Supplementary Table 3). A considerable fraction of mutants (15%) had catalytic properties comparable to native enzymes from other species (defined as the activity of *Citrus junos* BFS with a *k*_cat_ apparent of 0.075 s^−1^), particularly in the Y402L background. More generally, however, accumulating numbers of mutations led to an exponential decline in catalytic efficiency (Supplementary Fig. 8), indicating that a restricted subset of viable mutational pathways extend from the Y402L mutation to diverse cyclase activities.

### Structural interpretation of the cyclization mechanism

**Figure 5:**
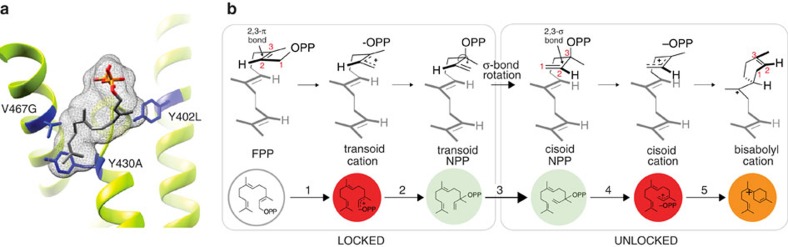
Proposed mechanism of substrate unlocking by position 402. (**a**) Spatial positions of the 402-467-430 network mapped onto a structural model of the *A. annua* BFS active site. (**b**) Proposed mechanism of substrate unlocking by the Y402L mutation involving (1) ionization of pyrophosphate, (2) recapture of pyrophosphate at C3 to form the neutral intermediate nerolidyl pyrophosphate (NPP), (3) rotation of the 2,3-σ bond, (4) re-ionization to form the cisoid carbocation and (5) 1,6 cyclization to the bisabolyl cation that parents the observed diversity of cyclic terpene products. Reaction scheme corresponds to catalytic mechanism (Fig. 1). Synthesis of cyclic products is dependent on rotation of the 2,3-σ bond of NPP (isomerization), this operation unlocks the intrinsically unproductive trans conformation of the ligand, which can then undergo cyclization to form the ubiquitous bisabolyl cation precursor of cyclic terpenes. In the absence of the Y402L mutation, isomerization cannot occur, leaving the substrate in a locked conformation only activated for the formation of linear products.

Structural models provide a basis to rationalize how the Y402L mutation may control the cyclization mechanism (Fig. 5a). To achieve the observed spectrum of cyclic products observed in BFS mutant backgrounds (Fig. 3), Y402L must both delay proton elimination and unlock the all trans FPP substrate to precisely align the ligand for cyclization along the 1,6 cyclization pathway (Fig. 5b). The close proximity of Y402L to the pyrophosphate and the first isoprene unit of FPP—the site of these key chemical transformations—suggests this residue is well-positioned to influence proton elimination, pyrophosphate ionization/recapture and 2,3 σ-bond rotation in the neutral NPP intermediate of BFS-catalysed reactions^14^ (Fig. 5a). Steric effects likely dominate, as substitution of Y402 with other aromatic residues (Phe and Trp) preserves farnesene synthase activity, while smaller aliphatic residues (Leu, Ile, Val and Thr) activate cyclization, perhaps by creating additional space for isomerization to happen more freely (Supplementary Table 7).

The physical basis for the epistatic interactions among residues of the 402-467-430 network is less obvious from models alone. The close proximity of positions Y430 to V467 suggests that these residues may interact directly, which accounts for Y430A reactivating cyclization from the V467G-suppressed background. Intriguingly, position V467 is ~10 Å away from Y402 in our structural model, adjacent to the last isoprene unit, yet exerts a profound suppressive effect on cyclization. Ultimately, the antagonistic interaction between position 402 and 467 must be transmitted either through a network of intervening amino-acid residues in the protein, through the isoprenoid chain of the bound FPP ligand or both (Fig. 5a).

### Phylogenetic context of the epistatic network

To interpret our discoveries in an evolutionary context, we conducted a phylogenetic analysis of the plant TPSs family. Examination of the data revealed that tyrosine is the ancestral state at position 402, conserved across the plant TPS-a subgroup for which the *A. annua* TPSs are cognate members (Supplementary Fig. 9). This observation suggests that the Y402L substitution was a recent event in *A. annua* and implicates Y402L as a dominant mutation in the evolutionary emergence of amorpha-4,11-diene and α-bisabolol biosynthesis, as both known dedicated TPSs contain this substitution. Consistent with this, most Y402L-contating BFS mutants in our library produce detectable amounts (~3%) of a putative amorphadiene isomer while one mutant produces α-bisabolol as the main product (61%). Considering the opposite scenario, we investigated the capacity of position 402 to suppress cyclization in ADS. While incorporating the L402Y mutation into ADS induced a broadening of cyclization products (from 94 to 82% amorpha-4,11-diene in the mutant), the more marked effect was on catalytic efficiency that was reduced by 44% (Supplementary Fig. 10). This result suggests that mutation back to the ancestral state was blocked by epistatic constraints, as numerous additional mutations undoubtedly contribute to the high-fidelity synthesis of amorpha-4,11-diene by ADS. Similar effects have been noted in evolving glucocorticoid receptors^22^ and are likely due to incompatibility from additional accumulating substitutions among divergent homologues^23^.

In a broader evolutionary context, the terpenoid synthase fold remains constant and hence the spatial positions of the 402-467-430 network residues remain essentially fixed. This raises an intriguing question of how a changing protein background influences interactions between positions within the network and their capacity to activate the cyclization mechanism. To explore this, we introduced substitutions into the equivalent positions of *C. junos* BFS from the *Rutaceae* plant family, the closest related homologue to *A. annua* BFS with 51% amino-acid sequence identity. Strikingly, G467V is able to activate cyclization; in *C. junos* G467V induces production of 15 distinct cyclic terpenes comprising 53% total product (Supplementary Fig. 11). Numerous cyclic products of *C. junos* G467V match those we previously identified for *A. annua* Y402L cyclases, indicating that cyclization proceeds via a common cisoid cyclization pathway (Fig. 1 and Fig. 3f). Importantly, G467V in *C. junos* maintains native-like catalytic efficiency (*k*_cat_/*K*_M_=2.4 versus 1.4 μM^−1^ s^−1^ for WT and G467V, respectively), indicating a viable pathway to cyclization (Fig. 4b). By contrast, position 402 alone has no impact on cyclization in the *C. junos* homologue, although pairing with position 430 promotes cyclization as seen in *A. annua* BFS (Supplementary Table 11). This result clearly demonstrates the capacity of the 402-467-430 network to control the onset of cyclization in a BFS homologue, while the changing protein background causes a marked shift in the functional contribution of individual positions.

**Figure 6:**
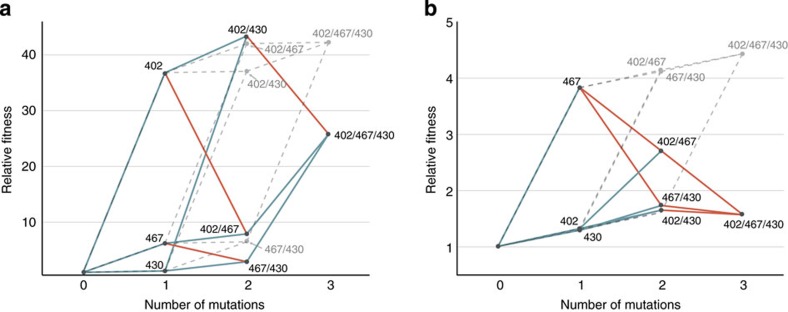
Fitness landscape analysis of the 402-467-430 residue network. The fitness effects of mutational steps within the 402-467-430 network were calculated using % cyclization as a proxy for fitness for (**a**) *A. annua* and (**b**) *C. junos* homologues relative to their respective wild type proteins (Supplementary Tables 11 and 12). Nodes correspond to mutational states, progressing from wild type to the triple mutant (as illustrated in Fig. 4a). Edges (lines) connect progressive mutational steps, where fitness gains (blue) and fitness losses (red) are indicated. A fitness peak is defined as a node with fitness gains from all incoming mutational trajectories while outgoing trajectories result in fitness loss (considered a blocked path). The corresponding epistatic-free (additive) fitness landscape is included for reference and indicated by dashed dark grey lines or as a cuboid (calculated as described in Methods).

Though qualitatively evident, we sought to quantify epistasis in *A. annua* and *C. junos* BFS backgrounds^24^,^25^. Therefore, we calculated the roughness (degree of epistasis) of the minimal fitness landscapes for the 402-467-430 network using % cyclization activity as a proxy for fitness (Table 2, Supplementary Table 12). Globally, the *A. annua* landscape was smooth (*r*/*s*=0.57) relative to other enzyme systems^26^, indicating many accessible pathways to cyclization^24^,^25^,^26^. However, the roughness of the *C. junos* landscape increased 1.5-fold (*r*/*s*=0.87), signifying a highly epistatic landscape and greater constraint on the emergence of cyclization in this protein background. To visualize epistasis, we plotted the relative fitness of all possible mutational paths across each landscape (Fig. 6). Immediately apparent is that both *A. annua* and *C. junos* networks are severely distorted as compared with the idealized additive (non epistatic) landscape, reflecting the strong epistatic character. Further, the pattern of epistatic interactions is clearly different among residues in the 402-467-430 network in each protein, providing a measure of the unique contribution of the protein background. Formally, mutations at positions 402-430 represent fitness maxima in both landscapes, appearing as global or local maxima in *A. annua* and *C. junos*, respectively. Our analysis thus reveals how interaction among residues in the 402-467-430 network, particularly the 402-430 pair, likely made a profound contribution to the emergence of cyclization in different protein backgrounds.

## Discussion

The implications of our findings paint a picture of epochal evolution in TPSs. In one possible scenario, a simple primordial BFS activity persisted while cyclization potential was continually changing via neutral drift^27^ as genes spread across different plant lineages, seeding chemodiversity to come. Then, at different times and in distinct lineages, cyclization was activated by a dominant mutation within a common epistatic network, analogous to driver nodes controlling emergent properties in complex systems^28^. The activities of the resulting, likely promiscuous, cyclase progenitor could be further honed by natural selection or refined by protein engineering^29^. The former, natural process, likely shaped a subset of TPSs in *A. annua* giving rise to amorpha-4,11-diene and α-bisabolol synthase specificities. Alternatively, one could envision a convergent evolution scenario wherein different species acquired a BFS activity via mutations that derail cyclization to linear products, as observed in bacterial TPSs^15^,^30^. To a plant, this trait may confer an advantage in warding off aphids given their highly tuned chemoreceptor for the alarm pheromone (*E)*-β-farnesene^31^, suggesting an antagonistic co-evolutionary link between insects and plants. In either scenario, a dominant mutation within an epistatic network of residues in the conserved protein fold likely drove transitions between simple and complex traits in TPSs, ultimately controlling the flow of chemical signals between organisms.

An intriguing finding in this report is the strength of interactions between distant positions in protein structure that result in the masking and unmasking of the cyclization mechanism. Distant effects have been observed in directed evolution experiments^23^,^32^, consistent with energetically coupled residues in protein structure^33^ and long range communication by allosteric interactions^34^. More interesting is the implication that epistatic interactions may be transmitted through the ligand, involving conformational re-organization of the isoprenoid chain between locked and unlocked states—the first such suggestion to our knowledge.

The preservation of epistatic linkage in the 402-467-430 network in distinct BFS backgrounds suggests that we have uncovered an intrinsic feature of the terpene synthase fold. We posit that such epistatic networks may be modular and serve as discrete control elements in protein structure akin to protein sectors^35^. Therefore, the approaches presented here could be exploited to empirically define epistatic networks through directed enzyme breeding guided by structural and phylogenetic information. Extracted networks in turn could be exploited in protein engineering applications. For example, the 402-467-430 network may activate isomerization in transoid TPS backgrounds and thereby redirect major cyclization pathways to access otherwise dormant cisoid cyclization pathways^36^ (Fig. 1). More generally, we suggest that activating or suppressing a specific catalytic function by transferring epistatic networks between homologues could be exploited in protein engineering applications to drive synthetic biology efforts for industrial and pharmaceutical biotechnology applications.

## Methods

### Homology modelling of BFS and ADS protein structures

Homology models were generated by submitting full-length amino-acid sequences to the I-TASSER server^37^,^38^. Substrate docking was carried out using AutoDock Vina^39^. Comparative sequence-structure analysis for the design of the BFS library (24 amino-acid substitutions within a 6-angstrom radius of the active site) and protein structure figures were generated using UCSF Chimera v.1.8.1 (ref. 40).

### Gene deconstruction and 3-plasmid system cloning

The BFS gene was deconstructed into three gene fragments for mutagenesis and library construction. Gene fragments were amplified by PCR using a full-length cDNA clone BFS-pH9GW as a template. PCR was carried out using Phusion High-Fidelity polymerase (New England Biolabs) for 30 cycles (program: 98 °C, 20 s, 50 °C, 30 s, 72 °C, 30 s and final extension at 72 °C, 10 min). The N-terminal, central and C-terminal fragments were ligated into pBSK2, pVL1392 and pcDNA plasmids, respectively, followed by transformation into DH5α Library Efficiency cells (Life Technologies).

### Mutagenesis of plasmid library and full-length genes

The 24 mutations were localized in six regions (zones) of the protein sequence. Each gene fragment (N-terminal, central and C-terminal) contained two zones and primers were designed to incorporate mutational combinations into each zone (Supplementary Fig. 1). Complementary primers were designed according to the QuikChange Site-Directed Mutagenesis Kit Instruction Manual (Stratagene). Non-overlapping primers were designed according to Liu and Naismith^41^ and synthesized by Sigma Aldrich; forward and reverse primers were diluted to 10 mM and mixed in a 1:1 ratio. All primers and their T_m_, T_m no_ and T_m pp_ are detailed in Supplementary Table 1. Mutagenesis reactions were designed to incorporate three increasing levels of mutagenic variation (low, medium and high) into each of the three gene fragments. Each mutagenesis reaction of 25 μl contained 10 ng of central plasmid, 0.4 μM primer mixture, 400 μM dNTPs, 4% DMSO and 0.5 units of Phusion High-Fidelity polymerase. The PCR cycles were initiated at 98 °C for 3 min to denature the template DNA, followed by 18 amplification cycles (program: 98 °C, 1 min; 50 °C, 1 min; 68 °C, 5 min (N- and C-terminal fragments)/11 min (central fragment); and final extension at 68 °C, 10 min). Following mutagenesis, 5 μl of each PCR reaction was analysed by agarose gel electrophoresis on a 1% TAE agarose gel containing 0.05% ethidium bromide. Reactions that contained a single band at the correct size were treated with 10 units of *Dpn*I at 37 °C for 2 h. An aliquot of 2 μl of each PCR product was transformed into 20 μl *E. coli* XL-10 Gold Ultracompetent cells (Agilent) by heat-shock. For individual mutants, the transformed cells were spread on a Luria-Bertani (LB) plate containing antibiotics and incubated at 37 °C overnight. For mutant mixtures, the transformed cells were added directly to liquid LB media containing antibiotics and incubated at 37 °C overnight with shaking at 230 r.p.m. Positive mutants were identified by sequencing purified plasmid DNA.

### SCOPE fragment amplification

Before BFS gene reconstruction, mutagenized N-terminal and C-terminal gene fragments were amplified by PCR using the mutagenized N- and C-terminal plasmid library mixtures as a template. Specific recombination primers and generic amplification primers were designed as described in Dokarry *et al*.^17^ (Supplementary Table 1). PCR was carried out using Phusion High-Fidelity polymerase (98 °C for 3 min, followed by 30 cycles of 98 °C for 15 s, 50 °C for 30 s, 72 °C for 1 min and 72 °C for 10 min) Each reaction was verified by agarose gel electrophoresis on a 2% TAE agarose gel, and fragments were diluted 1:10 for use in the SCOPE recombination reaction.

### SCOPE library recombination

To recombine the full-length BFS gene, diluted N-terminal and C-terminal fragments were mixed together in a 1:1 ratio and recombined with 1 nM of the central fragment plasmid. For the N- and C-terminal fragments, there were three sets of fragment mixtures with increasing levels of mutagenic variation. These were mixed in a 1:1 ratio in a grid, as shown in Supplementary Table 4b. Then 1 nM of the central fragment plasmid mixture was added to each of the N-terminal and C-terminal fragment mixtures. The reaction was set up as described in Dokarry *et al*.^17^ Recombination PCR was carried out using Phusion High-Fidelity polymerase (98 °C for 3 min, followed by 30 cycles of 98 °C for 15 s, 50–70 °C ramp (50 °C at cycle 1, then +1.5 °C per cycle) for 30 s, 72 °C for 30 s). Reactions were placed on ice and used directly in the SCOPE amplification reaction.

### SCOPE library amplification

Following gene recombination to assemble the BFS gene, the full-length gene was amplified using 2.5 μl of the recombination reaction as a template for the SCOPE amplification reaction. The reaction was set up as described in Dokarry *et al*.^17^ PCR was carried out using Phusion High-Fidelity polymerase (98 °C for 3 min, followed by 30 cycles of 98 °C for 15 s, 65 °C for 15 s, 72 °C for 1 min and 72 °C for 10 min). Reaction products were PEG precipitated into the same volume of Tris-EDTA buffer, pH 8.0 (TE buffer) before Gateway cloning.

### Cloning of individual mutants

For individual mutants, Gateway cloning was carried out in 5 μl reactions. pDONR207 and pH9GW were used as the entry vector and destination vector, respectively. A 1 μl portion of the BP or LR reaction was transformed into 10 μl *E. coli* DH5α Library Efficiency cells (Life Technologies) by heat-shock. The transformed cells were spread on LB plates containing antibiotics and incubated at 37 °C overnight. Sequencing of BP clones was used to identify correctly sequenced mutants to proceed to the LR reaction. For protein expression, pH9GW plasmids were transformed into 5 μl *E. coli* BL21(DE3) cells (NEB) by heat shock. Following cell recovery in 100 μl Super Optimal Broth (SOC), 10 μl of transformed cells were spread on LB plates containing antibiotics and incubated at 37 °C overnight.

### Cloning of mutant mixtures

For mutant mixtures, Gateway cloning was carried out in 5 μl reactions. The entire BP or LR reaction was transformed into 50 μl *E. coli* DH5α Library Efficiency cells by heat-shock. The transformed cells were added directly to liquid LB media containing antibiotics and incubated at 37 °C overnight with shaking at 230 r.p.m. For protein expression, expression plasmids were transformed into 25 μl *E. coli* BL21(DE3) cells by heat-shock. Following cell recovery in 250 μl SOC, the transformed cells were added directly to liquid LB media containing antibiotics and incubated at 37 °C overnight with shaking at 230 r.p.m.

### Protein expression of mutants (library screening)

Mutant mixtures in BL21(DE3) cells from each of the nine pools were spread onto LB plates with kanamycin, to isolate individual colonies each containing a single unique mutant. Individual colonies were transferred to 2.5 ml liquid media (LB with kanamycin) in 24-well plates and incubated overnight with shaking at 37 °C at 230 r.p.m. A 0.5 ml of each overnight culture was diluted to 5 ml with Terrific broth (TB) growth media with kanamycin in 24-well round bottom plates covered with micro-porous tape, followed by growth with shaking at 37 °C at 180 r.p.m. until cultures reached OD600≥0.8. Protein expression was induced by addition of IPTG to 0.1 mM followed by growth with shaking at 20 °C at 180 r.p.m. for 5 h. Cells were harvested by centrifugation and cell pellets were frozen at −20 °C.

### Ni-NTA chromatography purification of library proteins

Pellets from 5 ml expression cultures were re-suspended by adding 0.8 ml of lysis buffer (50 mM Tris-HCl, 500 mM NaCl, 20 mM imidazole, 10% glycerol (v/v), 10 mM β-mercaptoethanol, and 1% (v/v) Tween-20, pH 8) containing 1 mg ml^−1^ lysozyme and 1 mM EDTA directly to frozen pellets followed by shaking at room temperature at 250 r.p.m. for 30 min. Subsequently, 10 μl of benzonase solution (850 mM MgCl_2_ and 3.78 U μl^−1^ benzonase (Novagen) was added followed by additional shaking at 250 r.p.m. for 15 min. The lysate was passed through a Whatman unifilter 96-well plate and collected in another Whatman plate containing 50 μl bed-volume of superflow Ni-NTA resin (QIAgen), pre-equilibrated with lysis buffer using a vacuum manifold. Each well was washed with 1.5 ml lysis buffer (3 × 500 μl), followed by 1.5 ml wash buffer (lysis buffer lacking Tween-20). Resin was air-dried before addition of 150 μl elution buffer (wash buffer containing 250 mM imidazole), followed by centrifugation at 1,500 r.p.m. for 2 min to recover eluted protein. Protein concentration was measured using the Bradford Microassay protocol.

### Enzyme vial assay

The assay was performed as previously described^42^ in 2 ml screw-top glass vials (Agilent) in 500 μl reaction volume. Each reaction consisted of assay buffer at pH 7.0 [25 mM 2-(N-morpholino)ethanesulfonic acid (MES), 25 mM N-cyclohexyl-3-aminopropanesulfonic acid (CAPS), 50 mM Tris(hydroxymethyl)aminomethane (Tris)], 5 mM MgCl_2_, 100 μM farnesyl diphosphate (FPP) and enzyme (1.5–3 μM). Reactions were mixed and overlaid with 500 μl hexane (Sigma), and the caps were affixed. After an overnight incubation at room temperature, the hydrocarbon products were extracted by vortexing for 10 s, followed by GC–MS analysis^42^.

### Product identification and quantification by GC–MS

Reaction products were analysed using a Hewlett–Packard 6890 gas chromatograph (GC) coupled to a 5973 mass selective detector (MSD) outfitted with a 7683B series injector and autosampler and equipped with either an HP-5MS capillary column (5% diphenyl/95% dimethyl siloxane) for standard separations or an HP-Chiral-20B column (20% β-cyclodextrin) for chiral resolution (0.25 mm i.d. × 30 m; 0.25 μm film dimensions) (Agilent). The GC was operated at a He flow rate of 0.8 ml min^−1^, and the MSD was operated at 70 eV. Splitless injections of 2 μl were performed with an injector temperature of 250 °C. Initial oven temperature of the GC was 80 °C (1 min hold), which was then increased 20 °C/min up to 140 °C (1 min hold), followed by a 5 °C min^−1^ ramp until 170 °C (2 min hold), followed by a 100 °C min^−1^ ramp until 300 °C (1 min hold). A solvent delay of 6 min was allowed before the acquisition of MS data. For chiral separations, GC was operated at a He flow rate of 1.5 ml min^−1^ with an injector temperature of 200 °C. Initial oven temperature of the GC was 50 °C (5 min hold), which was then increased 10 °C min^−1^ up to 180 °C (4 min hold), followed by a 100 °C min^−1^ ramp until 240 °C (1 min hold). A solvent delay of 8.5 min was allowed before the acquisition of MS data. Product peaks were quantified by integration of peak areas using Enhanced Chemstation (version E.02.00, Agilent Technologies). Products were identified using Massfinder 4.25 (http://massfinder.com/). Identified products were compared with known terpenes in *A. annua*^43^,^44^.

The GC–MS data were inspected to identify the peaks (compounds) to be quantified in the series of samples. The quantification was carried out automatically and used the mass spectra to obtain chromatograms extracted for ions (*m*/*z*) (usually 3–5) specific to each compound. First the intensities of each extracted chromatogram were calculated using Met-Idea v2.05 (ref. 45), based on a collection of [retention time, *m*/*z*] pairs (Supplementary Table 13). The rest of the steps were carried out in Matlab 2013 (The MathWorks) using scripts written in-house. For each extracted chromatogram, the intensities were corrected to take into account the percentage signal that the ion represented in the mass spectrum, so that the corrected intensities should be the same for all ions and represent the amount compound present (relative quantitation). These intensities were averaged across ions. The percentage signal represented by each compound was then calculated. In addition a report, from scripts written in house, was generated that provided a number of useful diagnostic tools, notably graphs showing the extracted chromatograms over the relevant RT range, as well as the correlation between the corrected intensities from different ions. These were used to detect systematic bias resulting from non-specificity and/or interference between closely eluting compounds. When necessary the list of ions was refined so as to limit such occurrences.

### Malachite green assay for kinetic measurements

Kinetic assays were performed as described^18^ using 96-well flat-bottomed plates (Grenier). For *k*_cat_ apparent measurements, assays of 50 μl were conducted in malachite green assay buffer (vial assay buffer containing 2.5 mU of the coupling enzyme inorganic pyrophosphatase) from *C. cerevisiae* (Sigma) using six twofold serial dilutions of purified protein. Monophosphate (Pi) and pyrophosphate (PPi) standard curves (100 to 0.01 μM) were generated using a twofold serial dilution in malachite green assay buffer without FPP. Reactions were set up in duplicate and incubated at room temperature for 30, 90 and 180 min. Enzyme reactions were quenched by addition of the malachite green development solution (prepared according to Pegan *et al*.^46^), incubated for 15 min and then read at 623 nm on a Varioskan Flash plate reader^18^.

For steady-state kinetic measurements, assays of 50 μl were conducted in malachite green assay buffer, and serial dilutions of FPP, with a starting concentration of 100 μΜ. Enzyme was added to give a final concentration of 0.014 μΜ, unless otherwise stated. Standard curves of monophosphate (Pi) and pyrophosphate (PPi) (50 to 0.01 μM) were generated as above. Reactions were set up on ice in triplicate and incubated at 30 °C for 15 or 40 min, depending on the mutant studied. Enzyme reactions were quenched and read at 623 nm as above.

### Calculations of minimal fitness landscapes

We quantified the magnitude of epistatic interactions between substitutions through a commonly used measure of roughness^24^,^47^. A pure additive limit was calculated using a multidimensional linear model^26^. A convenient measure of epistasis is a ratio of roughness to slope. This ratio measures how well the landscape can be described by a linear model, which corresponds to the purely additive (or non-epistatic) landscape. A multidimensional linear model was fitted to our fitness landscapes by means of a least-squares fit. The ruggedness was calculated via the roughness to slope ratio (*r*/*s*), using the linear model as described by Szendro *et al*.^26^ Each mutant was represented by a binary string **b**=(*β*_1_,*β*_2_,…,*β*_*L*_) of length *L*, where *β*_*i*_=0(1) if the mutation at the *i*-th position is absent or present. The model is:


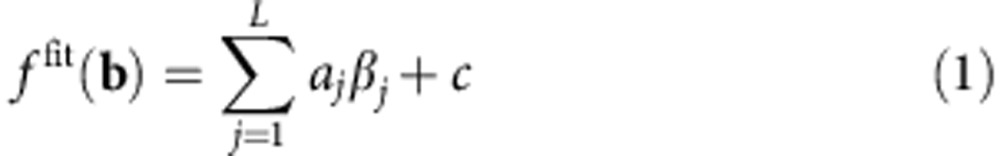


where the fitting parameters are *c* and *a*_*j*_ (coefficients). The mean slope, equation (2), was found by averaging the absolute of the fitted coefficients *a*_*j*_’**s**:


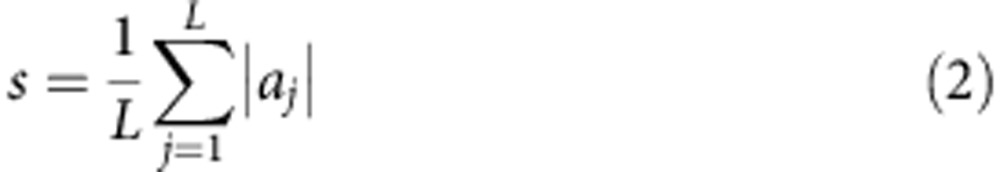


and the roughness, equation (3), was defined as:


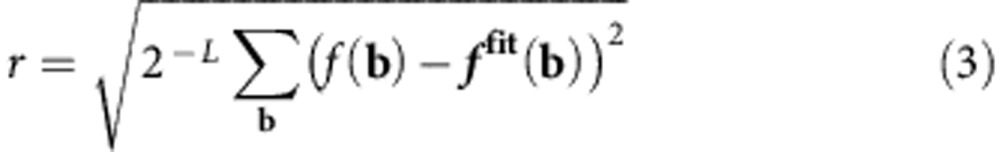


The higher the *r/s* ratio, the higher the roughness, which leads to more deviation from the linear model and suggesting that more epistasis is present in the landscape. For a purely additive smooth landscape, *r/s*=0. We have used the scaled values of cyclization percentage as a proxy for fitness. We compare the roughness and the *r/s* ratio between *A. annua* BFS and *C. junos* BFS in Table 2.

### Fourier analysis of fitness landscapes

Fourier analysis was performed using the formulation by Szendro *et al*.^26^ and Zanini and Neher^48^. A function that maps a binary string **b** to fitness can be written as an expansion in terms of main effects and interaction between the three sites:


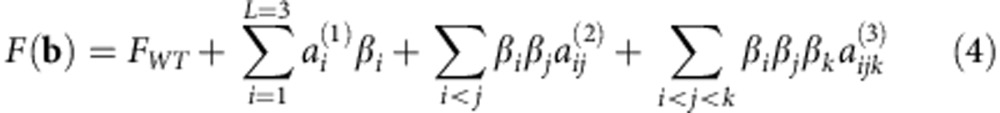


where *β*_*i*_ε{0,1} denotes the mutation variant to indicate the absence or presence of a mutation at a site. The first order coefficient *a*^(1)^ in the expansion captures the linear, non epistatic effects. The second order coefficient *a*^(2)^ describes the pairwise epistatic interaction and *a*^(3)^ denotes three-way epistatic interaction. In total there are 2^*L*^ coefficients, we can simply find them by solving a system of linear equations. We used the relative fitness values for the estimation of various epistatic measures and to infer coefficients of the expansion (Supplementary Table 12). Coefficients calculated in the *β*_*i*_ε{0,1} basis were used to find the epistatic free reference landscape by summing-up only the constant and first order terms while predicting the fitted landscape (Fig. 6), equivalent to setting the second and third order coefficients to vanishingly small or zero. We also calculated the coefficients of the transform in the *β*_*i*_ε{−1,+1} basis to calculate quantities *F*_*sum*_, *F*_1_, *F*_2_ and *F*_3_ as defined in Szendro *et al*.^26^ For a purely additive, non-epistatic landscape we expect that *F*_*sum*_=0 and *F*_1_=1. The fitted Fourier coefficients and contribution of epistasis orders are listed in Supplementary Table 12.

## Author contributions

M.S. synthesized, expressed, purified and characterized the BFS 6 Å library, carried out additional mutagenesis and performed data analysis; C.L. expressed, purified and characterized the BFS 6 Å library; M.S. and C.L. optimized the experimental process; M.V. performed the steady-state kinetic measurements; J.C. performed computational analysis of epistasis, constructed figures and wrote the paper; M.D. designed software and performed GC–MS bioinformatics analysis; S.G. was involved in study design and performed phylogenetic analyses; J.A.F. analysed the data and provided interpretation of the reaction mechanism; P.E.O. and M.S. designed the study, analysed the data, constructed figures and wrote the paper. All authors discussed the results and commented on the manuscript.

## Additional information

**How to cite this article**: Salmon, M. *et al*. Emergence of terpene cyclization in *Artemisia annua*. *Nat. Commun.* 6:6143 doi: 10.1038/ncomms7143 (2015).

## Supplementary Material

Supplementary Information Supplementary Figures 1-11 Supplementary Tables 1-13 and Supplementary References

## Figures and Tables

**Table 1:** Steady-state kinetic parameters for selected mutants along a mutation path of the epistatic cube.

**Name**	***K***_**m**_ **(μM)**	***k***_**cat**_ **(s**^**−1**^**)**	***k***_**cat**_***/K***_**m**_ **(μM**^**−1**^ **s**^**−1**^**)**
BFS (WT)	16.19	0.170	10.5 × 10^**−**3^
Y402L	10.57	0.062	5.9 × 10^**−**3^
Y402L/V467G	5.99	0.005	0.8 × 10^**−**3^
Y402L/V467G/Y430A	4.27	0.004	0.9 × 10^**−**3^
Y402L/Y430A	5.22	0.037	7.1 × 10^**−**3^

**Table 2:** Fitness landscape properties for *A. annua* and *C. junos.*

**Landscape property**	***A. annua***	***C. junos***
Roughness (*r*)	7.792	0.567
Mean slope(*s*)	13.64	0.651
Roughness to mean slope ratio (*r*/*s*)	0.571	0.871
Correlation coefficient	0.873	0.760
