# Supplementary material for: Emergence of terpene cyclization in Artemisia annua
Source: Nat Commun. 2015 Feb 3;6:6143. doi: 10.1038/ncomms7143 (PMC4327562; doi:10.1038/ncomms7143)
Supplement: Supplementary Information — Supplementary Figures 1-11 Supplementary Tables 1-13 and Supplementary References [file ncomms7143-s1.pdf]

ZONE 1

Zone 1: 296

	287	288	289	290	291	292	293	294	295	296	297	298	299	300	301	302	303	304	305	306
**AaADS**	ccg	tgc	ctg	cgt	gac	cgt	atc	gtt	gaa	tgt	tac	ttc	tgg	ggt	ctg	ggt	tct	ggt	tat	gaa
	P	C	L	R	D	R	I	V	E	C	Y	F	W	G	L	G	S	G	Y	E
**AaFS**	P	Y	V	R	D	R	V	V	E	G	Y	F	W	I	L	S	I	Y	Y	E
	cct	tat	gtt	cga	gac	agg	gtg	gtg	gaa	ggc	tat	ttt	tgg	ata	cta	tcc	atc	tat	tac	gag
N1.11				R	D	R	V	V	E	C	Y	F	W							
G296C-F								V	E	C	Y	F	W	I	L	S	I	Y	Y	E
G296C-R	P	Y	V	R	D	R	V	V	E	C	Y	F	W							

ZONE 2

Zone 2: 319–325

	313	314	315	316	317	318	319	320	321	322	323	324	325	326	327	328	329	330	331
**AaADS**	cgt	gtg	ttc	ttc	act	aaa	gct	gta	gct	gtt	atc	acc	ctg	atc	gat	gac	act	tac	gat
	R	V	F	F	T	K	A	V	A	V	I	T	L	I	D	D	T	Y	D
**AaFS**	R	M	F	L	M	K	T	C	M	W	L	V	V	L	D	D	T	F	D
	aga	atg	ttc	cta	atg	aaa	aca	tgc	atg	tgg	tta	gtc	gtt	tta	gat	gat	aca	ttt	gat
N2.2	R	M	F	F	T	K	T	C	M	W	L	V	V	L	D				
N2.3	R	M	F	F	M	K	A	C	M	W	L	V	V	L	D				
N2.4	R	M	F	L	T	K	T	V	M	W	L	V	V	L	D				
N2.5	R	M	F	L	M	K	T	C	A	W	L	V	V	L	D				
N2.6	R	M	F	L	M	K	T	C	M	V	L	V	V	L	D				
N2.7	R	M	F	L	M	K	A	V	M	W	L	V	V	L	D				
N2.8	R	M	F	L	M	K	A	C	A	W	L	V	V	L	D				
N2.9	R	M	F	L	M	K	A	C	M	V	L	V	V	L	D				
N2.10	R	M	F	L	M	K	T	V	A	W	L	V	V	L	D				
N2.11	R	M	F	L	M	K	T	V	M	V	L	V	V	L	D				
N2.12	R	M	F	F	T	K	T	C	A	V	L	V	V	L	D				
N2.13	R	M	F	F	M	K	A	V	A	W	L	V	V	L	D				
N2.14	R	M	F	L	T	K	A	V	M	V	L	V	V	L	D				
N2.15	R	M	F	L	M	K	A	C	A	V	L	V	V	L	D				
N2.16	R	M	F	L	M	K	T	V	A	V	L	V	V	L	D				
N2.17	R	M	F	L	M	K	A	V	A	V	L	V	V	L	D				
N2.18					M	K	T	C	M	W	L	V	V	L	D	D	T	F	D
N2.19					M	K	T	C	M	W	I	V	V	L	D	D	T	F	D
N2.20					M	K	T	C	M	W	L	T	V	L	D	D	T	F	D
N2.21					M	K	T	C	M	W	L	V	L	L	D	D	T	F	D
N2.22					M	K	T	C	M	W	I	T	V	L	D	D	T	F	D
N2.23					M	K	T	C	M	W	I	V	L	L	D	D	T	F	D
N2.24					M	K	T	C	M	W	L	T	L	L	D	D	T	F	D
N2.25					M	K	T	C	M	W	I	T	L	L	D	D	T	F	D

ZONE 3

Zone 3: 398–402

	391	392	393	394	395	396	397	398	399	400	401	402	403	404	405	406	407	408	409	410
**AaADS**			gag	ttt	gtt	aaa	gaa	ttc	gta	cgt	aac	ctg	atg	gtt	gaa	gct	aaa	tgg	gct	aac
			E	F	V	K	E	F	V	R	N	L	M	V	E	A	K	W	A	N
**AaFS**			E	M	A	K	E	L	V	R	N	Y	L	V	E	A	R	W	L	K
			gag	atg	gca	aaa	gag	ttg	gtt	cgc	aat	tac	ttg	gta	gaa	gcc	aga	tgg	cta	aaa
T3.1			E	M	A	K	E	L	V	R	N	Y	L	V	E	A	R			
T3.2			E	M	A	K	E	F	V	R	N	Y	L	V	E	A	R			
T3.3			E	M	A	K	E	L	V	R	N	L	L	V	E	A	R			
T3.4			E	M	A	K	E	F	V	R	N	L	L	V	E	A	R			
Y402L-F										R	N	L	L	V	E	A	R	W	L	K
Y402L-R				M	A	K	E	L	V	R	N	L	L	V						
Y402FL-R				M	A	K	E	F	V	R	N	L	L	V						
402WT-F										R	N	Y	L	V	E	A	R	W	L	K
402WT-R				M	A	K	E	L	V	R	N	Y	L	V						
402WT+F-R				M	A	K	E	F	V	R	N	Y	L	V						

Zone 3: 398–402

	391	392	393	394	395	396	397	398	399	400	401	402	403	404	405	406	407	408	409	410	
**CjFS**	gca	aaa	cag	atg	atg	caa	gaa	tta	att	ata	ttg	tac	ttt	acc	gaa	gcc	aag	tgg	tta	tat	aaa
	A	K	Q	M	M	Q	E	L	I	I	L	Y	F	T	E	A	K	W	L	Y	K
CjFS-402-F									I	I	L	L	F	T	E	A	K	W	L	Y	K
CjFS-402-R	A	K	Q	M	M	Q	E	L	I	I	L	L	F	T							

ZONE 4

Zone 4: 429–433

	421	422	423	424	425	426	427	428	429	430	431	432	433	434	435	436	437	438
**AaADS**	gac	ccg	gtt	gtt	atc	atc	acc	ggc	ggt	gca	aac	ctg	ctg	acc	acc	act	tgc	tat
	D	P	V	V	I	I	T	G	G	A	N	L	L	T	T	T	C	Y
**AaFS**	M	S	V	S	M	V	T	G	T	Y	G	L	M	I	A	R	S	Y
	atg	tct	gtg	tca	atg	gtg	act	ggt	acc	tat	ggc	ttg	atg	ata	gcg	aga	tct	tat
T4.1				S	M	V	T	G	T	Y	G	L	M	I	A	R	S	Y
T4.2				S	M	V	T	G	G	Y	G	L	M	I	A	R	S	Y
T4.3				S	M	V	T	G	T	A	G	L	M	I	A	R	S	Y
T4.4				S	M	V	T	G	T	Y	G	L	L	I	A	R	S	Y
T4.5				S	M	V	T	G	G	A	G	L	M	I	A	R	S	Y
T4.6				S	M	V	T	G	G	Y	G	L	L	I	A	R	S	Y
T4.7				S	M	V	T	G	T	A	G	L	L	I	A	R	S	Y
T4.8				S	M	V	T	G	G	A	G	L	L	I	A	R	S	Y
T429G-F							T	G	G	Y	G	L	M	I	A	R	S	Y
T429G-R	M	S	V	S	M	V	T	G	G	Y	G							
T429GL-F							T	G	G	Y	G	L	L	I	A	R	S	Y
T419GAL-F								G	G	A	G	L	L	I	A	R	S	Y
T429GAL-R	M	S	V	S	M	V	T	G	G	A	G							
Y430WT-F							T	G	T	Y	G	L	M	I	A	R	S	Y
Y430WT-R	M	S	V	S	M	V	T	G	T	Y	G							
Y430A-F							T	G	T	A	G	L	M	I	A	R	S	Y
Y430A-R		S	V	S	M	V	T	G	T	A	G							
Y430AL-F							T	G	T	A	G	L	L	I	A	R	S	Y
Y430GA-F							T	G	G	A	G	L	M	I	A	R	S	Y
Y430GA-R		S	V	S	M	V	T	G	G	A	G							

Zone 4: 429–433

	421	422	423	424	425	426	427	428	429	430	431	432	433	434	435	436	437	438
**CjFS**	aaa	tca	gtt	gca	tta	aga	agt	atc	ggt	tta	cgt	acg	cta	gcg	gtc	gct	tcc	ttc
	K	S	V	A	L	R	S	I	G	L	R	T	L	A	V	A	S	F
CjFS-430-F								I	G	A	R	T	L	A	V	A	S	
CjFS-430-R	K	S	V	A	L	R	S	I	G	A	R	T						

Supplementary Figure 1. Schematic representation of mutations incorporated into zones 1-6 by each oligonucleotide pair Each panel shows the nucleotide and amino acid sequence of the region surrounding each 6Å zone. Each zone is labelled in grey and the residues within each zone that are in the 6Å sphere are highlighted in grey (*A. annua* BFS), orange (ADS) or blue (*C. junos* BFS). The numbers above each codon/amino acid denote the amino acid position in *A. annua* BFS. Listed below each translated sequence alignment are the set of oligonucleotides designed to incorporate different combinations of mutations into *A. annua* BFS. Complementary oligonucleotides were designed for initial mutagenesis of the 6Å library according to QuikChange™ Site-Directed Mutagenesis System developed by Stratagene (La Jolla, CA). For deconvolution mutagenesis oligonucleotides containing non-overlapping regions at their 3' ends were designed according to Liu & Naismith[1]. The full list of oligonucleotides used for both rounds of mutagenesis are shown in Supplementary Table 1.

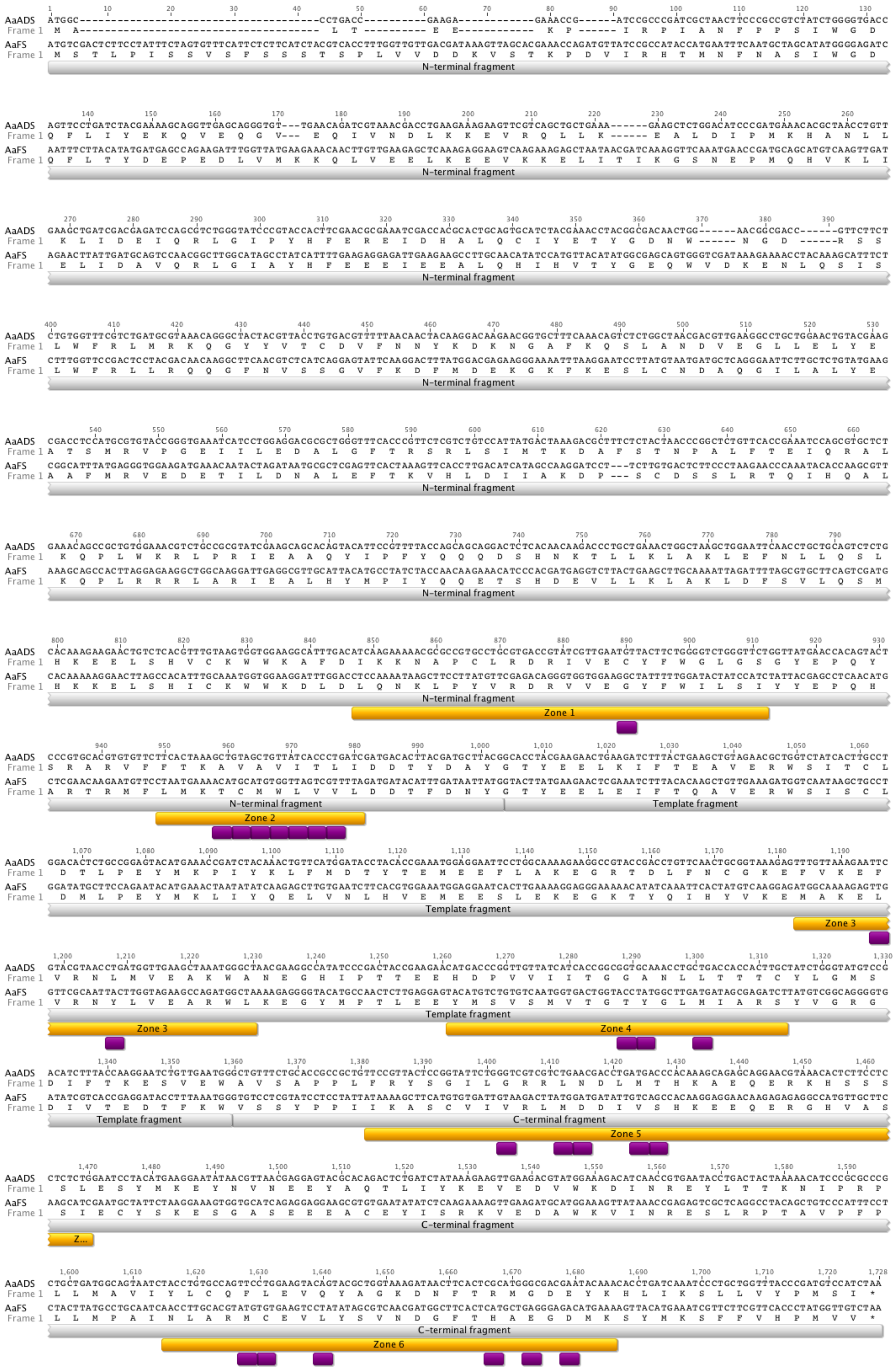

Supplementary Figure 2. Nucleotide translated sequence alignment of BFS and ADS

The alignment was generated by MUSCLE and drawn in Geneious. The sesquiterpene synthase sequences aligned are *Artemisia annua* (E)-β-farnesene synthase (BFS) and amorphadiene synthase (ADS). The gene fragments used to create the 3-plasmid system are highlighted in grey, the amino acids within 12Å of the active site are highlighted in yellow and the non-conserved amino acids within 6Å of the active site which were targeted for mutagenesis are highlighted in purple.

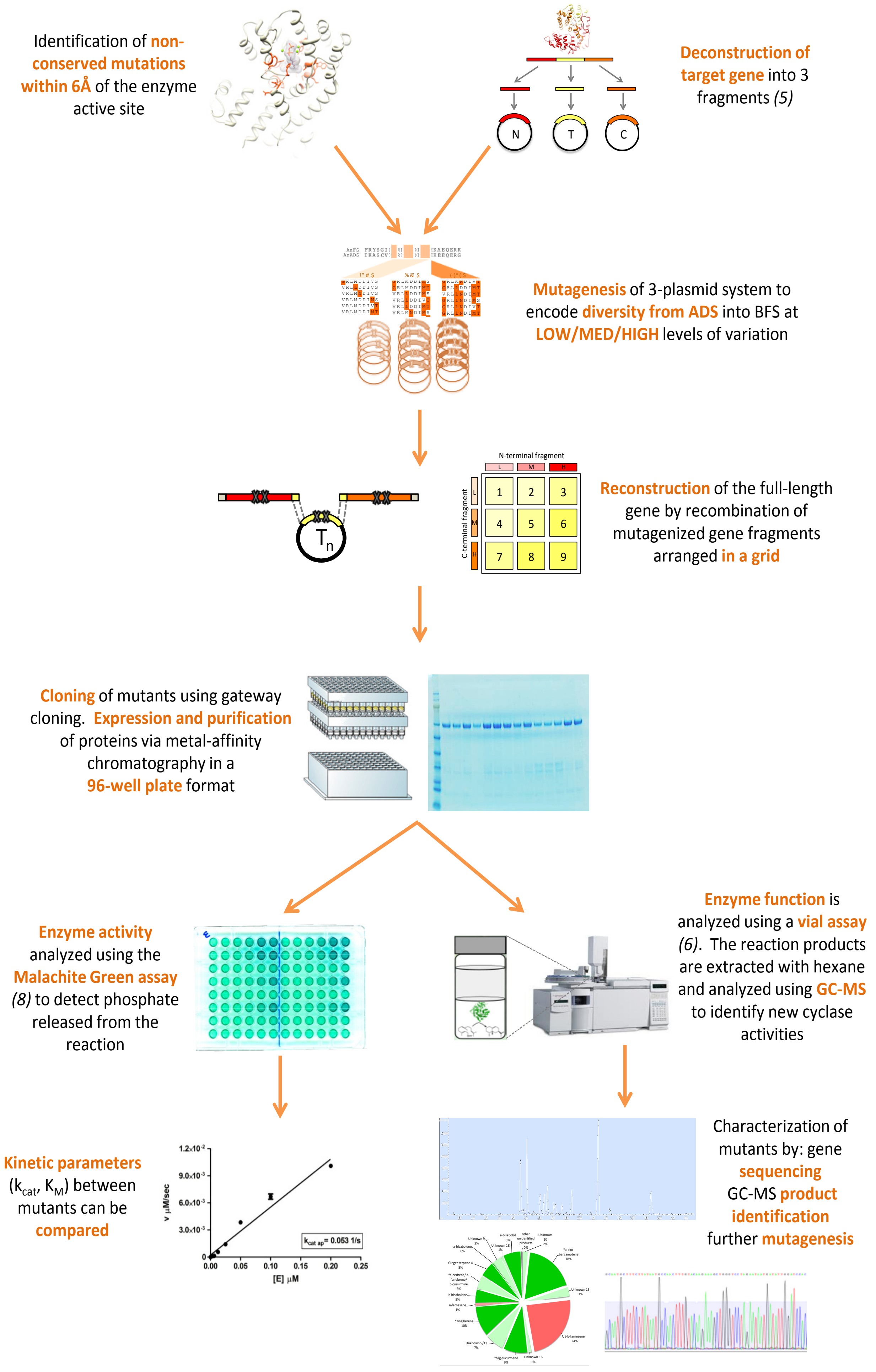

Supplementary Figure 3. Process overview of library design, synthesis and screening

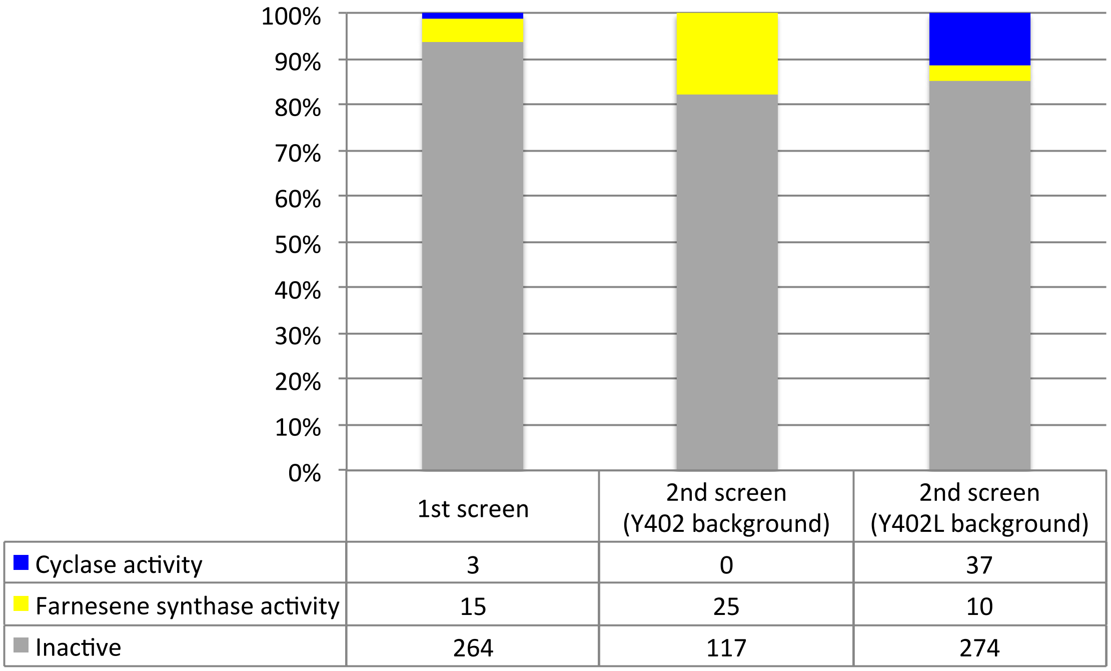

	1st screen	2nd screen (Y402 background)	2nd screen (Y402L background)
Cyclase activity	3	0	37
Farnesene synthase activity	15	25	10
Inactive	264	117	274

Supplementary Figure 4. Graphical representation of BFS 6Å library screening results
Percentage stacked column of BFS 6Å library screening results. The first screen of 282 mutants identified 3 novel cyclases. Further screening in the Y402 (WT) background did not yield any additional cyclases, whereas screening in the Y402L background resulted in the discovery of 37 novel cyclases.

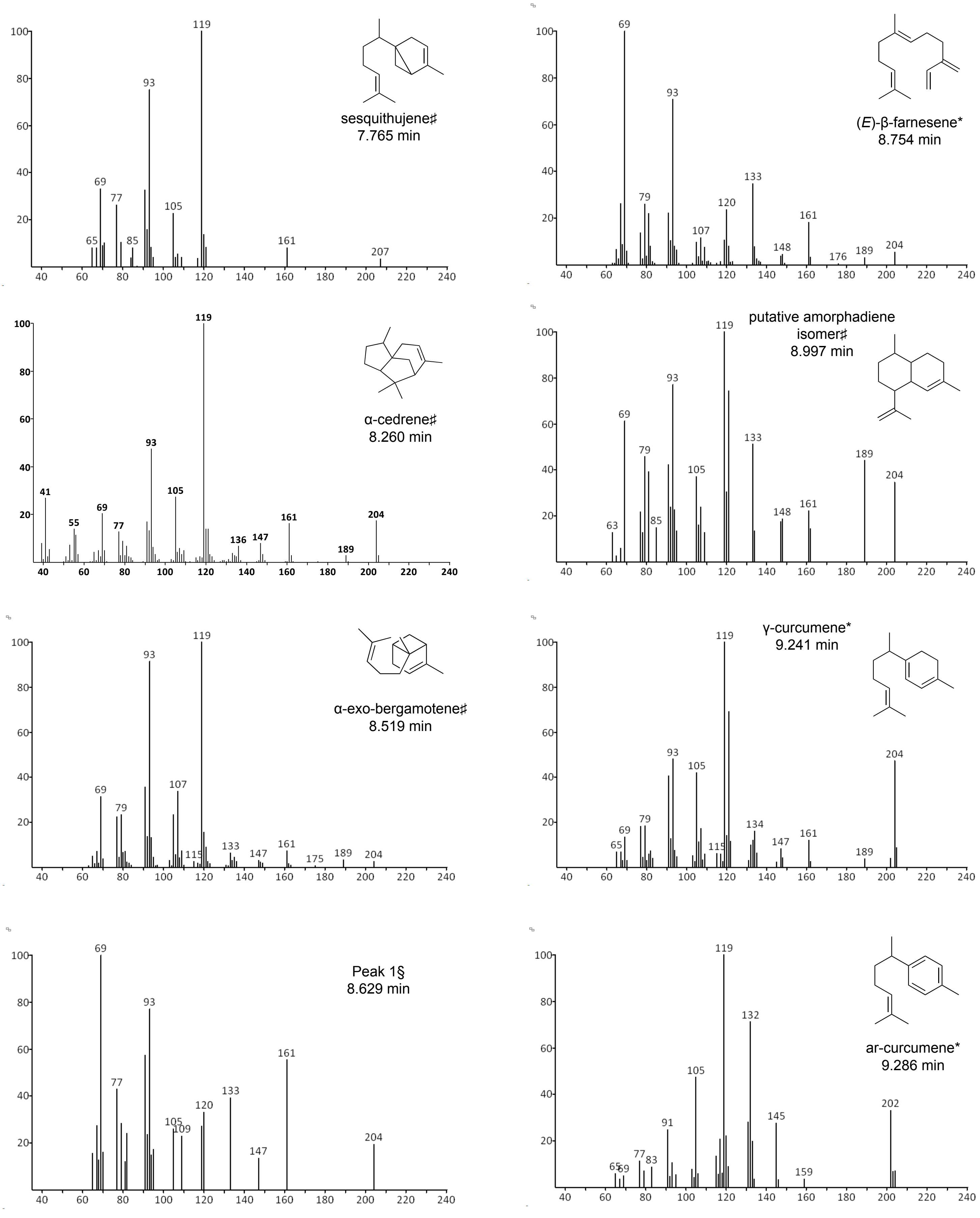

Supplementary Figure 5. Mass spectra of all products synthesized by the BFS mutant library
Mass spectrum, product name and retention time for each product synthesised by the BFS mutant library. For each product the best representative spectrum was chosen and product names were assigned by comparisons to authentic standards (*), spectra match from NIST or Massfinder libraries (♯) or labelled as unknown products (§).

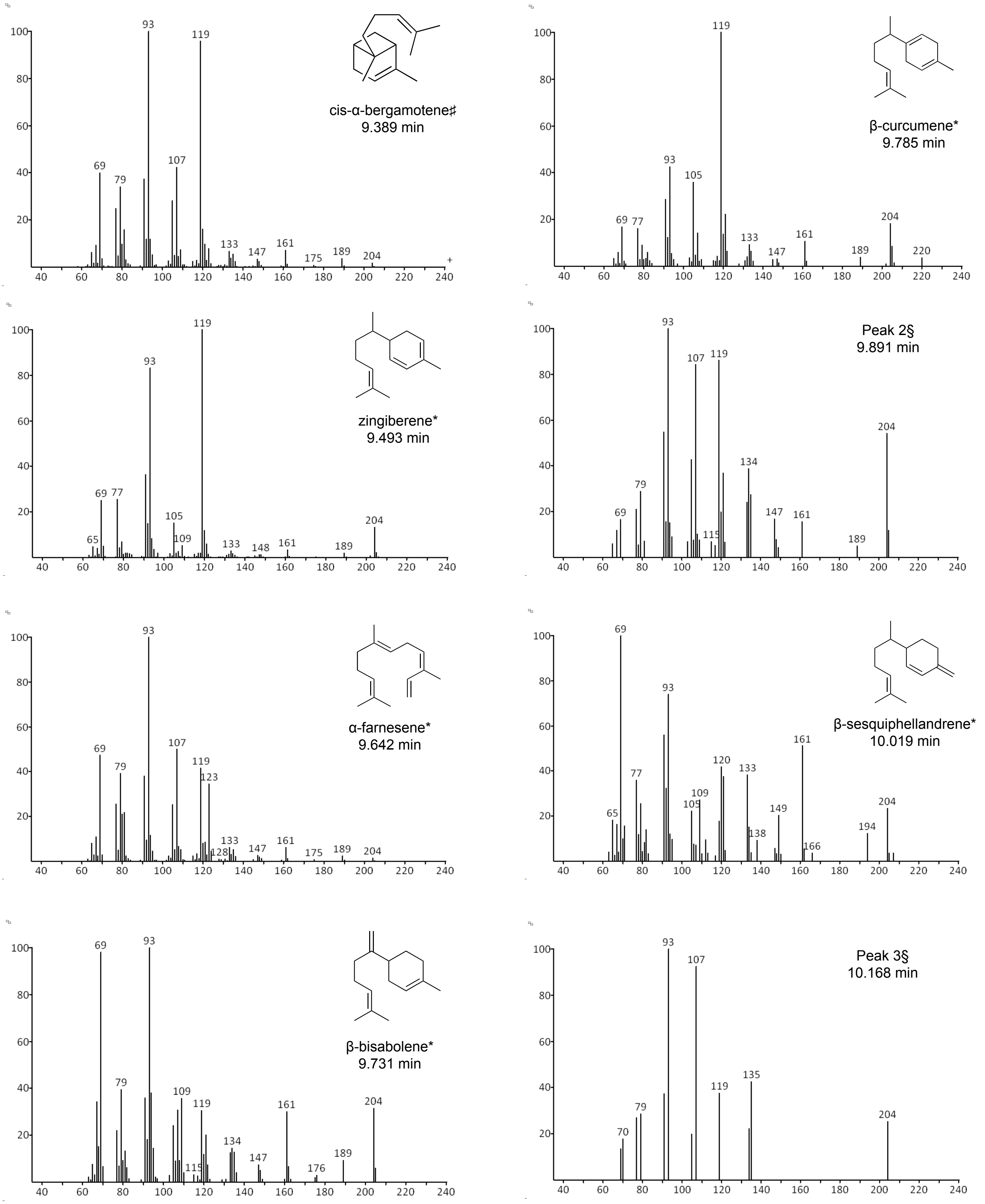

Supplementary Figure 5 continued
Mass spectrum, product name and retention time for each product synthesised by the BFS mutant library. For each product the best representative spectrum was chosen and product names were assigned by comparisons to authentic standards (*), spectra match from NIST or Massfinder libraries (♯) or labelled as unknown products (§).

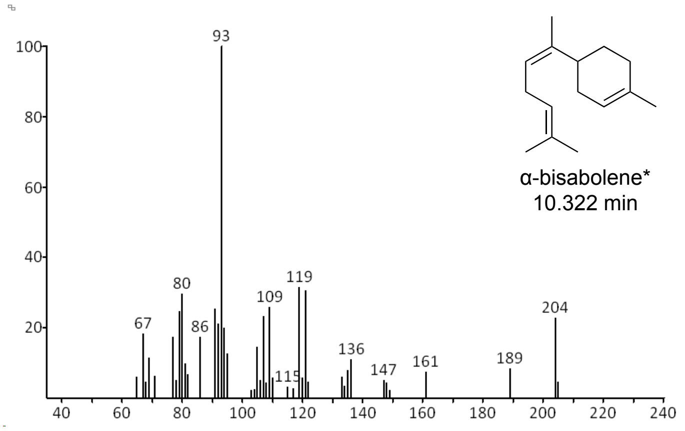

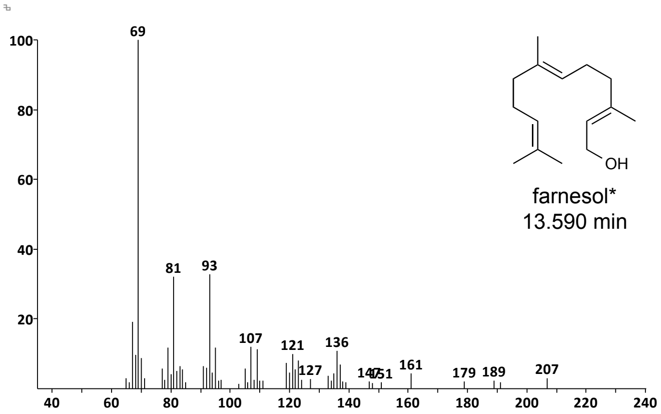

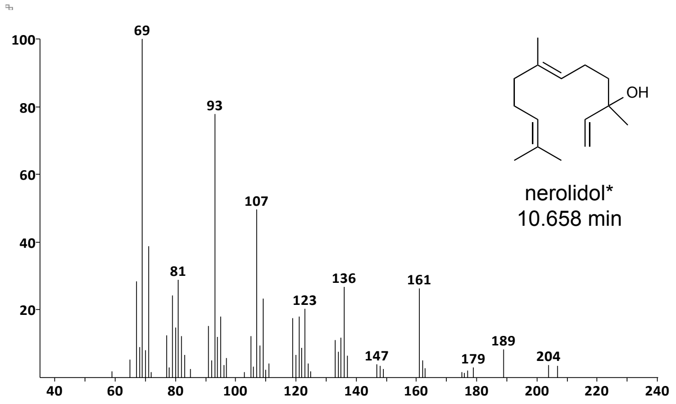

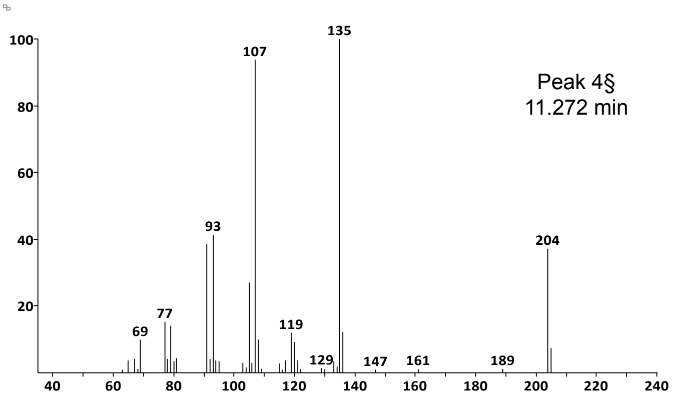

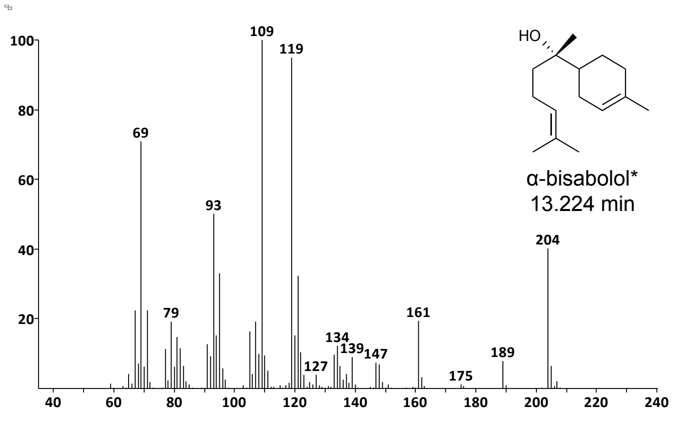

Supplementary Figure 5 continued

Mass spectrum, product name and retention time for each product synthesised by the BFS mutant library. For each product the best representative spectrum was chosen and product names were assigned by comparisons to authentic standards (*), spectra match from NIST or Massfinder libraries (♯) or labelled as unknown products (§).

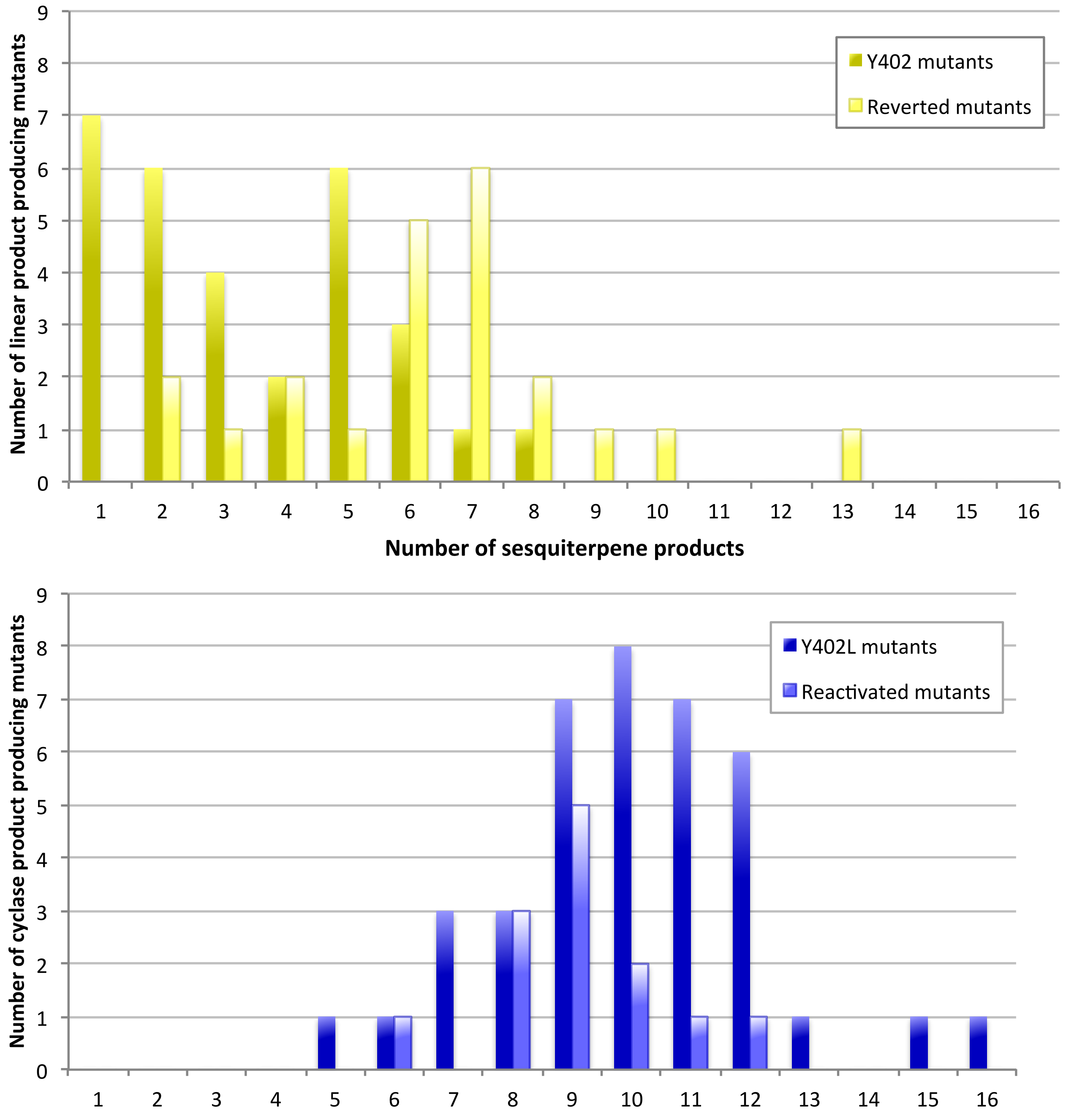

Supplementary Figure 6. Histogram of sesquiterpene products made by BFS 6Å library mutants
Distibution of the total number of sesquiterpene products made by mutants from the BFS 6Å library with backgrounds that give rise to a linear product (Y402 and reverted mutants – top panel) and a cyclic product activity (Y402L, reactivated mutants – bottom panel). Sesquiterpene product total includes both linear and cyclic sesquiterpene products over 1% of total product levels. Cyclic product producing mutants synthesize a higher diversity of products ranging from 5 to 16. Linear product producing mutants have much lower product diversity, ranging from 1-8 for 402- mutants to 2-13 for reverted mutants.

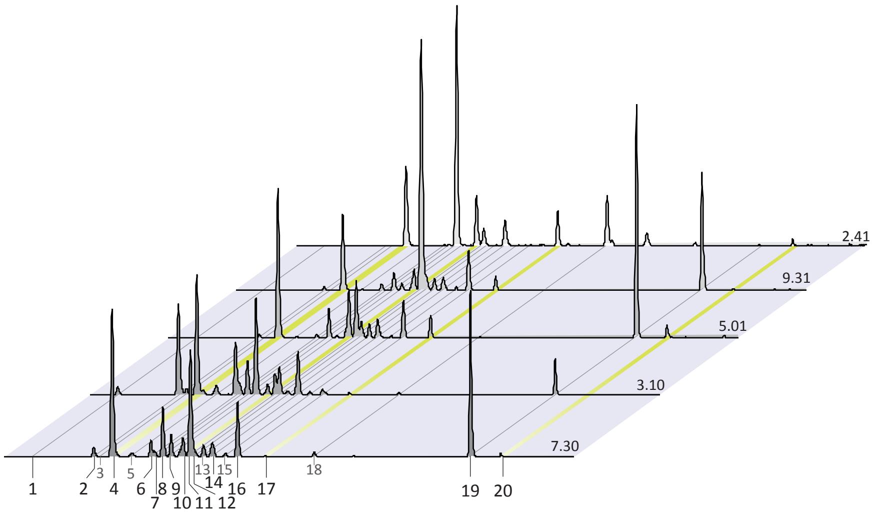

Supplementary Figure 7. Product diversity of terpenes synthesized by the BFS 6Å library

Total ion chromatograms of a representative panel of mutants from the BFS 6Å library. These include multi-product enzymes, 7.30 and 3.10, making up to 20 terpene products, and highly specific enzymes: 5.01, 9.31 and 2.41 which produce α-bisabolol; cis-α-bergamotene and β-bisabolene as their major products respectively. Linear products are indicated with a yellow line, all other products are cyclic. Labeled peaks and chemical structures were either identified by MS comparisons with authentic standards or matched to the mass spectra of compounds reported in the masssfinder library (Supplementary Figure 5 and Supplementary Table 5). 1, sesquithujene; 2, α-exo-bergamotene; 3, Peak 1; 4, (E)-β-farnesene; 5, putative amorphadiene isomer; 6, γ-curcumene; 7, ar-curcumene; 8, cis-α-bergamotene; 9, zingiberene; 10, α-farnesene; 11, β-bisabolene; 12, β-curcumene; 13, Peak 2; 14, β-sesquiphelladrene; 15, Peak 3; 16, α-bisabolene; 17, nerolidol; 18, Peak 4; 19, α-bisabolol; 20, farnesol; 21, α-cedrene; 22, acoradiene; 23, 8-epi-cedrol.

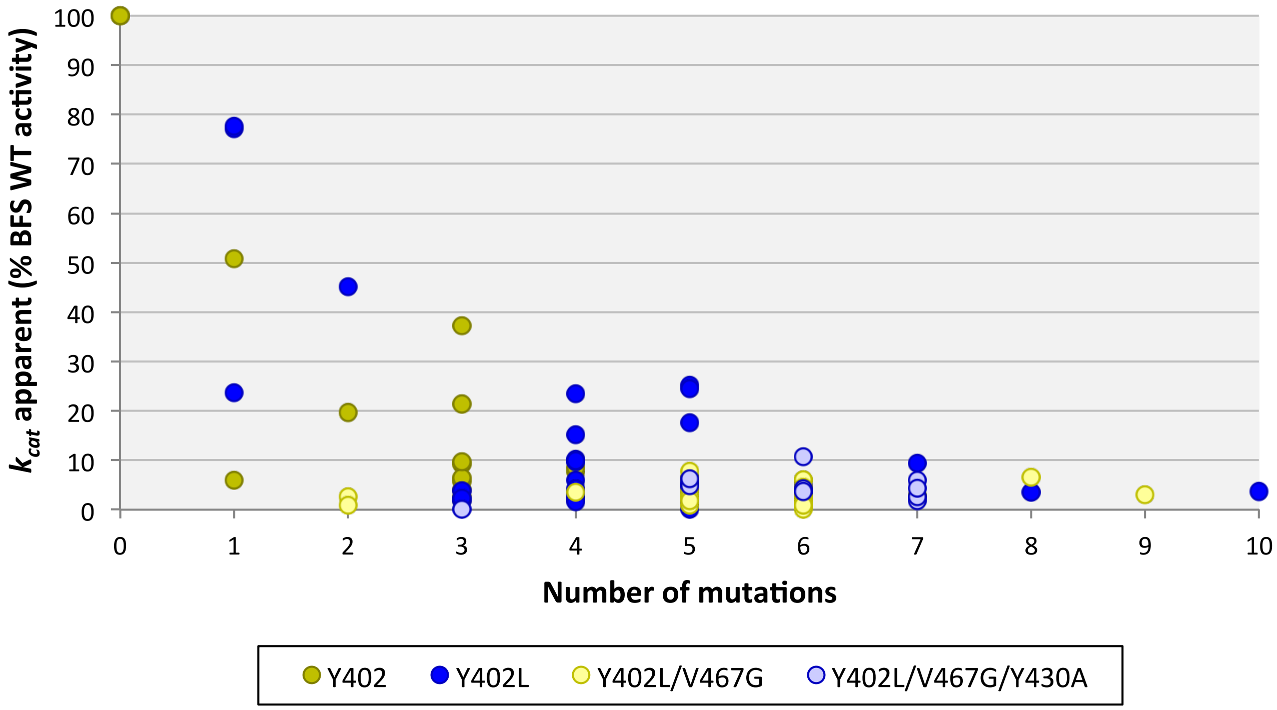

Supplementary Figure 8. Effect of increasing mutation number of k_{cat} apparent

Scatter graph to show how k_{cat} apparent for each BFS mutant is affected by increasing mutation number. The data is grouped according to 4 main mutant phenotypes shown above. Dark yellow or yellow points denote linear product producing mutants and blue or light blue points denote cyclic product producing mutants. The k_{cat} apparent values are shown as a percentage of the wild-type BFS activity.

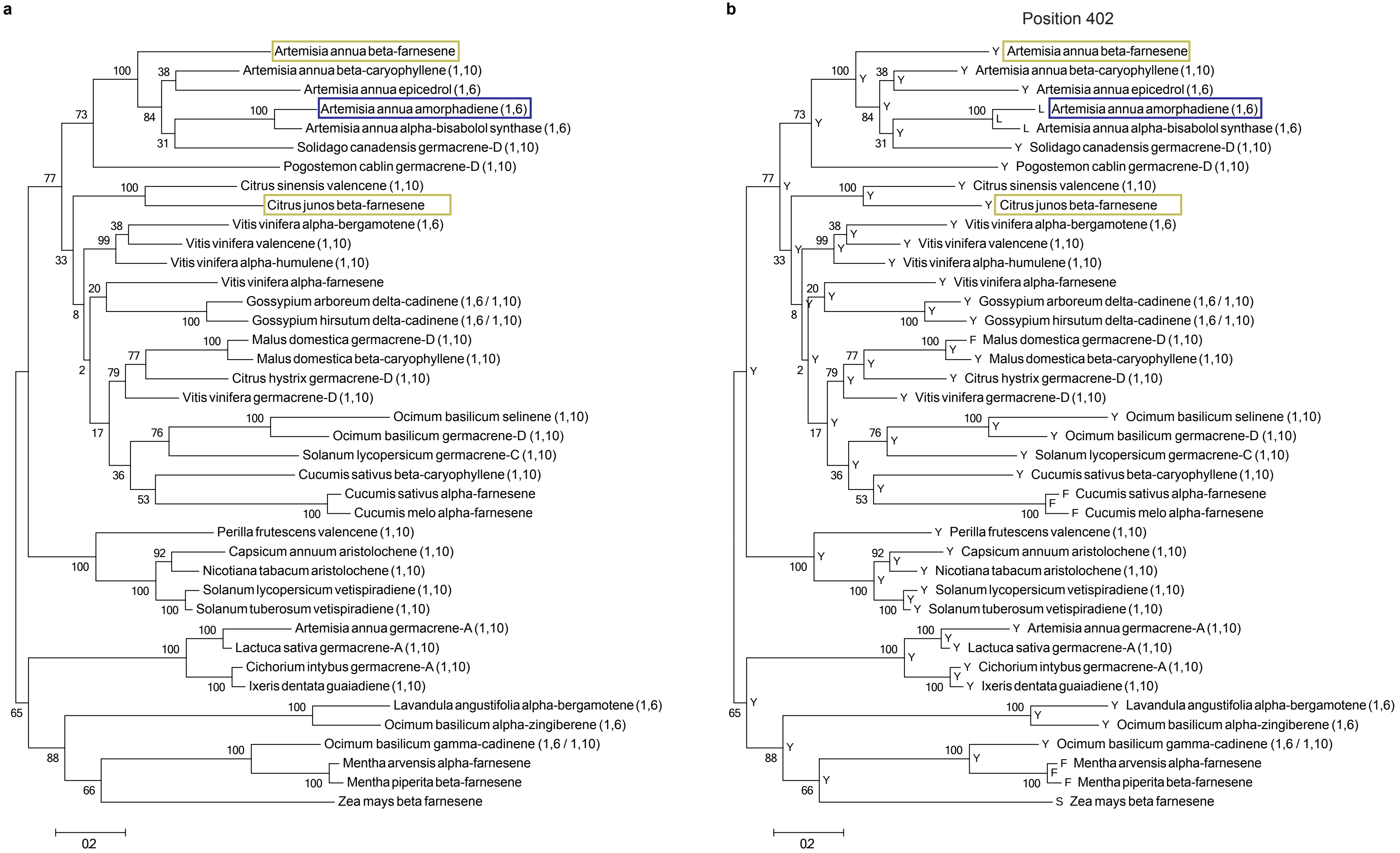

Supplementary Figure 9. Phylogenetic analysis of TPS protein sequences

Maximum Likelihood phylogenetic analysis of **a.** selected full-length protein sequences, from the TPS-a subgroup, obtained from NCBI. Current and derived ancestral assignments for amino acids equivalent to the *A. annua* amorphadiene synthase 402, 430 and 467 positions are shown in panels **b** to **d** respectively. Bootstrap values are shown as a percentage of 500 replicates. Numbers in parentheses indicate initial cyclization mechanism. The scale bar represents 0.2 substitutions per site. GeneBank accession numbers are: *Artemisia annua*: alpha-bisabolol (AFV40969), amorphadiene (CAB94691), beta-caryophyllene (AAL79181), beta-farnesene (AAX39387), epi cedrol (CAC08805) germacrene-A (ABE03980); Capsicum annuum: aristolochene (CAA06614); Cichorium intybus: germacrene-A (AAM21658); Citrus hystrix: germacrene-D (ADX01384); Citrus junos: beta-farnesene (AAK54279); Citrus sinensis: valencene (AAQ04608); Cucumis sativus: alpha-farnesene (AAU05951), beta-caryophyllene (AAU05952); Cucumis melo: alpha-farnesene (ABX83201); Gossypium arboretum: delta-cadinene (CAA65289); Gossypium hirsutum: gamma-cadinene (P93665); Ixeris dentate: guaiadiene (AAL92481) Lactuca sativa: germacrene-A (AAM11626); Mentha arvensis alpha-farnesene (ADC92564); Lavandula angustifolia: alpha-bergamotene (Q2XSC4) Mentha x piperita beta-farnesene (AAB95209); Malus domestica: beta-caryophyllene (JX848729), germacrene-D (JX848730); Nicotiana tabacum aristolochene (AAA19216); Ocimum basilicum: gamma-cadinene (AAV63787), germacrene-D (Q5SBP6), selinene (Q5SBP7), alpha-zingiberene (Q5SBP4); Perilla frutescens: valencene (AAX16077), Pogostemon cablin: germacrene-D (AAS86322); Solidago Canadensis: germacrene-D (AAR31144); Solanum lycopersicum: germacrene-C (AAC39432), vetispiradiene (AAG09949); Solanum tuberosum: vetispiradiene (BAA82108); Vitis vinifera: alpha-bergamotene (ADR74195), alpha-farnesene (ADR74198), alpha-humulene (ADR74227), germacrene-D (AAS66357), valencene (AAS66358); Zea mays: beta-farnesene (Q2NM15).

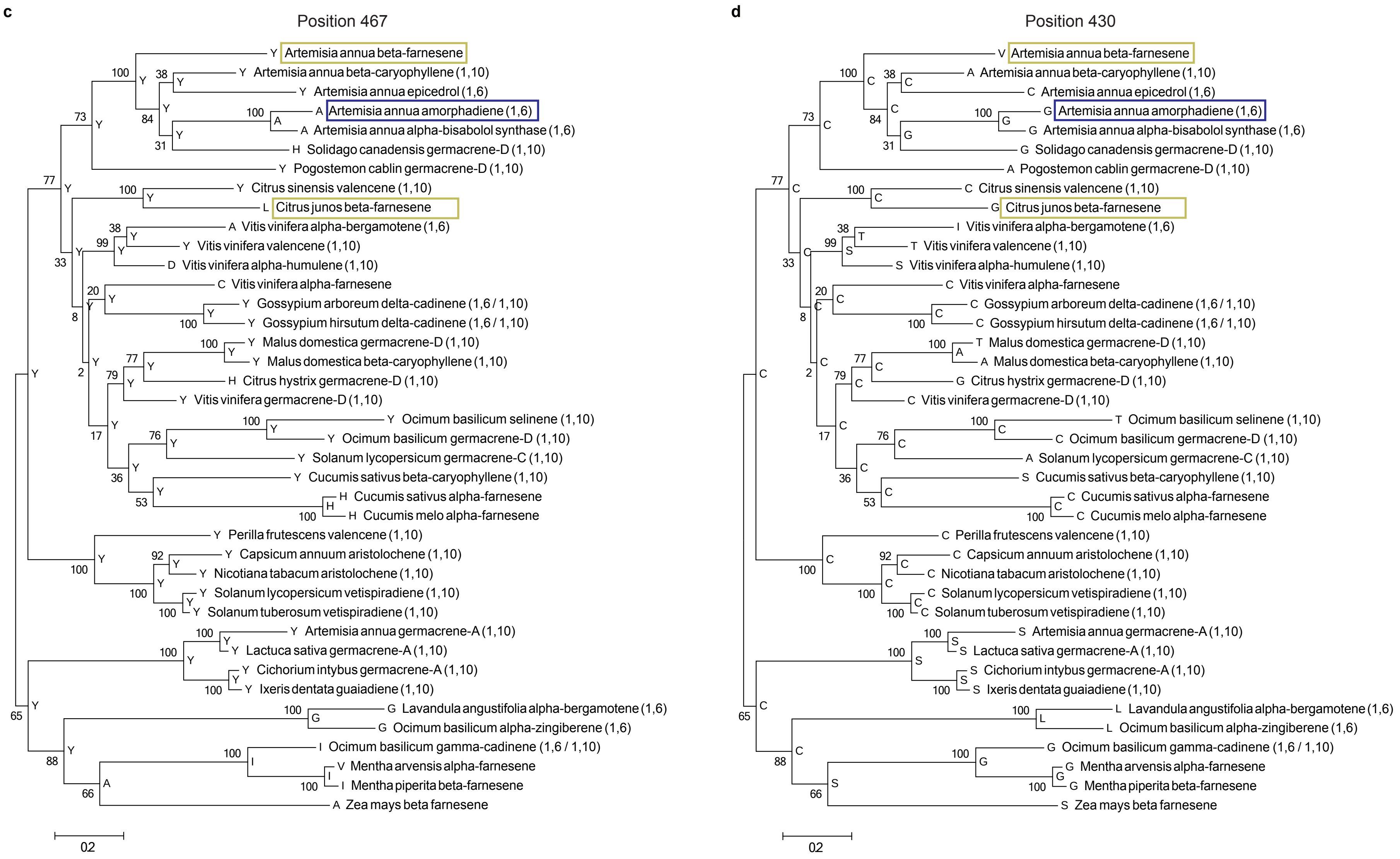

Supplementary Figure 9 continued

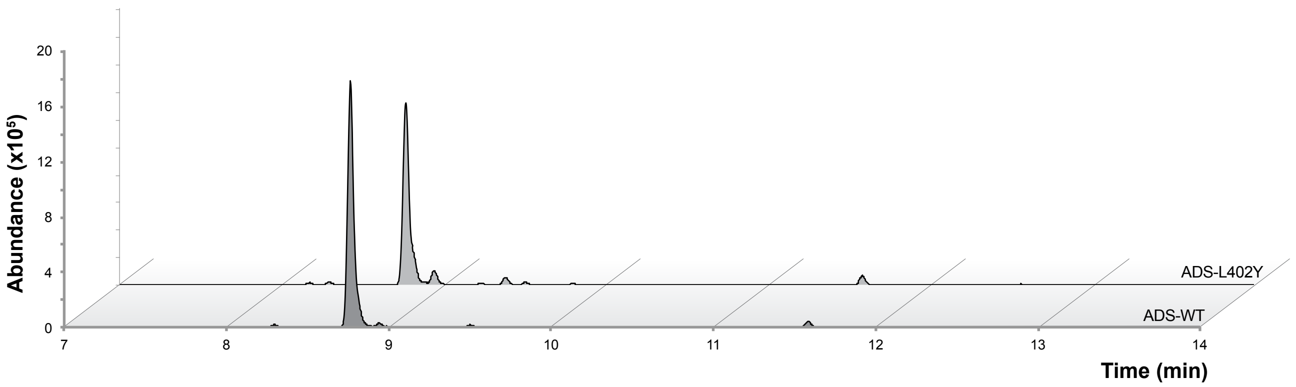

Supplementary Figure 10. Total ion chromatogram for *A. annua* ADS-WT and L402Y mutant
Total ion chromatograms of *A. annua* amorphadiene synthase (ADS) wild-type (WT) and L402Y mutant. Incorporation of L402Y mutation reduced product specificity of ADS from 93.9% to 82.2% (a percentage decrease of 12.5%), and increased minor product diversity from 8 terpene products to 10. Catalytic efficiency of the L402Y mutant is also reduced by 44%.

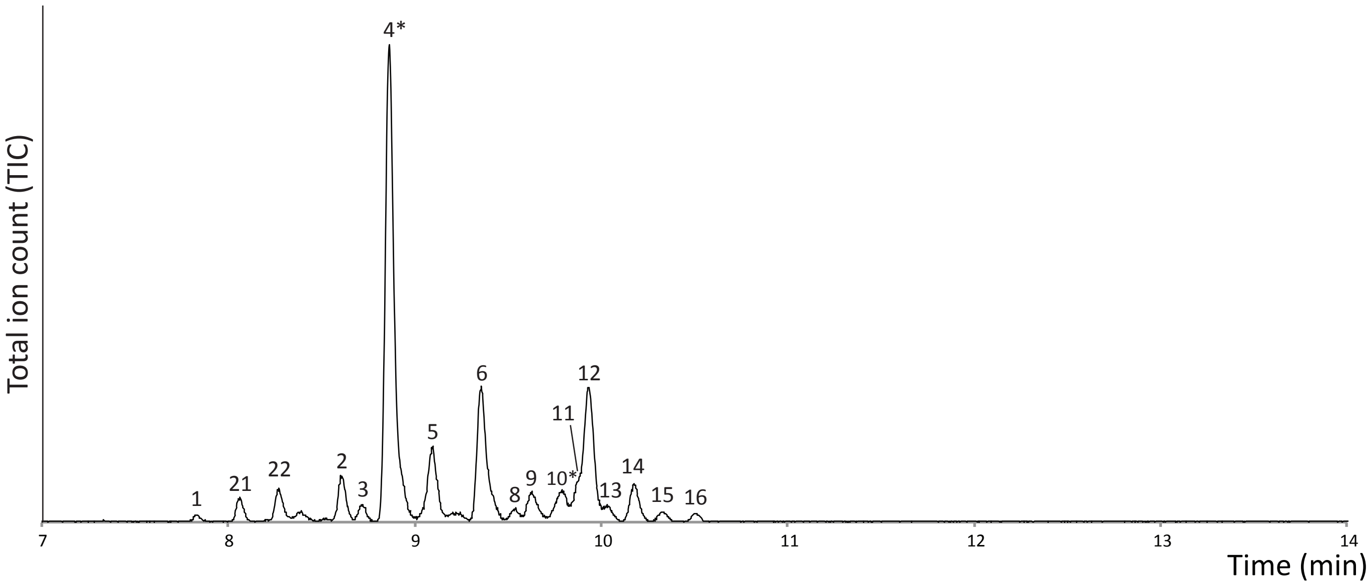

Supplementary Figure 11. Total ion chromatogram for *C. junos* BFS-467 mutant

Total ion chromatogram for the *C. junos* BFS-467 mutant. The G467V mutation is responsible for activating cyclisation in *C. junos* BFS. Labeled peaks were either identified by MS comparisons with authentic standards or matched to the mass spectra of compounds reported in the masssfinder library (Supplementary Figure 5 and Supplementary Table 5). 1, sesquithujene; 2, α-exo-bergamotene; 3, Peak 1; 4, (E)-β-farnesene; 5, putative amorphadiene isomer; 6, γ-curcumene; 7, ar-curcumene; 8, cis-α-bergamotene; 9, zingiberene; 10, α-farnesene; 11, β-bisabolene; 12, β-curcumene; 13, Peak 2; 14, β-sesquiphelladrene; 15, Peak 3; 16, α-bisabolene; 17, nerolidol; 18, Peak 4; 19, α-bisabolol; 20, farnesol; 21, α-cedrene; 22, Unknown 1.

Supplementary Table 1. Oligonucleotide primers used for library cloning, SCOPE and mutagenesis

For *A.annua* BFS mutagenesis, mutations to ADS residues are shown in red, and mutations back to *A.annua* BFS residues are shown in green. For *C.junos* BFS mutagenesis, mutations are shown in blue.

PRIMER NAME	PRIMER SEQUENCE (5'→3')	ZONE NUMBER	PURPOSE
XMA_B1	TCCCCCCGGGGACAAGTTTGTACAAAAAAGC	-	Cloning AaFS N-terminal fragment for 3-plasmid system
291_CLA-	CCGATCGATCATAATTATCAAATGTATCATCTAAAACG	-	Cloning AaFS N-terminal fragment for 3-plasmid system
292_XMA+	TCCCCCCGGGTACTTATGAAGAACTCGAAATCTTTACAC	-	Cloning AaFS Template fragment for 3-plasmid system
435_PST-	GGCCTGCAGAGGAGGATACGAGGACACCCATTTAAAGG	-	Cloning AaFS Template fragment for 3-plasmid system
436_XMA+	CACTGCCCGGGATTATAAAAGCTTCATGTGTGATTGTAAGAC	-	Cloning AaFS C-terminal fragment for 3-plasmid system
BAM_B2	GGCGGATCCGGGGACCACTTTGTACAAGAAAGCTGGG	-	Cloning AaFS C-terminal fragment for 3-plasmid system
F_attB1	CCTGGCTTGCTCGGATAGAAC**ACAAGTTTGTACAAAAAAGCAGGC**	-	Amplification of N-terminal fragment and full length gene (long primer)
NT_xover_FS	GATTTCGAGTTCTTCATAAGTACCATAATTATCAAATGTATCATC	-	Amplification of N-terminal fragment
attB2_6	GGACACACTTAGCCTTCCAGG**ACCACTTTGTACAAGAAAGCTGGG**	-	Amplification of C-terminal fragment and full length gene (long primer)
TC_xover_FS	CCGAGGATACCTTTAAATGGGTGTCCTCGTATCCTCCTATTA	-	Amplification of C-terminal fragment
F+	CCTGGCTTGCTCGGATAGAAC	-	Amplification of full length gene (short primer)
6-	GGACACACTTAGCCTTCCAGG	-	Amplification of full length gene (short primer)
N1.11-F	CGAGACAGGGTGGTGGAATGTTTATTTTTGG	1	Zone 1 mutagenesis
N1.11-R	CCAAAAATAACATTCCACCACCCTGTCTCG	1	Zone 1 mutagenesis
N2.2-F	AGAATGTTCCTAATGAAAACATGCATGTGGTTAGTCGTTTTAGAT	2	Zone 2 mutagenesis
N2.2-R	ATCTAAAACGACTAACCACATGCATGTTTTCATTAGGAACATTCT	2	Zone 2 mutagenesis
N2.3-F	AGAATGTTCCTAATGAAAGCTTGCATGTGGTTAGTCGTTTTAGAT	2	Zone 2 mutagenesis
N2.3-R	ATCTAAAACGACTAACCACATGCAAGCTTTCATTAGGAACATTCT	2	Zone 2 mutagenesis
N2.4-F	AGAATGTTCCTAATGAAAACAGTAATGTGGTTAGTCGTTTTAGAT	2	Zone 2 mutagenesis
N2.4-R	ATCTAAAACGACTAACCACATTACTGTTTTCATTAGGAACATTCT	2	Zone 2 mutagenesis
N2.5-F	AGAATGTTCCTAATGAAAACATGCGCTTGGTTAGTCGTTTTAGAT	2	Zone 2 mutagenesis
N2.5-R	ATCTAAAACGACTAACCAAGCGCATGTTTTCATTAGGAACATTCT	2	Zone 2 mutagenesis
N2.6-F	AGAATGTTCCTAATGAAAACATGCATGGTTTTAGTCGTTTTAGAT	2	Zone 2 mutagenesis
N2.6-R	ATCTAAAACGACTAAAACCATGCATGTTTTCATTAGGAACATTCT	2	Zone 2 mutagenesis
N2.7-F	AGAATGTTCCTAATGAAAGCTGTAATGTGGTTAGTCGTTTTAGAT	2	Zone 2 mutagenesis
N2.7-R	ATCTAAAACGACTAACCACATTACAGCTTTCATTAGGAACATTCT	2	Zone 2 mutagenesis
N2.8-F	AGAATGTTCCTAATGAAAGCTTGCGCTTGGTTAGTCGTTTTAGAT	2	Zone 2 mutagenesis
N2.8-R	ATCTAAAACGACTAACCAAGCGCAAGCTTTCATTAGGAACATTCT	2	Zone 2 mutagenesis
N2.9-F	AGAATGTTCCTAATGAAAGCTTGCATGGTTTTAGTCGTTTTAGAT	2	Zone 2 mutagenesis
N2.9-R	ATCTAAAACGACTAAAACCATGCAAGCTTTCATTAGGAACATTCT	2	Zone 2 mutagenesis
N2.10-F	AGAATGTTCCTAATGAAAACAGTAGCTTGGTTAGTCGTTTTAGAT	2	Zone 2 mutagenesis
N2.10-R	ATCTAAAACGACTAACCAAGCTACTGTTTTCATTAGGAACATTCT	2	Zone 2 mutagenesis
N2.11-F	AGAATGTTCCTAATGAAAACAGTAATGGTTTTAGTCGTTTTAGAT	2	Zone 2 mutagenesis
N2.11-R	ATCTAAAACGACTAAAACCATTACTGTTTTCATTAGGAACATTCT	2	Zone 2 mutagenesis
N2.12-F	AGAATGTTCCTAATGAAAACATGCGCTGTTTTAGTCGTTTTAGAT	2	Zone 2 mutagenesis
N2.12-R	ATCTAAAACGACTAAAACAGCGCATGTTTTCATTAGGAACATTCT	2	Zone 2 mutagenesis
N2.13-F	AGAATGTTCCTAATGAAAGCTGTAGCTTGGTTAGTCGTTTTAGAT	2	Zone 2 mutagenesis
N2.13-R	ATCTAAAACGACTAACCAAGCTACAGCTTTCATTAGGAACATTCT	2	Zone 2 mutagenesis
N2.14-F	AGAATGTTCCTAATGAAAGCTGTAATGGTTTTAGTCGTTTTAGAT	2	Zone 2 mutagenesis
N2.14-R	ATCTAAAACGACTAAAACCATTACAGCTTTCATTAGGAACATTCT	2	Zone 2 mutagenesis
N2.15-F	AGAATGTTCCTAATGAAAGCTTGCGCTGTTTTAGTCGTTTTAGAT	2	Zone 2 mutagenesis
N2.15-R	ATCTAAAACGACTAAAACAGCGCAAGCTTTCATTAGGAACATTCT	2	Zone 2 mutagenesis
N2.16-F	AGAATGTTCCTAATGAAAACAGTAGCTGTTTTAGTCGTTTTAGAT	2	Zone 2 mutagenesis
N2.16-R	ATCTAAAACGACTAAAACAGCTACTGTTTTCATTAGGAACATTCT	2	Zone 2 mutagenesis
N2.17-F	AGAATGTTCCTAATGAAAGCTGTAGCTGTTTTAGTCGTTTTAGAT	2	Zone 2 mutagenesis
N2.17-R	ATCTAAAACGACTAAAACAGCTACAGCTTTCATTAGGAACATTCT	2	Zone 2 mutagenesis
N2.18-F	ATGAAAACATGCATGTGGTTAGTCGTTTTAGATGATACATTTGAT	2	Zone 2 mutagenesis
N2.18-R	ATCAAATGTATCATCTAAAACGACTAACCACATGCATGTTTTCAT	2	Zone 2 mutagenesis
N2.19-F	ATGAAAACATGCATGTGGATCGTCGTTTTAGATGATACATTTGAT	2	Zone 2 mutagenesis
N2.19-R	ATCAAATGTATCATCTAAAACGACGATCCACATGCATGTTTTCAT	2	Zone 2 mutagenesis
N2.20-F	ATGAAAACATGCATGTGGTTAACCGTTTTAGATGATACATTTGAT	2	Zone 2 mutagenesis
N2.20-R	ATCAAATGTATCATCTAAAACGGTTAACCACATGCATGTTTTCAT	2	Zone 2 mutagenesis
N2.21-F	ATGAAAACATGCATGTGGTTAGTCCTGTTAGATGATACATTTGAT	2	Zone 2 mutagenesis
N2.21-R	ATCAAATGTATCATCTAACAGGACTAACCACATGCATGTTTTCAT	2	Zone 2 mutagenesis
N2.22-F	ATGAAAACATGCATGTGGATCACCGTTTTAGATGATACATTTGAT	2	Zone 2 mutagenesis
N2.22-R	ATCAAATGTATCATCTAAAACGGTGATCCACATGCATGTTTTCAT	2	Zone 2 mutagenesis
N2.23-F	ATGAAAACATGCATGTGGATCGTCCTGTTAGATGATACATTTGAT	2	Zone 2 mutagenesis
N2.23-R	ATCAAATGTATCATCTAACAGGACGATCCACATGCATGTTTTCAT	2	Zone 2 mutagenesis
N2.24-F	ATGAAAACATGCATGTGGTTAACCCTGTTAGATGATACATTTGAT	2	Zone 2 mutagenesis
N2.24-R	ATCAAATGTATCATCTAACAGGGTTAACCACATGCATGTTTTCAT	2	Zone 2 mutagenesis
N2.25-F	ATGAAAACATGCATGTGGATCACCCTGTTAGATGATACATTTGAT	2	Zone 2 mutagenesis
N2.25-R	ATCAAATGTATCATCTAACAGGGTGATCCACATGCATGTTTTCAT	2	Zone 2 mutagenesis
T3.1-F	GAGATGGCAAAAGAGTTGGTTCGCAATTACTTGGTAGAAGCCAGA	3	Zone 3 mutagenesis
T3.1-R	TCTGGCTTCTACCAAGTAATTGCGAACCAACTCTTTTGCCATCTC	3	Zone 3 mutagenesis
T3.2-F	GAGATGGCAAAAGAGTTCGTTCGCAATTACTTGGTAGAAGCCAGA	3	Zone 3 mutagenesis
T3.2-R	TCTGGCTTCTACCAAGTAATTGCGAACGAACTCTTTTGCCATCTC	3	Zone 3 mutagenesis
T3.3-F	GAGATGGCAAAAGAGTTGGTTCGCAATCTGTTGGTAGAAGCCAGA	3	Zone 3 mutagenesis
T3.3-R	TCTGGCTTCTACCAACAGATTGCGAACCAACTCTTTTGCCATCTC	3	Zone 3 mutagenesis
T3.4-F	GAGATGGCAAAAGAGTTCGTTCGCAATCTGTTGGTAGAAGCCAGA	3	Zone 3 mutagenesis
T3.4-R	TCTGGCTTCTACCAACAGATTGCGAACGAACTCTTTTGCCATCTC	3	Zone 3 mutagenesis
T4.1-F	TCAATGGTGACTGGTACCTATGGCTTGATGATAGCGAGATCTTAT	4	Zone 4 mutagenesis
T4.1-R	ATAAGATCTCGCTATCATCAAGCCATAGGTACCAGTCACCATTGA	4	Zone 4 mutagenesis
T4.2-F	TCAATGGTGACTGGTGGTTATGGCTTGATGATAGCGAGATCTTAT	4	Zone 4 mutagenesis
T4.2-R	ATAAGATCTCGCTATCATCAAGCCATAACCACCAGTCACCATTGA	4	Zone 4 mutagenesis
T4.3-F	TCAATGGTGACTGGTACCGCAGGCTTGATGATAGCGAGATCTTAT	4	Zone 4 mutagenesis
T4.3-R	ATAAGATCTCGCTATCATCAAGCCTGCGGTACCAGTCACCATTGA	4	Zone 4 mutagenesis
T4.4-F	TCAATGGTGACTGGTACCTATGGCTTGCTGATAGCGAGATCTTAT	4	Zone 4 mutagenesis
T4.4-R	ATAAGATCTCGCTATCAGCAAGCCATAGGTACCAGTCACCATTGA	4	Zone 4 mutagenesis
T4.5-F	TCAATGGTGACTGGTGGTGCAGGCTTGATGATAGCGAGATCTTAT	4	Zone 4 mutagenesis
T4.5-R	ATAAGATCTCGCTATCATCAAGCCTGCACCACCAGTCACCATTGA	4	Zone 4 mutagenesis
T4.6-F	TCAATGGTGACTGGTGGTTATGGCTTGCTGATAGCGAGATCTTAT	4	Zone 4 mutagenesis
T4.6-R	ATAAGATCTCGCTATCAGCAAGCCATAACCACCAGTCACCATTGA	4	Zone 4 mutagenesis

Supplementary Table 1 continued

Primer name	PRIMER SEQUENCE	Zone number	Purpose
T4.7-F	TCAATGGTGACTGGTACCGCAGGCTTGCTGATAGCGAGATCTTAT	4	Zone 4 mutagenesis
T4.7-R	ATAAGATCTCGCTATCAGCAAGCCTGCGGTACCAGTCACCATTGA	4	Zone 4 mutagenesis
T4.8-F	TCAATGGTGACTGGTGGTGCAGGCTTGCTGATAGCGAGATCTTAT	4	Zone 4 mutagenesis
T4.8-R	ATAAGATCTCGCTATCAGCAAGCCTGCACCACCAGTCACCATTGA	4	Zone 4 mutagenesis
C5.2-F	GCTTCATGTGTGATTGTAAGACTTATGGATGATATTGTCAGCCAC	5	Zone 5 mutagenesis
C5.2-R	GTGGCTGACAATATCATCCATAAGTCTTACAATCACACATGAAGC	5	Zone 5 mutagenesis
C5.3-F	GCTTCATGTGTGATTGGTAGACTTATGGATGATATTGTCAGCCAC	5	Zone 5 mutagenesis
C5.3-R	GTGGCTGACAATATCATCCATAAGTCTACCAATCACACATGAAGC	5	Zone 5 mutagenesis
C5.4-F	GCTTCATGTGTGATTGTAAGACTTCTGGATGATATTGTCAGCCAC	5	Zone 5 mutagenesis
C5.4-R	GTGGCTGACAATATCATCCAGAAGTCTTACAATCACACATGAAGC	5	Zone 5 mutagenesis
C5.5-F	GCTTCATGTGTGATTGTAAGACTTATGAACGATATTGTCAGCCAC	5	Zone 5 mutagenesis
C5.5-R	GTGGCTGACAATATCGTTCATAAGTCTTACAATCACACATGAAGC	5	Zone 5 mutagenesis
C5.6-F	GCTTCATGTGTGATTGGTAGACTTCTGGATGATATTGTCAGCCAC	5	Zone 5 mutagenesis
C5.6-R	GTGGCTGACAATATCATCCAGAAGTCTACCAATCACACATGAAGC	5	Zone 5 mutagenesis
C5.7-F	GCTTCATGTGTGATTGGTAGACTTATGAACGATATTGTCAGCCAC	5	Zone 5 mutagenesis
C5.7-R	GTGGCTGACAATATCGTTCATAAGTCTACCAATCACACATGAAGC	5	Zone 5 mutagenesis
C5.8-F	GCTTCATGTGTGATTGTAAGACTTCTGAACGATATTGTCAGCCAC	5	Zone 5 mutagenesis
C5.8-R	GTGGCTGACAATATCGTTCAGAAGTCTTACAATCACACATGAAGC	5	Zone 5 mutagenesis
C5.9-F	GCTTCATGTGTGATTGGTAGACTTCTGAACGATATTGTCAGCCAC	5	Zone 5 mutagenesis
C5.9-R	GTGGCTGACAATATCGTTCAGAAGTCTACCAATCACACATGAAGC	5	Zone 5 mutagenesis
C5.10-F	ATTGTAAGACTTATGGATGATATTGTCAGCCACAAGGAGGAACAA	5	Zone 5 mutagenesis
C5.10-R	TTGTTCCTCCTTGTGGCTGACAATATCATCCATAAGTCTTACAAT	5	Zone 5 mutagenesis
C5.11-F	ATTGTAAGACTTATGGATGATATTATGAGCCACAAGGAGGAACAA	5	Zone 5 mutagenesis
C5.11-R	TTGTTCCTCCTTGTGGCTCATAATATCATCCATAAGTCTTACAAT	5	Zone 5 mutagenesis
C5.12-F	ATTGTAAGACTTATGGATGATATTGTCACCCACAAGGAGGAACAA	5	Zone 5 mutagenesis
C5.12-R	TTGTTCCTCCTTGTGGGTGACAATATCATCCATAAGTCTTACAAT	5	Zone 5 mutagenesis
C5.13-F	ATTGTAAGACTTATGGATGATATTATGACCCACAAGGAGGAACAA	5	Zone 5 mutagenesis
C5.13-R	TTGTTCCTCCTTGTGGGTCATAATATCATCCATAAGTCTTACAAT	5	Zone 5 mutagenesis
C6.2-F	ATCAACCTTGCACGTATGTGTGAAGTCCTATATAGCGTCAACGAT	6	Zone 6 mutagenesis
C6.2-R	ATCGTTGACGCTATATAGGACTTCACACATACGTGCAAGGTTGAT	6	Zone 6 mutagenesis
C6.3-F	ATCAACCTTGCACGTTTCTGTGAAGTCCTATATAGCGTCAACGAT	6	Zone 6 mutagenesis
C6.3-R	ATCGTTGACGCTATATAGGACTTCACAGAAACGTGCAAGGTTGAT	6	Zone 6 mutagenesis
C6.4-F	ATCAACCTTGCACGTATGCTGGAAGTCCTATATAGCGTCAACGAT	6	Zone 6 mutagenesis
C6.4-R	ATCGTTGACGCTATATAGGACTTCCAGCATACGTGCAAGGTTGAT	6	Zone 6 mutagenesis
C6.5-F	ATCAACCTTGCACGTATGTGTGAAGTCCAGTATAGCGTCAACGAT	6	Zone 6 mutagenesis
C6.5-R	ATCGTTGACGCTATACTGGACTTCACACATACGTGCAAGGTTGAT	6	Zone 6 mutagenesis
C6.6-F	ATCAACCTTGCACGTTTCCTGGAAGTCCTATATAGCGTCAACGAT	6	Zone 6 mutagenesis
C6.6-R	ATCGTTGACGCTATATAGGACTTCCAGGAAACGTGCAAGGTTGAT	6	Zone 6 mutagenesis
C6.7-F	ATCAACCTTGCACGTTTCTGTGAAGTCCAGTATAGCGTCAACGAT	6	Zone 6 mutagenesis
C6.7-R	ATCGTTGACGCTATACTGGACTTCACAGAAACGTGCAAGGTTGAT	6	Zone 6 mutagenesis
C6.8-F	ATCAACCTTGCACGTATGCTGGAAGTCCAGTATAGCGTCAACGAT	6	Zone 6 mutagenesis
C6.8-R	ATCGTTGACGCTATACTGGACTTCCAGCATACGTGCAAGGTTGAT	6	Zone 6 mutagenesis
C6.9-F	ATCAACCTTGCACGTTTCCTGGAAGTCCAGTATAGCGTCAACGAT	6	Zone 6 mutagenesis
C6.9-R	ATCGTTGACGCTATACTGGACTTCCAGGAAACGTGCAAGGTTGAT	6	Zone 6 mutagenesis
C6.11-F	AACGATGGCTTCACTCATGCTGAGGGAGACATGAAAAGTTACATG	6	Zone 6 mutagenesis
C6.11-R	CATGTAACTTTTCATGTCTCCCTCAGCATGAGTGAAGCCATCGTT	6	Zone 6 mutagenesis
C6.12-F	AACGATGGCTTCACTCGCGCTGAGGGAGACATGAAAAGTTACATG	6	Zone 6 mutagenesis
C6.12-R	CATGTAACTTTTCATGTCTCCCTCAGCGCGAGTGAAGCCATCGTT	6	Zone 6 mutagenesis
C6.13-F	AACGATGGCTTCACTCATGCTGGCGGAGACATGAAAAGTTACATG	6	Zone 6 mutagenesis
C6.13-R	CATGTAACTTTTCATGTCTCCGCCAGCATGAGTGAAGCCATCGTT	6	Zone 6 mutagenesis
C6.14-F	AACGATGGCTTCACTCATGCTGAGGGAGAAATGAAAAGTTACATG	6	Zone 6 mutagenesis
C6.14-R	CATGTAACTTTTCATTTCTCCCTCAGCATGAGTGAAGCCATCGTT	6	Zone 6 mutagenesis
C6.15-F	AACGATGGCTTCACTCGCGCTGGCGGAGACATGAAAAGTTACATG	6	Zone 6 mutagenesis
C6.15-R	CATGTAACTTTTCATGTCTCCGCCAGCGCGAGTGAAGCCATCGTT	6	Zone 6 mutagenesis
C6.16-F	AACGATGGCTTCACTCGCGCTGAGGGAGAAATGAAAAGTTACATG	6	Zone 6 mutagenesis
C6.16-R	CATGTAACTTTTCATTTCTCCCTCAGCGCGAGTGAAGCCATCGTT	6	Zone 6 mutagenesis
C6.17-F	AACGATGGCTTCACTCATGCTGGCGGAGAAATGAAAAGTTACATG	6	Zone 6 mutagenesis
C6.17-R	CATGTAACTTTTCATTTCTCCGCCAGCATGAGTGAAGCCATCGTT	6	Zone 6 mutagenesis
C6.18-F	AACGATGGCTTCACTCGCGCTGGCGGAGAAATGAAAAGTTACATG	6	Zone 6 mutagenesis
C6.18-R	CATGTAACTTTTCATTTCTCCGCCAGCGCGAGTGAAGCCATCGTT	6	Zone 6 mutagenesis
G296C-F	GGTGGAATGTTATTTTTGGATACTATCCATCTATTACGAGC	1	AaFS deconvolution mutagenesis - Introduce G296C mutation
G296C-R	CAAAAATAACATTCCACCACCCTGTCTCGAACATAAG	1	AaFS deconvolution mutagenesis - Introduce G296C mutation
Y402L-F	CGCAATCTGTTGGTAGAAGCCAGATGGCTAAAAG	3	AaFS deconvolution mutagenesis - Introduce Y402L mutation
Y402L-R	CTACCAACAGATTGCGAACCAACTCTTTTGCCATC	3	AaFS deconvolution mutagenesis - Introduce Y402L mutation
Y402FL-R	CTACCAACAGATTGCGAACGAACTCTTTTGCCATC	3	AaFS deconvolution mutagenesis - Introduce Y402L/L398F mutations
402WT-F	CGCAATTACTTGGTAGAAGCCAGATGGCTAAAAG	3	AaFS deconvolution mutagenesis - Introduce WT L402Y mutation
402WT-R	CTACCAAGTAATTGCGAACCAACTCTTTTGCCATC	3	AaFS deconvolution mutagenesis - Introduce WT L402Y/F398L mutations
402WT+F-R	CTACCAAGTAATTGCGAACGAACTCTTTTGCCATC	3	AaFS deconvolution mutagenesis - Introduce WT L402Y and L398F mutations
D471N-F	GACTTATGAACGATATTGTCAGCCACAAGGAGGAAC	5	AaFS deconvolution mutagenesis - Introduce D471N mutation
V467GN-R	ACAATATCGTTCATAAGTCTACCAATCACACATGAAGC	5	AaFS deconvolution mutagenesis - Introduce V467G/D471N mutations
467WT-F	GACTTATGGATGATATTGTCAGCCACAAGGAGGAAC	5	AaFS deconvolution mutagenesis - Introduce WT N471D mutation
467WT-R	ACAATATCATCCATAAGTCTTACAATCACACATGAAGC	5	AaFS deconvolution mutagenesis - Introduce WT G467V/N471D mutation
V467G-R	ACAATATCATCCATAAGTCTACCAATCACACATGAAGC	5	AaFS deconvolution mutagenesis - Introduce V467G mutation
D471N-R	ACAATATCGTTCATAAGTCTTACAATCACACATGAAGC	5	AaFS deconvolution mutagenesis - Introduce D471N mutation
T429G-F	CTGGTGGTTATGGCTTGATGATAGCGAGATCTTATG	4	AaFS deconvolution mutagenesis - Introduce T429G mutation
T429G-R	GCCATAACCACCAGTCACCATTGACACAGACATG	4	AaFS deconvolution mutagenesis - Introduce T429G mutation
T429GL-F	CTGGTGGTTATGGCTTGCTGATAGCGAGATCTTATG	4	AaFS deconvolution mutagenesis - Introduce T429G/M433L mutations
T419GAL-F	GGTGGTGCAGGCTTGCTGATAGCGAGATCTTATG	4	AaFS deconvolution mutagenesis - Introduce T429GY430A/M433L mutations
T429GAL-R	GCCTGCACCACCAGTCACCATTGACACAGAC	4	AaFS deconvolution mutagenesis - Introduce T429G/Y430A mutations
Y430WT-F	CTGGTACCTATGGCTTGATGATAGCGAGATCTTATG	4	AaFS deconvolution mutagenesis - Introduce WT A430Y mutation
Y430WT-R	GCCATAGGTACCAGTCACCATTGACACAGACATG	4	AaFS deconvolution mutagenesis - Introduce WT A430Y mutation
Y430A-F	GGTACCGCAGGCTTGATGATAGCGAGATCTTATG	4	AaFS deconvolution mutagenesis - Introduce Y430A mutation
Y430A-R	GCCTGCGGTACCAGTCACCATTGACACAGAC	4	AaFS deconvolution mutagenesis - Introduce Y430A mutation

Supplementary Table 1 continued

Primer name	PRIMER SEQUENCE	Zone number	Purpose
Y430GA-F	GGTGGTGCAGGCTTGATGATAGCGAGATCTTATG	4	AaFS deconvolution mutagenesis - Introduce T429G/Y430A mutation
Y430GA-R	GCCTGCACCACCAGTCACCATTGACACAGAC	4	AaFS deconvolution mutagenesis - Introduce T429G/Y430A mutation
M470LN-F	GACTTCTGAACGATATTGTCAGCCACAAGGAGGAAC	5	AaFS deconvolution mutagenesis - Introduce M470L/D471N mutations
V467GLN-R	ACAATATCGTTCAGAAGTCTACCAATCACACATGAAGCTTTT	5	AaFS deconvolution mutagenesis - Introduce V467G/M470L/D471N mutations
S475T-F	GACTTATGGATGATATTGTCACCCACAAGGAGGAAC	5	AaFS deconvolution mutagenesis - Introduce S475T mutation
M470L-F	GACTTCTGGATGATATTGTCAGCCACAAGGAGGAAC	5	AaFS deconvolution mutagenesis - Introduce M470L mutation
V467GL-R	ACAATATCATCCAGAAGTCTACCAATCACACATGAAGCTTTT	5	AaFS deconvolution mutagenesis - Introduce V467G/M470L mutations
CjFS-402-F	TTATATTGCTGTTTACCGAAGCCAAGTGGTTATATAAAG	3	CjFS mutagenesis - Introduce Y402L mutation
CjFS-402-R	CGGTAAACAGCAATATAATTAATTCTTGCATCATCTGTTTTG	3	CjFS mutagenesis - Introduce Y402L mutation
CjFS-467-F	CCATTGTAAGGCTCATGGATGACATAGCAGG	5	CjFS mutagenesis - Introduce G467V mutation
CjFS-467-R	GAGCCTTACAATGGTTTCTGTCGCTTTAAGAC	5	CjFS mutagenesis - Introduce G467V mutation
CjFS-430-F	TCGGTGCACGTACGCTAGCGGTCGCTTCC	4	CjFS mutagenesis - Introduce L430A mutation
CjFS-430-R	CGTACGTGCACCGATACTTCTTAATGCAACTGATTTG	4	CjFS mutagenesis - Introduce L430A mutation

Supplementary Table 2. Residue name and codon sequence for the 24 amino acids targeted for mutagenesis

A. annua BFS amino acid number	*A. annua* BFS		*A. annua* ADS		*C. junos* BFS	
	Residue	Codon	Residue	Codon	Residue	Codon
296	G	ggc	C	tgt		
319	T	aca	A	gct		
320	C	tgc	V	gta		
321	M	atg	A	gct		
322	W	tgg	V	gtt		
323	L	tta	I	atc		
324	V	gtc	T	acc		
325	V	gtt	L	ctg		
398	L	ttg	F	ttc		
402	Y	tac	L	ctg	Y	tac
429	T	acc	G	ggt		
430	Y	tat	A	gca	L	tta
433	M	atg	L	ctg		
467	V	gta	G	ggt	G	ggc
470	M	atg	L	ctg		
471	D	gat	N	aac		
474	V	gtc	M	atg		
475	S	agc	T	acc		
542	M	atg	F	ttc		
543	C	tgt	L	ctg		
546	L	cta	Q	cag		
555	H	cat	R	cgc		
557	E	gag	G	ggc		
559	D	gac	E	gaa		

Supplementary Table 3. Sequence and kinetic analysis of the *A. annua* BFS 6Å library

Mutant name	Number of mutations	Kinetic parameters: Protein concentration (µM)	kcat apparent (min^{-1})	% of AaFS-WT activity	Product analysis: Linear products (%)	Cyclic products (%)	Background 402	467	430
**AaFS 6Å library**									
1.03-402	3	2.38	0.60	2.02	93	7	Y	V	Y
1.03+402	4	3.28	0.98	3.29	69	31	L	V	Y
1.03+467	6	2.70	0.30	1.01	86	14	L	G	Y
1.12+402	6	2.88	1.64	4.80	45	55	L	V	Y
1.16-402	4	2.40	1.19	3.48	98	2	Y	V	Y
1.16+402	5	2.54	1.72	5.02	60	40	L	V	Y
1.16+467	6	3.19	0.35	1.01	87	13	L	G	Y
1.25-402	5	3.41	2.70	6.21	95	5	Y	G	Y
1.25+402	5	3.52	10.93	25.18	34	66	L	V	Y
1.25+467	6	3.42	1.03	2.37	89	11	L	G	Y
1.34-402	4	2.61	0.88	2.47	94	6	Y	V	Y
1.34+402	5	1.97	1.27	4.08	29	71	L	V	Y
1.34+467	6	2.69	0.29	0.97	78	22	L	G	Y
1.37-402	5	1.95	1.56	5.00	95	5	Y	G	A
1.37+430	6	2.80	1.07	3.69	63	37	L	G	A
1.37+430	6	2.95	1.89	6.06	67	33	L	G	A
1.37+467	5	2.67	1.23	3.92	91	9	L	G	Y
2.05+402	3	2.73	0.67	2.25	60	40	L	V	A
2.09-2-402	4	2.06	1.03	3.00	98	2	Y	V	A
2.09-402	3	3.87	1.74	4.00	98	2	Y	V	Y
2.09+402	4	3.65	1.87	5.99	31	69	L	V	Y
2.09+467	5	3.47	3.41	7.86	95	5	L	G	Y
2.12+402	6	2.45	0.99	2.90	34	66	L	V	A
2.22+402	4	2.75	2.94	9.86	32	68	L	V	Y
2.22+467	5	2.84	0.78	2.62	81	19	L	G	Y
2.28-402	4	2.90	0.72	2.36	98	2	Y	G	Y
2.28+402	3	3.18	0.57	1.85	66	34	L	V	Y
2.28+430	6	3.19	1.30	4.26	69	31	L	G	A
2.28+467	5	2.80	0.91	2.98	95	5	L	G	Y
2.37-402	3	3.01	2.47	5.69	97	3	Y	G	Y
2.37+402	3	3.04	1.67	3.84	38	62	L	V	Y
2.37+430	5	2.09	1.50	4.91	72	28	L	G	A
2.37+467	4	3.41	1.50	3.46	94	6	L	G	Y
2.39-402	3	2.46	7.61	21.49	98	2	Y	V	A
2.39+402	4	2.60	8.32	23.49	13	87	L	V	A
2.39+430	5	2.83	1.94	6.26	65	35	L	G	A
2.41-402	4	2.07	3.04	8.57	97	3	Y	V	Y
2.41+296	6	2.29	0.39	1.14	36	64	L	V	Y
2.41+402	5	2.20	6.22	17.55	56	44	L	V	Y
2.46+402	4	2.97	0.64	2.09	67	33	L	V	Y
2.46+430	7	2.49	0.80	2.62	63	37	L	G	A
2.46+467	6	2.21	0.54	1.77	90	10	L	G	Y
2.61-402	4	2.93	2.74	7.72	92	8	Y	V	Y
2.61+402	5	2.30	8.69	24.53	25	75	L	V	Y
2.61+467	6	3.64	0.82	2.65	84	16	L	G	Y
3.03-402	5	2.52	1.98	6.38	98	2	Y	V	A
3.03+402	6	2.52	1.03	3.30	28	72	L	V	A
3.05+402	6	2.20	0.85	2.93	62	38	L	V	A
3.05+430	7	2.66	1.27	4.38	41	59	L	G	A
3.10-402	5	2.12	1.54	3.54	95	5	Y	G	Y
3.10+402	4	3.48	4.44	10.23	23	77	L	V	Y
3.10+430	7	3.03	1.86	6.00	61	39	L	G	A
3.10+467	6	3.14	0.32	0.75	87	13	L	G	Y
3.26-402	4	2.15	1.07	3.43	95	5	Y	V	A
3.26+402	5	2.48	1.70	3.92	23	77	L	V	A
3.26+467	6	2.78	0.85	2.72	76	24	L	G	A
3.29-402	3	3.08	2.00	6.45	94	6	Y	V	Y
3.29+402	4	2.73	4.72	15.22	20	80	L	V	Y
3.36+402	4	2.59	0.91	2.92	59	41	L	V	A
3.36+430	6	2.03	3.19	10.72	55	45	L	G	A
3.36+467	5	2.36	1.66	5.34	89	11	L	G	Y
4.02+467	6	2.48	1.43	4.61	93	7	L	G	Y
4.12-402	3	2.77	3.03	9.77	97	3	Y	V	A
4.12+402	4	2.85	0.84	2.71	26	74	L	V	A
5.01+402	5	2.47	0.65	2.18	40	60	L	V	Y
5.24+402	5	2.46	0.44	1.50	44	56	L	V	Y
5.24+467	6	2.68	0.44	1.52	78	22	L	G	Y
7.02o-402	3	2.75	3.28	9.25	98	2	Y	V	Y
7.02o+402	4	2.37	1.52	4.28	56	44	L	V	Y
7.02o+467	5	3.33	0.73	2.36	89	11	L	G	Y
7.09-402	4	2.08	0.83	2.84	94	6	Y	V	Y
7.09+402	5	2.67	0.00	0.00	77	23	L	V	Y
7.09+467	6	2.33	0.00	0.00	81	19	L	G	Y
7.10-402	6	2.51	1.84	5.94	98	2	Y	V	Y
7.10+402	7	2.76	2.88	9.29	53	47	L	V	Y
7.10+467	8	2.57	2.05	6.62	98	2	L	G	Y
7.11-402	4	2.05	1.02	3.52	96	4	Y	V	A
7.11+402	5	2.32	0.59	2.03	42	58	L	V	A
7.27-402	5	2.51	0.77	2.52	96	4	Y	G	Y
7.27+430	7	2.87	0.54	1.77	75	25	L	G	A
7.27+467	6	3.23	0.49	1.60	94	6	L	G	Y
7.27o-402	3	2.45	13.21	37.30	98	2	Y	V	Y
7.27o+402	4	2.15	3.43	9.67	46	54	L	V	Y

Mutant name		Zone 1				Zone 2											Zone 3										Zone 4								Zone 5												Zone 6														Total number of mutations			
	Residue	294	295	296	297	316	317	318	319	320	321	322	323	324	325	326	395	396	397	398	399	400	401	402	403	404	428	429	430	431	432	433	434	435	465	466	467	468	469	470	471	472	473	474	475	476	541	542	543	544	545	546	547	554	555	556	557	558	559	560				
	AaFS	V	E	G	Y	L	M	K	T	C	M	W	L	V	V	L	A	K	E	L	V	R	N	Y	L	V	G	T	Y	G	L	M	I	A	V	I	V	R	L	M	D	D	I	V	S	H	R	M	C	E	V	L	Y	T	H	A	E	G	D	M				
	AaADS	V	E	C	Y	F	T	K	A	V	A	V	I	T	L	I	V	K	E	F	V	R	N	L	M	V	G	G	A	N	L	L	T	T	I	L	G	R	R	L	N	D	L	M	T	H	Q	F	L	E	V	Q	Y	T	R	M	G	D	E	Y	N	T	C	Total
**AaFS 6Å library**																																																																
1.03-402		V	E	G	Y	L	M	K	T	C	M	W	L	V	V	L	A	K	E	F	V	R	N	Y	L	V	G	T	Y	G	L	M	I	A	V	I	V	R	L	M	D	D	I	V	S	H	R	M	C	E	V	L	Y	T	R	A	E	G	E	M	0	1	2	3
1.03+402		V	E	G	Y	L	M	K	T	C	M	W	L	V	V	L	A	K	E	F	V	R	N	L	L	V	G	T	Y	G	L	M	I	A	V	I	V	R	L	M	D	D	I	V	S	H	R	M	C	E	V	L	Y	T	R	A	E	G	E	M	0	2	2	4
1.03+467		V	E	G	Y	L	M	K	T	C	M	W	L	V	V	L	A	K	E	F	V	R	N	L	L	V	G	G	Y	G	L	M	I	A	V	I	G	R	L	M	D	D	I	V	S	H	R	M	C	E	V	L	Y	T	R	A	E	G	E	M	0	3	3	6
1.12+402		V	E	G	Y	L	M	K	T	C	M	W	L	T	L	L	A	K	E	F	V	R	N	L	L	V	G	G	Y	G	L	M	I	A	V	I	V	R	L	M	N	D	I	V	S	H	R	M	C	E	V	L	Y	T	H	A	E	G	D	M	2	3	1	6
1.16-402		V	E	G	Y	L	M	K	T	C	M	W	L	V	L	L	A	K	E	L	V	R	N	Y	L	V	G	T	Y	G	L	L	I	A	V	I	V	R	L	L	N	D	I	V	S	H	R	M	C	E	V	L	Y	T	H	A	E	G	D	M	1	1	2	4
1.16+402		V	E	G	Y	L	M	K	T	C	M	W	L	V	L	L	A	K	E	L	V	R	N	L	L	V	G	T	Y	G	L	L	I	A	V	I	V	R	L	L	N	D	I	V	S	H	R	M	C	E	V	L	Y	T	H	A	E	G	D	M	1	2	2	5
1.16+467		V	E	G	Y	L	M	K	T	C	M	W	L	V	L	L	A	K	E	L	V	R	N	L	L	V	G	T	Y	G	L	L	I	A	V	I	G	R	L	L	N	D	I	V	S	H	R	M	C	E	V	L	Y	T	H	A	E	G	D	M	1	2	3	6
1.25-402		V	E	G	Y	L	M	K	T	C	M	W	L	T	V	L	A	K	E	L	V	R	N	Y	L	V	G	T	Y	G	L	M	I	A	V	I	G	R	L	M	N	D	I	V	S	H	R	F	C	E	V	L	Y	T	R	A	E	G	D	M	1	0	4	5
1.25+402		V	E	G	Y	L	M	K	T	C	M	W	L	T	V	L	A	K	E	L	V	R	N	L	L	V	G	T	Y	G	L	M	I	A	V	I	V	R	L	M	N	D	I	V	S	H	R	F	C	E	V	L	Y	T	R	A	E	G	D	M	1	1	3	5
1.25+467		V	E	G	Y	L	M	K	T	C	M	W	L	T	V	L	A	K	E	L	V	R	N	L	L	V	G	T	Y	G	L	M	I	A	V	I	G	R	L	M	N	D	I	V	S	H	R	F	C	E	V	L	Y	T	R	A	E	G	D	M	1	1	4	6
1.34-402		V	E	G	Y	L	M	K	T	C	A	W	L	V	V	L	A	K	E	L	V	R	N	Y	L	V	G	G	Y	G	L	M	I	A	V	I	V	R	L	L	N	D	I	V	S	H	R	M	C	E	V	L	Y	T	H	A	E	G	D	M	1	1	2	4
1.34+402		V	E	G	Y	L	M	K	T	C	A	W	L	V	V	L	A	K	E	L	V	R	N	L	L	V	G	G	Y	G	L	M	I	A	V	I	V	R	L	L	N	D	I	V	S	H	R	M	C	E	V	L	Y	T	H	A	E	G	D	M	1	2	2	5
1.34+467		V	E	G	Y	L	M	K	T	C	A	W	L	V	V	L	A	K	E	L	V	R	N	L	L	V	G	G	Y	G	L	M	I	A	V	I	G	R	L	L	N	D	I	V	S	H	R	M	C	E	V	L	Y	T	H	A	E	G	D	M	1	2	3	6
1.37-402		V	E	G	Y	L	M	K	T	C	A	W	L	V	V	L	A	K	E	F	V	R	N	Y	L	V	G	T	A	G	L	M	I	A	V	I	G	R	L	M	N	D	I	V	S	H	R	M	C	E	V	L	Y	T	H	A	E	G	D	M	1	2	2	5
1.37+430		V	E	G	Y	L	M	K	T	C	A	W	L	V	V	L	A	K	E	F	V	R	N	L	L	V	G	T	A	G	L	M	I	A	V	I	G	R	L	M	N	D	I	V	S	H	R	M	C	E	V	L	Y	T	H	A	E	G	D	M	1	3	2	6
1.37+430		V	E	G	Y	L	M	K	T	C	A	W	L	V	V	L	A	K	E	F	V	R	N	L	L	V	G	T	A	G	L	M	I	A	V	I	G	R	L	M	N	D	I	V	S	H	R	M	C	E	V	L	Y	T	H	A	E	G	D	M	1	3	2	6
1.37+467		V	E	G	Y	L	M	K	T	C	A	W	L	V	V	L	A	K	E	F	V	R	N	L	L	V	G	T	Y	G	L	M	I	A	V	I	G	R	L	M	N	D	I	V	S	H	R	M	C	E	V	L	Y	T	H	A	E	G	D	M	1	2	2	5
2.05+402		V	E	G	Y	L	M	K	T	V	M	W	L	V	V	L	A	K	E	L	V	R	N	L	L	V	G	T	A	G	L	M	I	A	V	I	V	R	L	M	D	D	I	V	S	H	R	M	C	E	V	L	Y	T	H	A	E	G	D	M	1	2	0	3
2.09-2-402		V	E	G	Y	L	M	K	T	C	A	W	L	V	V	L	A	K	E	L	V	R	N	Y	L	V	G	T	A	G	L	M	I	A	V	I	V	R	L	M	N	D	I	V	S	H	R	F	C	E	V	L	Y	T	H	A	E	G	D	M	1	1	2	4
2.09-402		V	E	G	Y	L	M	K	T	C	M	W	L	V	L	L	A	K	E	L	V	R	N	Y	L	V	G	T	Y	G	L	M	I	A	V	I	V	R	L	M	N	D	I	V	S	H	R	M	C	E	V	L	Y	T	H	A	G	G	D	M	1	0	2	3
2.09+402		V	E	G	Y	L	M	K	T	C	M	W	L	V	L	L	A	K	E	L	V	R	N	L	L	V	G	T	Y	G	L	M	I	A	V	I	V	R	L	M	N	D	I	V	S	H	R	M	C	E	V	L	Y	T	H	A	G	G	D	M	1	1	2	4
2.09+467		V	E	G	Y	L	M	K	T	C	M	W	L	V	L	L	A	K	E	L	V	R	N	L	L	V	G	T	Y	G	L	M	I	A	V	I	G	R	L	M	N	D	I	V	S	H	R	M	C	E	V	L	Y	T	H	A	G	G	D	M	1	1	3	5
2.12+402		V	E	G	Y	L	M	K	T	C	A	W	L	V	V	L	A	K	E	F	V	R	N	L	L	V	G	T	A	G	L	M	I	A	V	I	V	R	L	M	D	D	I	V	S	H	R	F	C	E	V	Q	Y	T	H	A	E	G	D	M	1	3	2	6
2.22+402		V	E	G	Y	L	M	K	T	C	M	W	L	V	L	L	A	K	E	L	V	R	N	L	L	V	G	G	Y	G	L	M	I	A	V	I	V	R	L	M	N	D	I	V	S	H	R	M	C	E	V	L	Y	T	H	A	E	G	D	M	1	2	1	4
2.22+467		V	E	G	Y	L	M	K	T	C	M	W	L	V	L	L	A	K	E	L	V	R	N	L	L	V	G	G	Y	G	L	M	I	A	V	I	G	R	L	M	N	D	I	V	S	H	R	M	C	E	V	L	Y	T	H	A	E	G	D	M	1	2	2	5
2.28-402		V	E	G	Y	L	M	K	T	C	M	V	L	V	V	L	A	K	E	F	V	R	N	Y	L	V	G	T	Y	G	L	M	I	A	V	I	G	R	L	M	N	D	I	V	S	H	R	M	C	E	V	L	Y	T	H	A	E	G	D	M	1	1	2	4
2.28+402		V	E	G	Y	L	M	K	T	C	M	V	L	V	V	L	A	K	E	F	V	R	N	L	L	V	G	T	Y	G	L	M	I	A	V	I	V	R	L	M	D	D	I	V	S	H	R	M	C	E	V	L	Y	T	H	A	E	G	D	M	1	2	0	3
2.28+430		V	E	G	Y	L	M	K	T	C	M	V	L	V	V	L	A	K	E	F	V	R	N	L	L	V	G	T	A	G	L	M	I	A	V	I	G	R	L	M	N	D	I	V	S	H	R	M	C	E	V	L	Y	T	H	A	E	G	D	M	1	3	2	6
2.28+467		V	E	G	Y	L	M	K	T	C	M	V	L	V	V	L	A	K	E	F	V	R	N	L	L	V	G	T	Y	G	L	M	I	A	V	I	G	R	L	M	N	D	I	V	S	H	R	M	C	E	V	L	Y	T	H	A	E	G	D	M	1	2	2	5
2.37-402		V	E	G	Y	L	M	K	T	C	M	V	L	V	V	L	A	K	E	L	V	R	N	Y	L	V	G	T	Y	G	L	M	I	A	V	I	G	R	L	M	N	D	I	V	S	H	R	M	C	E	V	L	Y	T	H	A	E	G	D	M	1	0	2	3
2.37+402		V	E	G	Y	L	M	K	T	C	M	V	L	V	V	L	G	K	E	L	V	R	N	L	L	V	G	T	Y	G	L	M	I	A	V	I	V	R	L	M	D	D	I	V	S	H	R	M	C	E	V	L	Y	T	H	A	E	G	D	M	1	2	0	3
2.37+430		V	E	G	Y	L	M	K	T	C	M	V	L	V	V	L	A	K	E	L	V	R	N	L	L	V	G	T	A	G	L	M	I	A	V	I	G	R	L	M	N	D	I	V	S	H	R	M	C	E	V	L	Y	T	H	A	E	G	D	M	1	2	2	5
2.37+467		V	E	G	Y	L	M	K	T	C	M	V	L	V	V	L	A	K	E	L	V	R	N	L	L	V	G	T	Y	G	L	M	I	A	V	I	G	R	L	M	N	D	I	V	S	H	R	M	C	E	V	L	Y	T	H	A	E	G	D	M	1	1	2	4
2.39-402		V	E	G	Y	L	M	K	T	C	A	W	L	V	V	L	A	K	E	L	V	R	N	Y	L	V	G	T	A	G	L	M	I	A	V	I	V	R	L	M	D	D	I	V	T	H	R	M	C	E	V	L	Y	T	H	A	E	G	D	M	1	1	1	3
2.39+402		V	E	G	Y	L	M	K	T	C	A	W	L	V	V	L	A	K	E	L	V	R	N	L	L	V	G	T	A	G	L	M	I	A	V	I	V	R	L	M	D	D	I	V	T	H	R	M	C	E	V	L	Y	T	H	A	E	G	D	M	1	2	1	4
2.39+430		V	E	G	Y	L	M	K	T	C	A	W	L	V	V	L	A	K	E	L	V	R	N	L	L	V	G	T	A	G	L	M	I	A	V	I	G	R	L	M	D	D	I	V	T	H	R	M	C	E	V	L	Y	T	H	A	E	G	D	M	1	2	2	5
2.41-402		V	E	G	Y	L	M	K	T	C	M	W	L	V	L	L	A	K	E	F	V	R	N	Y	L	V	G	T	Y	G	L	M	I	A	V	I	V	R	L	L	N	D	I	V	S	H	R	M	C	E	V	L	Y	T	H	A	E	G	D	M	1	1	2	4
2.41+296		V	E	C	Y	L	M	K	T	C	M	W	L	V	L	L	A	K	E	F	V	R	N	L	L	V	G	T	Y	G	L	M	I	A	V	I	V	R	L	L	N	D	I	V	S	H	R	M	C	E	V	L	Y	T	H	A	E	G	D	M	2	2	2	6
2.41+402		V	E	G	Y	L	M	K	T	C	M	W	L	V	L	L	A	K	E	F	V	R	N	L	L	V	G	T	Y	G	L	M	I	A	V	I	V	R	L	L	N	D	I	V	S	H	R	M	C	E	V	L	Y	T	H	A	E	G	D	M	1	2	2	5
2.46+402		V	E	G	Y	L	M	K	T	C	M	V	L	V	V	L	A	K	E	F	V	R	N	L	L	V	G	T	Y	G	L	L	I	A	V	I	V	R	L	M	D	D	I	V	S	H	R	M	C	E	V	L	Y	T	H	A	E	G	D	M	1	3	0	4
2.46+430		V	E	G	Y	L	M	K	T	C	M	V	L	V	V	L	A	K	E	F	V	R	N	L	L	V	G	T	A	G	L	L	I	A	V	I	G	R	L	M	N	D	I	V	S	H	R	M	C	E	V	L	Y	T	H	A	E	G	D	M	1	4	2	7
2.46+467		V	E	G	Y	L	M	K	T	C	M	V	L	V	V	L	A	K	E	F	V	R	N	L	L	V	G	T	Y	G	L	L	I	A	V	I	G	R	L	M	N	D	I	V	S	H	R	M	C	E	V	L	Y	T	H	A	E	G	D	M	1	3	2	6
2.61-402		V	E	G	Y	L	M	K	A	C	M	W	L	V	V	L	A	K	E	F	V	R	N	Y	L	V	G	G	Y	G	L	M	I	A	V	I	V	R	L	M	D	D	I	V	T	H	R	M	C	E	V	L	Y	T	H	A	E	G	D	M	1	2	1	4
2.61+402		V	E	G	Y	L	M	K	A	C	M	W	L	V	V	L	A	K	E	F	V	R	N	L	L	V	G	G	Y	G	L	M	I	A	V	I	V	R	L	M	D	D	I	V	T	H	R	M	C	E	V	L	Y	T	H	A	E	G	D	M	1	3	1	5
2.61+467		V	E	G	Y	L	M	K	A	C	M	W	L	V	V	L	A	K	E	F	V	R	N	L	L	V	G	G	Y	G	L	M	I	A	V	I	G	R	L	M	D	D	I	V	T	H	R	M	C	E	V	L	Y	T	H	A	E	G	D	M	1	3	2	6
3.03-402		V	E	G	Y	L	M	K	T	C	A	W	L	V	V	L	A	K	E	F	V	R	N	Y	L	V	G	T	A	G	L	M	I	A	V	I	V	R	L	L	N	D	I	V	S	H	R	M	C	E	V	L	Y	T	H	A	E	G	D	M	1	2	2	5
3.03+402		V	E	G	Y	L	M	K	T	C	A	W	L	V	V	L	A	K	E	F	V	R	N	L	L	V	G	T	A	G	L	M	I	A	V	I	V	R	L	L	N	D	I	V	S	H	R	M	C	E	V	L	Y	T	H	A	E	G	D	M	1	3	2	6
3.05+402		V	E	G	Y	L	M	K	T	C	M	W	I	V	V	L	A	K	E	F	V	R	N	L	L	V	G	T	A	G	L	M	I	A	V	I	V	R	L	L	N	D	I	V	S	H	R	M	C	E	V	L	Y	T	H	A	E	G	D	M	1	3	2	6
3.05+430		V	E	G	Y	L	M	K	T	C	M	W	I	V	V	L	A	K	E	F	V	R	N	L	L	V	G	T	A	G	L	M	I	A	V	I	G	R	L	L	N	D	I	V	S	H	R	M	C	E	V	L	Y	T	H	A	E	G	D	M	1	3	3	7
3.10-402		V	E	G	Y	L	M	K	T	C	A	W	L	V	V	L	A	K	E	F	V	R	N	Y	L	V	G	G	Y	G	L	M	I	A	V	I	G	R	L	M	N	D	I	V	S	H	R	M	C	E	V	L	Y	T	H	A	E	G	D	M	1	2	2	5
3.10+402		V	E	G	Y	L	M	K	T	C	A	W	L	V	V	L	A	K	E	F	V	R	N	L	L	V	G	G	Y	G	L	M	I	A	V	I	V	R	L	M	D	D	I	V	S	H	R	M	C	E	V	L	Y	T	H	A	E	G	D	M	1	3	0	4
3.10+430		V	E	G	Y	L	M	K	T	C	A	W	L	V	V	L	A	K	E	F	V	R	N	L	L	V	G	G	A	G	L	M	I	A	V	I	G	R	L	M	N	D	I	V	S	H	R	M	C	E	V	L	Y	T	H	A	E	G	D	M	1	4	2	7
3.10+467		V	E	G	Y	L	M	K	T	C	A	W	L	V	V	L	A	K	E	F	V	R	N	L	L	V	G	G	Y	G	L	M	I	A	V	I	G	R	L	M	N	D	I	V	S	H	R	M	C	E	V	L	Y	T	H	A	E	G	D	M	1	3	2	6
3.26-402		V	E	G	Y	L	M	K	T	C	M	V	L	V	V	L	A	K	E	L	V	R	N	Y	L	V	G	T	A	G	L	M	I	A	V	I	V	R	L	L	N	D	I	V	S	H	R	M	C	E	V	L	Y	T	H	A	E	G	D	M	1	1	2	4
3.26+402		V	E	G	Y	L	M	K	T	C	M	V	L	V	V	L	A	K	E	L	V	R	N	L	L	V	G	T	A	G	L	M	I	A	V	I	V	R	L	L	N	D	I	V	S	H	R	M	C	E	V	L	Y	T	H	A	E	G	D	M	1	2	2	5
3.26+467		V	E	G	Y	L	M	K	T	C	M	V	L	V	V	L	A	K	E	L	V	R	N	L	L	V	G	T	A	G	L	M	I	A	V	I	G	R	L	L	N	D	I	V	S	H	R	M	C	E	V	L	Y	T	H	A	E	G	D	M	1	2	3	6
3.29-402		V	E	G	Y	L	M	K	T	C	M	W	L	V	L	L	A	K	E	L	V	R	N	Y	L	V	G	G	Y	G	L	M	I	A	V	I	V	R	L	M	D	D	I	V	S	H	R	M	C	E	V	L	Y	T	H	A	G	G	D	M	1	1	1	3
3.29+402		V	E	G	Y	L	M	K	T	C	M	W	L	V	L	L	A	K	E	L	V	R	N	L	L	V	G	G	Y	G	L	M	I	A	V	I	V	R	L	M	D	D	I	V	S	H	R	M	C	E	V	L	Y	T	H	A	G	G	D	M	1	2	1	4
3.36+402		V	E	G	Y	L	M	K	T	C	M	W	I	V	V	L	A	K	E	F	V	R	N	L	L	V	G	T	A	G	L	M	I	A	V	I	V	R	L	M	D	D	I	V	S	H	R	M	C	E	V	L	Y	T	H	A	E	G	D	M	1	3	0	4
3.36+430		V	E	G	Y	L	M	K	T	C	M	W	I	V	V	L	A	K	E	F	V	R	N	L	L	V	G	T	A	G	L	M	I	A	V	I	G	R	L	M	N	D	I	V	S	H	R	M	C	E	V	L	Y	T	H	A	E	G	D	M	1	3	2	6
3.36+467		V	E	G	Y	L	M	K	T	C	M	W	I	V	V	L	A	K	E	F	V	R	N	L	L	V	G	T	Y	G	L	M	I	A	V	I	G	R	L	M	N	D	I	V	S	H	R	M	C	E	V	L	Y	T	H	A	E	G	D	M	1	2	2	5
4.02+467		V	E	G	Y	L	M	K	T	C	M	W	L	V	V	L	A	K	E	F	V	R	N	L	L	V	G	T	Y	G	L	M	I	A	V	I	G	R	L	M	D	D	I	V	T	H	R	F	C	E	V	L	Y	T	R	A	E	G	D	M	0	2	4	6
4.12-402		V	E	G	Y	L	M	K	T	C	M	W	L	V	V	L	A	K	E	L	V	R	N	Y	L	V	G	T	A	G	L	M	I	A	V	I	V	R	L	M	D	D	I	V	S	H	R	M	C	E	V	L	Y	T	R	A	G	G	D	M	0	1	2	3
4.12+402		V	E	G	Y	L	M	K	T	C	M	W	L	V	V	L	A	K	E	L	V	R	N	L	L	V	G	T	A	G	L	M	I	A	V	I	V	R	L	M	D	D	I	V	S	H	R	M	C	E	V	L	Y	T	R	A	G	G	D	M	0	2	2	4
5.01+402		V	E	G	Y	L	M	K	A	C	M	W	L	V	V	L	A	K	E	L	V	R	N	L	L	V	G	G	Y	G	L	M	I	A	V	I	V	R	L	M	D	D	I	V	S	H	R	M	C	E	V	L	Y	T	R	A	G	G	D	M	1	2	2	5
5.24+402		V	E	G	Y	L	M	K	A	C	M	W	L	V	V	L	A	K	E	L	V	R	N	L	L	V	G	G	Y	G	L	M	I	A	V	I	V	R	L	M	D	D	I	V	S	H	R	M	C	E	V	L	Y	T	R	A	G	G	D	M	1	2	2	5
5.24+467		V	E	G	Y	L	M	K	A	C	M	W	L	V	V	L	A	K	E	L	V	R	N	L	L	V	G	G	Y	G	L	M	I	A	V	I	G	R	L	M	D	D	I	V	S	H	R	M	C	E	V	L	Y	T	R	A	G	G	D	M	1	2	3	6
7.02o-402		V	E	G	Y	L	M	K	T	C	M	W	L	V	L	L	A	K	E	L	V	R	N	Y	L	V	G	T	Y	G	L	M	I	A	V	I	V	R	L	M	D	D	I	V	S	H	R	M	C	E	V	L	Y	T	R	A	E	G	E	M	1	0	2	3
7.02o+402		V	E	G	Y	L	M	K	T	C	M	W	L	V	L	L	A	K	E	L	V	R	N	L	L	V	G	T	Y	G	L	M	I	A	V	I	V	R	L	M	D	D	I	V	S	H	R	M	C	E	V	L	Y	T	R	A	E	G	E	M	1	1	2	4
7.02o+467		V	E	G	Y	L	M	K	T	C	M	W	L	V	L	L	A	K	E	L	V	R	N	L	L	V	G	T	Y	G	L	M	I	A	V	I	G	R	L	M	D	D	I	V	S	H	R	M	C	E	V	L	Y	T	R	A	E	G	E	M	1	1	3	5
7.09-402		V	E	G	Y	L	M	K	T	C	M	W	L	T	V	L	A	K	E	L	V	R	N	Y	L	V	G	T	Y	G	L	L	I	A	V	I	V	R	L	M	D	D	I	V	S	H	R	F	C	E	V	Q	Y	T	H	A	E	G	D	M	1	1	2	4
7.09+402		V	E	G	Y	L	M	K	T	C	M	W	L	T	V	L	A	K	E	L	V	R	N	L	L	V	G	T	Y	G	L	L	I	A	V	I	V	R	L	M	D	D	I	V	S	H	R	F	C	E	V	Q	Y	T	H	A	E	G	D	M	1	2	2	5
7.09+467		V	E	G	Y	L	M	K	T	C	M	W	L	T	V	L	A	K	E	L	V	R	N	L	L	V	G	T	Y	G	L	L	I	A	V	I	G	R	L	M	D	D	I	V	S	H	R	F	C	E	V	Q	Y	T	H	A	E	G	D	M	1	2	3	6
7.10-402		V	E	G	Y	L	M	K	T	C	M	W	L	V	V	L	A	K	E	L	V	R	N	Y	L	V	G	T	Y	A	L	L	I	A	V	I	V	R	L	L	N	D	I	V	S	H	R	M	L	E	V	L	Y	T	R	A	E	G	D	M	0	2	4	6
7.10+402		V	E	G	Y	L	M	K	T	C	M	W	L	V	V	L	A	K	E	L	V	R	N	L	L	V	G	T	Y	A	L	L	I	A	V	I	V	R	L	L	N	D	I	V	S	H	R	M	L	E	V	L	Y	T	R	A	E	G	D	M	0	3	4	7
7.10+467		V	E	G	Y	L	M	K	T	C	M	W	L	V	V	L	A	K	E	L	V	R	N	L	L	V	G	T	Y	A	L	L	I	A	V	I	G	R	L	L	N	D	I	V	S	H	R	M	L	E	V	L	Y	T	R	A	E	G	D	M	0	3	5	8
7.11-402		V	E	G	Y	L	M	K	T	C	M	W	L	V	L	L	A	K	E	L	V	R	N	Y	L	V	G	T	A	G	L	M	I	A	V	I	V	R	L	M	D	D	I	V	S	H	R	M	C	E	V	L	Y	T	R	A	E	G	E	M	1	1	2	4
7.11+402		V	E	G	Y	L	M	K	T	C	M	W	L	V	L	L	A	K	E	L	V	R	N	L	L	V	G	T	A	G	L	M	I	A	V	I	V	R	L	M	D	D	I	V	S	H	R	M	C	E	V	L	Y	T	R	A	E	G	E	M	1	2	2	5
7.27-402		V	E	G	Y	L	M	K	T	C	M	V	L	V	V	L	A	K	E	L	V	R	N	Y	L	V	G	T	Y	G	L	M	I	A	V	I	G	R	L	M	N	D	I	V	S	H	R	F	C	E	V	L	Y	T	R	A	E	G	D	M	1	0	4	5
7.27+430		V	E	G	Y	L	M	K	T	C	M	V	L	V	V	L	A	K	E	L	V	R	N	L	L	V	G	T	A	G	L	M	I	A	V	I	G	R	L	M	N	D	I	V	S	H	R	F	C	E	V	L	Y	T	R	A	E	G	D	M	1	2	4	7
7.27+467		V	E	G	Y	L	M	K	T	C	M	V	L	V	V	L	A	K	E	L	V	R	N	L	L	V	G	T	Y	G	L	M	I	A	V	I	G	R	L	M	N	D	I	V	S	H	R	F	C	E	V	L	Y	T	R	A	E	G	D	M	1	1	4	6
7.27o-402		V	E	G	Y	L	M	K	T	C	M	W	L	V	V	L	A	K	E	L	V	R	N	Y	L	V	G	T	Y	G	L	L	I	A	V	I	V	R	L	M	D	D	I	V	S	H	R	F	C	E	V	Q	Y	T	H	A	E	G	D	M	0	1	2	3
7.27o+402		V	E	G	Y	L	M	K	T	C	M	W	L	V	V	L	A	K	E	L	V	R	N	L	L	V	G	T	Y	G	L	L	I	A	V	I	V	R	L	M	D	D	I	V	S	H	R	F	C	E	V	Q	Y	T	H	A	E	G	D	M	0	2	2	4

Supplementary Table 3 continued

Mutant name	Number of mutations	Kinetic parameters: Protein concentration (μM)	Kinetic parameters: kcat apparent (min^{-1})	Kinetic parameters: % of AaFS-WT activity	Product analysis: Linear products (%)	Product analysis: Cyclic products (%)	Background 402	Background 467	Background 430
7.27o+467	5	2.80	1.18	3.81	92	8	L	G	Y
7.30o-402	7	1.63	0.90	2.88	94	6	Y	V	A
7.30o+402	8	2.86	1.12	3.59	26	74	L	V	A
7.30o+467	9	2.35	0.93	2.98	73	27	L	G	A
8.15-402	3	2.95	0.84	2.46	98	2	Y	V	Y
8.15+402	4	3.09	0.56	1.64	65	35	L	V	Y
8.15+467	5	3.23	0.63	1.84	89	11	L	G	Y
9.31+402	10	2.50	1.10	3.70	16	84	L	V	A
AaFS-WT-10	0	2.95	37.45	100.00	99	1	Y	V	Y
AaFS-402	1	2.78	32.97	77.69	23	77	L	V	Y
WT+402	1	2.86	24.10	77.14	22	78	L	V	Y
WT+467	2	2.94	0.82	2.62	84	16	L	G	Y
AaFS-467	1	2.85	2.48	5.84	90	10	Y	G	Y
AaFS-430	1	2.35	21.55	50.78	98	2	Y	V	A
AaFS-402/430	2	2.78	19.14	45.10	12	88	L	V	A
AaFS-402/467	2	2.98	0.38	0.90	86	14	L	G	Y
AaFS-467/430	2	2.95	8.37	19.72	96	4	Y	G	A
AaFS-402/467/430	3	3.42	0.00	1.08	45	55	L	G	A

	Zone 1				Zone 2											Zone 3									
Residue	294	295	296	297	316	317	318	319	320	321	322	323	324	325	326	395	396	397	398	399	400	401	402	403	404
AaFS	V	E	G	Y	L	M	K	T	C	M	W	L	V	V	L	A	K	E	L	V	R	N	Y	L	V
AaADS	V	E	C	Y	F	T	K	A	V	A	V	I	T	L	I	V	K	E	F	V	R	N	L	M	V
7.27o+467	V	E	G	Y	L	M	K	T	C	M	W	L	V	V	L	A	K	E	L	V	R	N	L	L	V
7.30o-402	V	E	G	Y	L	M	K	T	C	A	W	L	V	V	L	A	K	E	L	V	R	N	Y	L	V
7.30o+402	V	E	G	Y	L	M	K	T	C	A	W	L	V	V	L	A	K	E	L	V	R	N	L	L	V
7.30o+467	V	E	G	Y	L	M	K	T	C	A	W	L	V	V	L	A	K	E	L	V	R	N	L	L	V
8.15-402	V	E	G	Y	L	M	K	T	C	M	W	L	V	L	L	A	K	E	L	V	R	N	Y	L	V
8.15+402	V	E	G	Y	L	M	K	T	C	M	W	L	V	L	L	A	K	E	L	V	R	N	L	L	V
8.15+467	V	E	G	Y	L	M	K	T	C	M	W	L	V	L	L	A	K	E	L	V	R	N	L	L	V
9.31+402	V	E	G	Y	L	M	K	T	C	M	W	L	T	L	L	A	K	E	L	V	R	N	L	L	V
AaFS-WT-10	V	E	G	Y	L	M	K	T	C	M	W	L	V	V	L	A	K	E	L	V	R	N	Y	L	V
AaFS-402	V	E	G	Y	L	M	K	T	C	M	W	L	V	V	L	A	K	E	L	V	R	N	L	L	V
WT+402	V	E	G	Y	L	M	K	T	C	M	W	L	V	V	L	A	K	E	L	V	R	N	L	L	V
WT+467	V	E	G	Y	L	M	K	T	C	M	W	L	V	V	L	A	K	E	L	V	R	N	L	L	V
AaFS-467	V	E	G	Y	L	M	K	T	C	M	W	L	V	V	L	A	K	E	L	V	R	N	Y	L	V
AaFS-430	V	E	G	Y	L	M	K	T	C	M	W	L	V	V	L	A	K	E	L	V	R	N	Y	L	V
AaFS-402/430	V	E	G	Y	L	M	K	T	C	M	W	L	V	V	L	A	K	E	L	V	R	N	L	L	V
AaFS-402/467	V	E	G	Y	L	M	K	T	C	M	W	L	V	V	L	A	K	E	L	V	R	N	L	L	V
AaFS-467/430	V	E	G	Y	L	M	K	T	C	M	W	L	V	V	L	A	K	E	L	V	R	N	Y	L	V
AaFS-402/467/430	V	E	G	Y	L	M	K	T	C	M	W	L	V	V	L	A	K	E	L	V	R	N	L	L	V

	Zone 4								Zone 5												Zone 6														Total number of mutations			
Residue	428	429	430	431	432	433	434	435	465	466	467	468	469	470	471	472	473	474	475	476	541	542	543	544	545	546	547	554	555	556	557	558	559	560				
AaFS	G	T	Y	G	L	M	I	A	V	I	V	R	L	M	D	D	I	V	S	H	R	M	C	E	V	L	Y	T	H	A	E	G	D	M				
AaADS	G	G	A	N	L	L	T	T	I	L	G	R	R	L	N	D	L	M	T	H	Q	F	L	E	V	Q	Y	T	R	M	G	D	E	Y	N	T	C	Total
7.27o+467	G	T	Y	G	L	L	I	A	V	I	G	R	L	M	D	D	I	V	S	H	R	F	C	E	V	Q	Y	T	H	A	E	G	D	M	0	2	3	5
7.30o-402	G	T	A	G	L	M	I	A	V	I	V	R	L	L	N	D	I	V	S	H	R	F	L	E	V	L	Y	T	H	A	G	G	D	M	1	1	5	7
7.30o+402	G	T	A	G	L	M	I	A	V	I	V	R	L	L	N	D	I	V	S	H	R	F	L	E	V	L	Y	T	H	A	G	G	D	M	1	2	5	8
7.30o+467	G	T	A	G	L	M	I	A	V	I	G	R	L	L	N	D	I	V	S	H	R	F	L	E	V	L	Y	T	H	A	G	G	D	M	1	2	6	9
8.15-402	G	T	Y	G	L	M	I	A	V	I	V	R	L	M	D	D	I	V	S	H	R	F	C	E	V	L	Y	T	R	A	E	G	D	M	1	0	2	3
8.15+402	G	T	Y	G	L	M	I	A	V	I	V	R	L	M	D	D	I	V	S	H	R	F	C	E	V	L	Y	T	R	A	E	G	D	M	1	1	2	4
8.15+467	G	T	Y	G	L	M	I	A	V	I	G	R	L	M	D	D	I	V	S	H	R	F	C	E	V	L	Y	T	R	A	E	G	D	M	1	1	3	5
9.31+402	G	G	A	G	L	M	I	A	V	I	V	R	L	L	N	D	I	V	S	H	R	F	C	E	V	Q	Y	T	R	A	E	G	D	M	2	3	5	10
AaFS-WT-10	G	T	Y	G	L	M	I	A	V	I	V	R	L	M	D	D	I	V	S	H	R	M	C	E	V	L	Y	T	H	A	E	G	D	M	0	0	0	0
AaFS-402	G	T	Y	G	L	M	I	A	V	I	V	R	L	M	D	D	I	V	S	H	R	M	C	E	V	L	Y	T	H	A	E	G	D	M	0	1	0	1
WT+402	G	T	Y	G	L	M	I	A	V	I	V	R	L	M	D	D	I	V	S	H	R	M	C	E	V	L	Y	T	H	A	E	G	D	M	0	1	0	1
WT+467	G	T	Y	G	L	M	I	A	V	I	G	R	L	M	D	D	I	V	S	H	R	M	C	E	V	L	Y	T	H	A	E	G	D	M	0	1	1	2
AaFS-467	G	T	Y	G	L	M	I	A	V	I	G	R	L	M	D	D	I	V	S	H	R	M	C	E	V	L	Y	T	H	A	E	G	D	M	0	0	1	1
AaFS-430	G	T	A	G	L	M	I	A	V	I	V	R	L	M	D	D	I	V	S	H	R	M	C	E	V	L	Y	T	H	A	E	G	D	M	0	1	0	1
AaFS-402/430	G	T	A	G	L	M	I	A	V	I	V	R	L	M	D	D	I	V	S	H	R	M	C	E	V	L	Y	T	H	A	E	G	D	M	0	2	0	2
AaFS-402/467	G	T	Y	G	L	M	I	A	V	I	G	R	L	M	D	D	I	V	S	H	R	M	C	E	V	L	Y	T	H	A	E	G	D	M	0	1	1	2
AaFS-467/430	G	T	A	G	L	M	I	A	V	I	G	R	L	M	D	D	I	V	S	H	R	M	C	E	V	L	Y	T	H	A	E	G	D	M	0	1	1	2
AaFS-402/467/430	G	T	A	G	L	M	I	A	V	I	G	R	L	M	D	D	I	V	S	H	R	M	C	E	V	L	Y	T	H	A	E	G	D	M	0	2	1	3

Supplementary Table 4. Summary of BFS 6Å library screening results

a. Results from screening of the BFS 6Å library. The 1st screen rows detail the results from the initial screen of the BFS library. The second screen rows detail the results from the focused libraries generated to search for alternative position(s) that may activate cyclization (Y402 background) or to search for more functionally diverse cyclases (Y402L background). **b.** SCOPE grid used for gene reconstruction. **c.** Number of unique mutagenized fragments generated for each gene fragment at low, medium and high mutation levels. **d.** Numbering of BFS library pools for screening. Fragment combinations for each pool are detailed in panel B. **e.** Total number of possible independent mutation combinations for each pool. This number was generated by multiplying together the number of unique mutagenised fragments for each fragment combination. For example, for pool 5 - 7 (N-terminal MED) x 6 (Central MED) x 65 (C-terminal MED) = 2730.

a

BFS library pool	No. of mutants assayed	Inactive mutants	Active mutants	% Active mutants	Active farnesene synthase mutants				Active cyclase mutants			
					High	Low	Trace	**Total**	High	Low	Trace	**Total**
1st screen - POOL1	31	26	5	16	0	4	1	5	0	0	0	0
1st screen - POOL2	31	30	1	3	0	1	0	1	0	0	0	0
1st screen - POOL3	32	32	0	0	0	0	0	0	0	0	0	0
1st screen - POOL4	31	22	9	29	2	1	6	9	0	0	0	0
1st screen - POOL5	31	31	0	0	0	0	0	0	0	0	0	0
1st screen - POOL6	32	32	0	0	0	0	0	0	0	0	0	0
1st screen - POOL7	31	28	3	10	0	0	0	0	1	2	0	3
1st screen - POOL8	31	31	0	0	0	0	0	0	0	0	0	0
1st screen - POOL9	32	32	0	0	0	0	0	0	0	0	0	0
Total	282	264	18	6	2	6	7	15	1	2	0	3
2nd screen (Y402) - POOL1	16	9	7	44	6	0	1	7	0	0	0	0
2nd screen (Y402) - POOL2	16	12	4	25	3	0	1	4	0	0	0	0
2nd screen (Y402) - POOL3	16	9	7	44	5	0	2	7	0	0	0	0
2nd screen (Y402) - POOL4	15	14	1	7	1	0	0	1	0	0	0	0
2nd screen (Y402) - POOL5	16	16	0	0	0	0	0	0	0	0	0	0
2nd screen (Y402) - POOL6	16	16	0	0	0	0	0	0	0	0	0	0
2nd screen (Y402) - POOL7	15	11	4	27	3	0	1	4	0	0	0	0
2nd screen (Y402) - POOL8	16	14	2	13	2	0	0	2	0	0	0	0
2nd screen (Y402) - POOL9	16	16	0	0	0	0	0	0	0	0	0	0
Total	142	117	25	18	20	0	5	25	0	0	0	0
2nd screen (Y402L) - POOL1	39	31	8	21	1	3	0	4	2	2	0	4
2nd screen (Y402L) - POOL2	65	49	16	25	1	4	0	5	9	2	0	11
2nd screen (Y402L) - POOL3	38	30	8	21	0	0	0	0	4	3	1	8
2nd screen (Y402L) - POOL4	40	38	2	5	0	0	0	0	0	2	0	2
2nd screen (Y402L) - POOL5	38	31	7	18	0	0	0	0	2	4	1	7
2nd screen (Y402L) - POOL6	15	15	0	0	0	0	0	0	0	0	0	0
2nd screen (Y402L) - POOL7	39	34	5	13	0	1	0	1	1	2	1	4
2nd screen (Y402L) - POOL8	15	15	0	0	0	0	0	0	0	0	0	0
2nd screen (Y402L) - POOL9	32	31	1	3	0	0	0	0	0	1	0	1
Total	321	274	47	15	2	8	0	10	18	16	3	37
**TOTAL**	**745**	**655**	**90**	**12**	**24**	**14**	**12**	**50**	**19**	**18**	**3**	**40**

b

		**N-terminal fragment**		
		LOW	MED	HIGH
**C-terminal fragment**	LOW	LOW	LOW	MED
	MED	LOW	MED	HIGH
	HIGH	MED	HIGH	HIGH
		**Central fragment**		

c

Level of mutagenesis	**Number of unique mutagenised fragments**		
	N-terminal	Central	C-terminal
LOW	16	5	22
MED	7	6	65
HIGH	13	4	68

d

1<br>2-5 mutations	2<br>3-5 mutations	3<br>4-7 mutations
4<br>4-6 mutations	5<br>5-7 mutations	6<br>6-9 mutations
7<br>6-9 mutations	8<br>5-10 mutations	9<br>7-11 mutations

e

1760	770	1716
5200	2730	3380
6528	1904	3536

Supplementary Table 5. Sesquiterpene products synthesized by the BFS 6Å library
For each sesquiterpene product the retention time is listed together with the method of product verification, either by a match in the NIST/Massfinder spectral library, or by a match to an authentic standard. The origin of each authentic standard is also listed.

PRODUCT NAME	RETENTION TIME (MIN)	PRODUCT VERIFICATION		PRODUCT OBSERVED IN *Artemisia* SPECIES
		METHOD	STANDARD USED	
sesquithujene	7.765	NIST/Massfinder	-	
α-cedrene	8.260	NIST/Massfinder	-	
Unknown 1	8.470	Unknown product	-	
α-exo-bergamotene	8.519	NIST/Massfinder	-	
Peak 1	8.629	Unknown product	-	
(E)-β-farnesene	8.754	Authentic standard	Sigma (73492)	Yes[2-4]
putative amorphadiene isomer	8.997	NIST/Massfinder	-	
γ-curcumene	9.241	Authentic standard	Ginger (hexane extract)	
ar-curcumene	9.286	Authentic standard	Ginger (hexane extract)	Yes[4]
cis-α-bergamotene	9.389	NIST/Massfinder	-	
zingiberene	9.493	Authentic standard	Ginger (hexane extract)	
α-farnesene	9.642	Authentic standard	Green apple (hexane extract)	Yes[2-4]
β-bisabolene	9.731	Authentic standard	Sigma (14462)	
β-curcumene	9.785	Authentic standard	Ginger (hexane extract)	
Peak 2	9.891	Unknown product	-	
β-sesquiphelladrene	10.019	Authentic standard	Ginger (hexane extract)	
Peak 3	10.168	Unknown product	-	
α-bisabolene	10.322	Authentic standard	Sigma (14462)	
nerolidol	10.658	Authentic standard	Sigma (18143)	Yes[4]
Peak 4	11.272	Unknown product	-	
α-bisabolol	13.224	Authentic standard	Sigma (14462)	Yes[4]
farnesol	11.272	Authentic standard	Sigma (277541)	Yes[3]

Supplementary Table 6. Quantitative analysis of sesquiterpene products synthesized by the *A. annua* BFS 6Å library

Kinetic parameters and Background

Mutant name	Number of mutations	Protein concentration (μM)	kcat apparent (min^{-1})	% of AaFS-WT activity	Linear products (%)	Cyclic products (%)	Background 402	Background 467	Background 430	sesquithujene 7.77 min	α-cedrene 8.26 min	α-exo-bergamotene 8.52 min	Peak 1 8.62 min	(E)-β-farnesene 8.75 min	amorphadiene 9.00 min
**AaFS 6Å library**															
1.03-402	3	2.38	0.60	2.02	93	7	Y	V	Y				0.12	78.54	0.01
1.16-402	4	2.40	1.19	3.48	98	2	Y	V	Y				0.06	94.68	0.05
1.34-402	4	2.61	0.88	2.47	94	6	Y	V	Y			0.45	0.15	88.48	0.06
2.09-402	3	3.87	1.74	4.00	98	2	Y	V	Y	0.01		0.01	0.09	96.64	0.02
2.41-402	4	2.07	3.04	8.57	97	3	Y	V	Y				0.13	90.53	0.01
2.61-402	4	2.93	2.74	7.72	92	8	Y	V	Y	0.01		2.30	0.28	86.13	0.11
3.29-402	3	3.08	2.00	6.45	94	6	Y	V	Y			0.35	0.30	88.64	0.10
7.02o-402	3	2.75	3.28	9.25	98	2	Y	V	Y				0.13	95.11	0.03
7.09-402	4	2.08	0.83	2.84	94	6	Y	V	Y				0.16	88.97	0.04
7.10-402	6	2.51	1.84	5.94	98	2	Y	V	Y	0.01			0.14	95.89	0.01
7.27o-402	3	2.45	13.21	37.30	98	2	Y	V	Y				0.13	97.36	0.01
8.15-402	3	2.95	0.84	2.46	98	2	Y	V	Y	0.01			0.06	94.44	0.02
AaFS-WT-10	0	2.95	37.45	100.00	99	1	Y	V	Y				0.10	97.67	0.02
1.03+402	4	3.28	0.98	3.29	69	31	L	V	Y				0.04	63.51	0.55
1.12+402	6	2.88	1.64	4.80	45	55	L	V	Y	0.31		9.32	0.43	38.19	1.56
1.16+402	5	2.54	1.72	5.02	60	40	L	V	Y			0.05	0.05	53.31	0.13
1.25+402	5	3.52	10.93	25.18	34	66	L	V	Y	0.01		0.34	0.02	31.49	0.34
1.34+402	5	1.97	1.27	4.08	29	71	L	V	Y			2.23	0.05	25.77	0.45
2.09+402	4	3.65	1.87	5.99	31	69	L	V	Y	0.01		0.63	0.03	29.03	0.42
2.22+402	4	2.75	2.94	9.86	32	68	L	V	Y			1.74	0.07	29.34	0.35
2.28+402	3	3.18	0.57	1.85	66	34	L	V	Y				0.04	63.00	0.34
2.37+402	3	3.04	1.67	3.84	38	62	L	V	Y	0.01		0.14	0.02	35.86	0.55
2.41+296	6	2.29	0.39	1.14	36	64	L	V	Y	0.02				17.37	0.03
2.41+402	5	2.20	6.22	17.55	56	44	L	V	Y	0.01		0.03	0.03	50.54	0.26
2.46+402	4	2.97	0.64	2.09	67	33	L	V	Y					51.81	0.04
2.61+402	5	2.30	8.69	24.53	25	75	L	V	Y	1.26		18.91	1.17	22.91	2.56
3.10+402	4	3.48	4.44	10.23	23	77	L	V	Y	1.23		16.96	1.11	20.07	2.10
3.29+402	4	2.73	4.72	15.22	20	80	L	V	Y	0.01		1.32	0.03	15.70	0.60
5.01+402	5	2.47	0.65	2.18	40	60	L	V	Y			0.29	0.02	26.41	0.10
5.24+402	5	2.46	0.44	1.50	44	56	L	V	Y			0.42	0.08	27.82	0.51
7.02o+402	4	2.37	1.52	4.28	56	44	L	V	Y	0.01		0.14	0.04	41.70	0.86
7.10+402	7	2.76	2.88	9.29	53	47	L	V	Y			0.05		46.44	0.08
7.27o+402	4	2.15	3.43	9.67	46	54	L	V	Y	0.01		0.02	0.04	42.08	0.57
8.15+402	4	3.09	0.56	1.64	65	35	L	V	Y	0.03		0.04		45.27	0.27
AaFS-402	1	2.78	32.97	77.69	23	77	L	V	Y			0.26	0.03	21.40	1.60
WT+402	1	2.86	24.10	77.14	22	78	L	V	Y	0.05		0.33	0.03	21.18	1.23
7.09+402	5	2.67	0.00	0.00	77	23	L	V	Y				0.05	49.91	0.14
1.25-402	5	3.41	2.70	6.21	95	5	Y	G	Y	0.01		0.04	0.11	93.26	0.09
2.28-402	4	2.90	0.72	2.36	98	2	Y	G	Y	0.01			0.19	94.74	0.01
2.37-402	3	3.01	2.47	5.69	97	3	Y	G	Y	0.01		0.01	0.09	95.81	0.06
3.10-402	5	2.12	1.54	3.54	95	5	Y	G	Y	0.01		0.02	0.09	87.63	0.09
7.27-402	5	2.51	0.77	2.52	96	4	Y	G	Y				0.04	93.57	0.06
AaFS-467	1	2.85	2.48	5.84	90	10	Y	G	Y			0.02	0.11	86.37	0.50
2.09-2-402	4	2.06	1.03	3.00	98	2	Y	V	A			0.04	0.07	95.30	0.06
2.39-402	3	2.46	7.61	21.49	98	2	Y	V	A			0.01	0.13	96.83	0.02
3.03-402	5	2.52	1.98	6.38	98	2	Y	V	A	0.02			0.19	96.63	0.05
3.26-402	4	2.15	1.07	3.43	95	5	Y	V	A	0.01		0.03	0.14	91.63	0.11
4.12-402	3	2.77	3.03	9.77	97	3	Y	V	A	0.01		0.01	0.17	95.33	0.03
7.11-402	4	2.05	1.02	3.52	96	4	Y	V	A	0.01		0.01	0.11	89.51	0.03
7.30o-402	7	1.63	0.90	2.88	94	6	Y	V	A			0.03	0.16	87.42	0.21
AaFS-430	1	2.35	21.55	50.78	98	2	Y	V	A			0.02	0.09	96.77	0.04
1.16+467	6	3.19	0.35	1.01	87	13	L	G	Y	0.11		0.02		51.74	0.02
1.25+467	6	3.42	1.03	2.37	89	11	L	G	Y	0.01		0.26	0.09	84.63	0.11
1.34+467	6	2.69	0.29	0.97	78	22	L	G	Y	0.01		0.15	0.12	63.88	0.22
1.37+467	5	2.67	1.23	3.92	91	9	L	G	Y			0.02	0.10	88.83	0.05
2.09+467	5	3.47	3.41	7.86	95	5	L	G	Y	0.01		0.03	0.07	94.30	0.04
2.22+467	5	2.84	0.78	2.62	81	19	L	G	Y			0.05	0.13	67.76	0.05
2.28+467	5	2.80	0.91	2.98	95	5	L	G	Y				0.05	93.05	0.01
2.37+467	4	3.41	1.50	3.46	94	6	L	G	Y	0.01		0.01	0.11	90.64	0.04
2.46+467	6	2.21	0.54	1.77	90	10	L	G	Y				0.03	86.04	0.01
2.61+467	6	3.64	0.82	2.65	84	16	L	G	Y	0.01		1.61	0.03	72.11	0.07
3.10+467	6	3.14	0.32	0.75	87	13	L	G	Y	0.06		0.49	0.14	69.14	0.12
3.36+467	5	2.36	1.66	5.34	89	11	L	G	Y			0.03	0.08	63.76	0.09
4.02+467	6	2.48	1.43	4.61	93	7	L	G	Y				0.04	70.47	0.03
5.24+467	6	2.68	0.44	1.52	78	22	L	G	Y				0.02	43.78	0.02
7.02o+467	5	3.33	0.73	2.36	89	11	L	G	Y					62.59	
7.10+467	8	2.57	2.05	6.62	98	2	L	G	Y			0.01	0.14	96.43	0.02
7.27+467	6	3.23	0.49	1.60	94	6	L	G	Y	0.03			0.05	87.09	0.04
7.27o+467	5	2.80	1.18	3.81	92	8	L	G	Y				0.02	79.57	0.01
8.15+467	5	3.23	0.63	1.84	89	11	L	G	Y					49.86	0.09
AaFS-402/467	2	2.98	0.38	0.90	86	14	L	G	Y			0.07	0.09	83.73	0.36
WT+467	2	2.94	0.82	2.62	84	16	L	G	Y	0.01		0.11	0.09	80.16	0.34
1.03+467	6	2.70	0.30	1.01	86	14	L	G	Y				0.05	52.54	0.02
7.09+467	6	2.33	0.00	0.00	81	19	L	G	Y				0.08	48.86	0.09
2.05+402	3	2.73	0.67	2.25	60	40	L	V	A				0.04	50.53	0.05
2.12+402	6	2.45	0.99	2.90	34	66	L	V	A	0.01		0.38	0.01	25.92	0.31
2.39+402	4	2.60	8.32	23.49	13	87	L	V	A	0.01		0.89	0.01	10.50	1.83
3.03+402	6	2.52	1.03	3.30	28	72	L	V	A			0.45	0.02	18.10	0.66
3.05+402	6	2.20	0.85	2.93	62	38	L	V	A			0.49	0.03	10.25	0.83
3.26+402	5	2.48	1.70	3.92	23	77	L	V	A			0.52		21.33	0.03
3.36+402	4	2.59	0.91	2.92	59	41	L	V	A	0.02		0.31	0.04	24.44	1.10
4.12+402	4	2.85	0.84	2.71	26	74	L	V	A	0.01		0.95	0.07	18.04	1.88
7.11+402	5	2.32	0.59	2.03	42	58	L	V	A			0.24	0.02	26.49	1.20
7.30o+402	8	2.86	1.12	3.59	26	74	L	V	A	0.02		1.37	0.03	22.03	0.67
9.31+402	10	2.50	1.10	3.70	16	84	L	V	A			0.45	0.02	11.25	0.08
AaFS-402/430	2	2.78	19.14	45.10	12	88	L	V	A	0.02		0.80	0.02	9.06	1.96
1.37-402	5	1.95	1.56	5.00	95	5	Y	G	A				0.14	90.32	0.05
AaFS-467/430	2	2.95	8.37	19.72	96	4	Y	G	A				0.21	93.08	0.18
1.37+430	6	2.80	1.07	3.69	63	37	L	G	A			0.14	0.21	57.24	0.93
1.37+430	6	2.95	1.89	6.06	67	33	L	G	A			0.05	0.05	62.40	0.24
2.28+430	6	3.19	1.30	4.26	69	31	L	G	A	0.01		0.03	0.07	64.12	0.14
2.37+430	5	2.09	1.50	4.91	72	28	L	G	A			0.07	0.04	65.92	0.04
2.39+430	5	2.83	1.94	6.26	65	35	L	G	A			0.03	0.01	51.76	0.60
2.46+430	7	2.49	0.80	2.62	63	37	L	G	A				0.02	55.74	0.06
3.05+430	7	2.66	1.27	4.38	41	59	L	G	A			0.06	0.05	15.87	0.82
3.10+430	7	3.03	1.86	6.00	61	39	L	G	A			5.40	0.11	54.52	0.42
3.26+467	6	2.78	0.85	2.72	76	24	L	G	A	0.02		0.04	0.03	66.80	0.12
3.36+430	6	2.03	3.19	10.72	55	45	L	G	A			0.08		20.06	0.33
7.27+430	7	2.87	0.54	1.77	75	25	L	G	A					62.16	0.00
7.30o+467	9	2.35	0.93	2.98	73	27	L	G	A	0.01		0.08	0.06	64.34	0.18
AaFS-402/467/430	3	3.42	0.00	1.08	45	55	L	G	A			0.22	0.04	41.26	1.66

Product analysis (continued)

Mutant name	γ-curcumene 9.24 min	ar-curcumene 9.29 min	cis-α-bergamotene 9.39 min	zingiberene 9.49 min	α-farnesene 9.64 min	β-bisabolene 9.73 min	β-curcumene 9.8 min	Peak 2 9.89 min	β-sesquiphellandrene 10.02 min	α-bisabolene 10.32 min	nerolidol 10.66 min	Peak 4 11.27 min	α-bisabolol 13.22 min	farnesol 13.59 min
**AaFS 6Å library**														
1.03-402	0.01		4.38	0.05	8.74	0.53	1.12	0.02	0.21	0.09	3.87	0.10		2.23
1.16-402	0.05	0.01	1.00	0.19	1.24	0.78	0.03	0.03	0.05	0.06	1.58	0.08	0.01	0.11
1.34-402	0.12	0.01	2.10	0.25	2.96	1.05	0.15	0.13	0.47	0.29	1.06	0.09	0.88	1.30
2.09-402	0.06	0.01	0.48	0.10	0.72	0.92	0.37	0.04	0.20	0.05	0.15	0.02		0.11
2.41-402	0.03	0.01	1.93	0.03	3.31	0.52	0.19	0.02	0.00	0.04	1.41	0.06		1.77
2.61-402	0.23	0.03	2.21	0.37	2.81	0.94	0.64	0.08	0.79	0.08	1.41	0.08	0.03	1.48
3.29-402	0.11	0.01	1.88	0.20	2.94	0.96	0.12	0.08	0.78	0.25	1.35	0.07	0.92	0.94
7.02o-402	0.04		0.90	0.08	1.64	0.61	0.22	0.02	0.12	0.06	0.51	0.01		0.52
7.09-402	0.29	0.03	1.42	0.16	2.92	2.16	1.33	0.11	0.44	0.09	1.72	0.03		0.12
7.10-402	0.03		0.84	0.06	1.37	0.60	0.05	0.02	0.23	0.03	0.67	0.02		0.03
7.27o-402	0.05	0.01	0.32	0.04	0.58	0.84	0.19	0.01	0.24	0.02	0.14	0.01		0.03
8.15-402	0.03	0.01	0.75	0.06	1.26	0.68	0.12	0.03	0.43	0.07	1.88	0.04	0.01	0.12
AaFS-WT-10	0.05	0.03	0.27	0.13	0.42	0.38	0.04	0.02	0.09	0.02	0.72	0.01	0.01	0.02
1.03+402	1.40	0.67	7.59	5.80	3.78	12.28	0.60	0.09	1.30	0.14	1.12	0.86	0.13	0.13
1.12+402	4.62	1.33	14.62	6.61	4.03	5.50	2.82	1.27	2.81	0.95	2.25	1.22	2.12	0.04
1.16+402	0.63	0.51	10.50	3.69	3.18	12.26	2.05	0.47	0.75	2.52	3.03	1.56	5.03	0.28
1.25+402	1.77	1.08	5.20	6.59	2.02	12.69	7.86	2.07	1.18	3.33	0.14	0.70	23.14	0.03
1.34+402	0.87	0.17	2.28	0.89	2.45	11.32	6.75	2.86	0.56	6.96	0.66	0.14	35.38	0.20
2.09+402	2.01	0.96	4.06	6.45	1.55	25.61	14.61	0.78	1.35	2.47	0.13	0.38	9.21	0.32
2.22+402	0.70	0.07	1.73	0.49	1.85	11.36	6.89	2.75	0.43	6.71	0.69	0.06	34.73	0.03
2.28+402	1.23	0.94	10.05	6.56	2.58	11.60	0.57	0.03	0.76	0.17	0.60	1.20	0.24	0.09
2.37+402	2.93	1.80	3.22	13.71	1.70	18.87	10.83	0.33	1.46	1.70	0.46	0.45	5.76	0.19
2.41+296	0.14	0.06	46.98	0.22	11.15	3.85	0.66	0.13	0.20	0.13	7.02	11.26	0.03	0.75
2.41+402	0.55	0.64	16.91	2.41	5.31	13.13	4.87	0.20	0.79	0.19	0.30	3.54	0.22	0.07
2.46+402	0.13	0.21	24.50	1.52	9.66	2.62	0.95	0.01	0.64	0.16	4.75	2.63	0.01	0.34
2.61+402	9.44	2.02	5.47	8.28	1.83	4.44	7.53	1.02	4.75	1.64	0.52	0.07	6.10	0.08
3.10+402	9.05	2.44	5.88	16.37	1.99	3.47	5.06	0.75	7.01	0.96	0.37	0.30	4.80	0.08
3.29+402	1.26	0.14	1.22	1.14	2.51	10.58	6.84	2.81	1.22	7.17	1.09	0.04	46.01	0.32
5.01+402	0.39	0.01	4.05	0.19	8.93	10.00	2.65	2.14	0.52	6.23	3.46	0.09	32.92	1.61
5.24+402	1.01	0.18	6.08	0.18	10.93	10.75	5.77	2.86	0.44	7.52	2.63	0.08	20.38	2.36
7.02o+402	2.07	1.55	5.28	7.71	6.91	12.81	6.03	0.34	1.99	1.02	2.64	0.21	3.68	5.02
7.10+402	1.04	0.73	9.46	2.82	4.75	15.56	8.14	0.39	0.78	2.25	2.05	0.59	4.62	0.23
7.27o+402	1.76	1.69	3.42	7.67	2.15	20.81	9.07	0.48	0.90	1.80	0.60	0.39	5.72	0.82
8.15+402	1.43	0.40	5.09	5.24	6.63	14.56	0.65	0.13	3.18	0.55	11.54	0.31	3.51	1.17
AaFS-402	4.40	3.02	1.78	19.11	1.25	24.08	13.33	0.50	2.45	1.45	0.67	0.06	4.59	0.02
WT+402	4.67	2.01	1.67	19.95	0.90	23.94	14.02	0.36	3.51	1.15	0.07	0.10	4.76	0.07
7.09+402	0.62	0.14	10.06	1.08	17.37	4.24	3.42	0.37	0.34	0.76	8.09	0.14	1.35	1.93
1.25-402	0.16	0.02	1.22	0.08	1.17	1.87	0.54	0.19	0.42	0.42	0.27	0.04	0.01	0.08
2.28-402	0.04	0.02	1.02	0.02	1.90	0.48	0.32	0.02	0.21	0.07	0.88	0.02		0.05
2.37-402	0.11		0.42	0.08	0.83	1.26	0.47	0.13	0.18	0.29	0.17	0.02		0.05
3.10-402	0.13	0.05	1.69	0.06	3.05	1.42	0.75	0.20	0.32	0.37	2.88	0.08	0.07	1.06
7.27-402	0.06		0.83	0.02	2.04	1.08	1.17	0.09	0.09	0.31	0.53	0.01		0.11
AaFS-467	0.57	0.06	0.82	0.26	2.19	4.16	1.05	0.59	0.46	1.66	1.06	0.03	0.01	0.08
2.09-2-402	0.08		0.69	0.06	0.95	0.86	0.13	0.04	0.08	0.11	1.39	0.05	0.01	0.09
2.39-402	0.08	0.01	0.45	0.10	0.95	0.55	0.22	0.01	0.33	0.03	0.20	0.01		0.06
3.03-402	0.06	0.01	0.59	0.08	1.22	0.44	0.17	0.04	0.00	0.04	0.37	0.04		0.05
3.26-402	0.15	0.10	1.60	0.09	2.14	0.96	0.67	0.06	0.41	0.19	0.49	0.10	0.08	1.02
4.12-402	0.11	0.01	0.58	0.08	1.27	0.70	0.35	0.02	0.76	0.09	0.38	0.02		0.08
7.11-402	0.07		1.98	0.08	3.81	1.10	0.67	0.04	0.24	0.11	2.08	0.03		0.15
7.30o-402	0.11	0.03	2.61	0.09	3.98	1.44	0.24	0.10	0.66	0.35	1.03	0.06	0.15	1.35
AaFS-430	0.05	0.03	0.33	0.09	0.61	0.71	0.35	0.02	0.06	0.05	0.74	0.01		0.02
1.16+467	0.18		8.22	0.09	13.99	2.54	0.44	0.16	0.50	0.38	17.10	0.45	0.26	3.80
1.25+467	0.16	0.10	5.08	0.28	2.37	1.95	0.67	0.31	0.68	0.52	1.06	0.11	1.07	0.54
1.34+467	0.13		4.49	0.07	7.16	5.06	0.56	0.76	0.39	1.94	6.09	0.07	8.28	0.64
1.37+467	0.12	0.04	2.92	0.64	2.04	3.30	0.38	0.07	0.34	0.19	0.20	0.28	0.33	0.14
2.09+467	0.12	0.02	0.94	0.26	0.53	1.95	0.35	0.16	0.50	0.24	0.08	0.02	0.30	0.07
2.22+467	0.07		4.22	0.07	6.66	4.65	1.11	0.79	0.14	1.57	5.04	0.07	6.57	1.05
2.28+467	0.03		3.15	0.08	1.32	1.10	0.18	0.01	0.13	0.04	0.54	0.30		0.02
2.37+467	0.10	0.08	1.66	0.21	1.77	1.68	0.89	0.11	0.25	0.31	1.47	0.09	0.11	0.44
2.46+467		0.01	7.43	0.15	2.77	0.77	0.39	0.01	0.15	0.05	1.49	0.65		0.06
2.61+467	0.36		6.03	0.39	5.49	3.17	2.02	0.26	1.49	0.41	5.80	0.04	0.59	0.13
3.10+467	0.21	0.08	5.06	0.24	6.71	2.61	1.19	0.27	0.62	0.95	7.88	0.34	0.37	3.51
3.36+467	0.14	0.08	5.91	0.89	22.56	1.71	0.33	0.03	0.91	0.07	1.90	0.71	0.01	0.78
4.02+467	0.04		6.27	0.04	11.95	0.28	0.26	0.02		0.31	9.58	0.03		0.69
5.24+467	0.04		11.58		21.19	1.73	8.06	0.06		0.44	11.85	0.26	0.08	0.90
7.02o+467	0.05		7.36	0.07	15.20	1.12	2.02	0.02		0.10	10.33	0.08		1.06
7.10+467	0.07	0.01	0.60	0.11	1.00	0.63	0.28	0.01	0.14	0.06	0.45	0.01	0.01	0.03
7.27+467	0.02		2.93	0.02	4.19	1.41	1.10	0.03	0.18	0.19	2.28	0.03	0.02	0.37
7.27o+467	0.02		3.54	0.24	7.54	1.86	1.29	0.03	0.52	0.11	4.61	0.07	0.17	0.38
8.15+467	0.07		7.74	0.13	15.42	1.47		0.06	0.56	0.25	19.06	0.43	0.07	4.79
AaFS-402/467	0.57	0.09	2.10	1.31	1.11	6.03	0.52	0.29	1.43	0.53	1.26	0.02	0.47	0.03
WT+467	0.61	0.08	2.71	1.24	2.18	6.11	2.21	0.34	1.60	0.50	0.49	0.05	0.39	0.79
1.03+467	0.06		10.05	0.03	20.17	0.78	2.26	0.03	0.20	0.29	8.68	0.22		4.62
7.09+467	0.08	0.02	11.94	0.06	21.13	1.54	3.95	0.11	0.41	0.30	6.96	0.40		4.08
2.05+402	0.52	0.01	10.70	0.16	6.49	14.59	7.34	0.36	0.42	2.00	2.78	1.44	2.49	0.07
2.12+402	1.92	0.73	9.80	5.55	4.55	32.17	9.53	0.23	1.80	1.23	3.34	1.34	0.74	0.43
2.39+402	4.82	2.89	3.72	17.52	2.28	21.10	11.20	1.12	3.27	3.83	0.27	0.27	14.37	0.12
3.03+402	1.41	0.69	34.57	3.24	9.29	13.19	6.84	0.70	1.54	1.24	0.58	6.89	0.50	0.08
3.05+402	0.65	0.86	24.98	2.17	50.46	2.17	1.09	0.18	0.97	0.25	0.89	3.53	0.03	0.18
3.26+402	1.65	0.69	12.62	3.85	1.24	28.03	9.37	0.32	0.43	6.17		1.10	12.66	
3.36+402	2.47	1.85	16.12	9.56	25.73	2.82	1.70	0.08	2.00	0.19	4.37	2.21	0.06	4.95
4.12+402	4.60	2.29	4.93	9.87	5.00	24.36	8.86	1.04	3.26	3.97	1.41	0.30	7.94	1.22
7.11+402	2.97	1.64	7.14	7.13	8.59	21.34	3.49	0.59	3.81	2.81	6.17	0.19	5.76	0.43
7.30o+402	2.38	0.81	6.96	3.23	3.14	17.17	10.27	1.80	1.58	7.71	0.10	0.61	19.83	0.30
9.31+402	0.66	0.04	2.28	0.77	3.01	38.95	17.92	1.54	0.74	5.49	1.77	0.04	14.87	0.12
AaFS-402/430	5.15	2.97	3.18	17.13	1.91	26.61	12.26	0.80	3.36	3.64	0.70	0.14	10.26	0.02
1.37-402	0.06	0.01	1.88	0.09	3.00	1.41	0.48	0.06	0.38	0.22	0.75	0.03	0.04	1.07
AaFS-467/430	0.16	0.01	0.67	0.12	1.53	1.73	0.56	0.16	0.16	0.50	0.86	0.02	0.01	0.04
1.37+430	1.11	0.51	16.50	1.75	4.73	9.08	0.98	0.48	0.88	2.25	0.81	2.07	0.31	0.03
1.37+430	0.65	0.24	13.36	1.65	3.79	7.47	4.19	0.25	1.06	1.35	0.22	2.48	0.44	0.10
2.28+430	0.35	0.12	18.77	0.51	3.94	5.74	0.80	0.13	0.19	1.41	0.51	2.99	0.13	0.04
2.37+430	0.48	0.11	5.94	0.60	3.55	9.14	0.88	0.42	0.61	5.47	1.86	0.34	4.33	0.20
2.39+430	1.17	0.15	5.59	2.89	7.92	9.55	3.34	0.26	4.09	2.65	4.95	0.12	4.53	0.40
2.46+430	0.06	0.07	28.25	0.19	6.93	1.77	0.28	0.02	0.18	0.76	0.58	5.05	0.02	0.04
3.05+430	0.48	0.42	43.91	1.22	23.93	2.33	0.96	0.19	1.27	0.23	1.13	7.05	0.03	0.04
3.10+430	1.20	0.27	6.24	2.42	3.28	6.39	1.17	0.80	0.98	10.47	2.99	0.42	2.88	0.04
3.26+467	0.50	0.10	6.49	0.71	5.49	6.53	1.09	0.54	1.31	2.80	1.77	0.81	2.81	2.05
3.36+430	0.26	0.11	33.04	2.28	34.70	2.44	0.42	0.01	1.15	0.05	0.41	4.67	0.01	0.00
7.27+430					6.11		0.00	0.00	0.00		5.16			1.53
7.30o+467	0.51	0.08	5.95	0.60	5.60	5.87	3.57	0.81	1.15	2.80	1.72	0.50	4.45	1.74
AaFS-402/467/430	2.93	0.83	2.59	6.10	2.07	17.87	8.75	0.56	4.52	3.59	1.57	0.07	5.32	0.05

Supplementary Table 7. Quantitative analysis of sesquiterpene products synthesized by the *C. junos* BFS M3 library, native enzymes and non-natural Y402 substitutions

Mutant name	Number of mutations	Kinetic parameters: Protein concentration (μM)	kcat apparent (min^{-1})	% of AaFS-WT activity	Product analysis: Linear products (%)	Cyclic products (%)	Background: 402	467	430
**CjFS M3 library**									
WT	0		0.587	15.70	85	15	Y	G	L
Y402L	1		0.449	12.02	81	19	L	G	L
G467V	1		0.339	12.77	44	56	Y	V	L
L430A	1		0.087	2.32	81	19	Y	G	A
Y402L/G467V	2		0.215	5.75	61	39	L	V	L
Y402L/L430A	2		0.228	6.10	76	24	L	G	A
G467V/L430A	2		0.195	5.22	75	25	Y	V	A
Y402L/G467V/L430A	3		0.183	4.88	77	23	L	V	A
**Native enzymes**									
AaADS	0	2.46	1.25	33.48	2	98			
AaGAS	0	3.17	6.86	183.18	2	98			
CjFS	0	1.15	0.45	12.10	86	14			
LsGAS2	0	2.34	8.53	227.74	2	98			
MpFS	0	2.69	0.86	22.94	98	2			
TEAS	0	6.34	4.55	121.55	1	99			
**Non-natural Y402 substitutions**									
Y402F	1				85	15			
Y402W	1				93	7			
Y402L	1				26	74			
Y402T	1				20	80			
Y402I	1				21	79			
Y402V	1				20	80			

Mutant name	sesquithujene	α-cedrene	Unknown 1	α-exo-bergamotene	Peak 1	(E)-β-farnesene	amorphadiene	γ-curcumene	ar-curcumene	cis-α-bergamotene	zingiberene
	7.77 min	8.26 min		8.52 min	8.62 min	8.75 min	9.00 min	9.24 min	9.29 min	9.39 min	9.49 min
WT					1.16	81.58	2.60	2.89	0.57	1.45	1.05
Y402L					1.56	58.36	2.99	1.75	0.93	8.42	0.65
G467V	0.51	1.74	2.80	3.51	1.36	40.67	7.28	13.03		1.19	3.06
L430A				0.51		76.77	3.81	3.73	0.59	1.59	2.25
Y402L/G467V				2.24		43.12	2.67	5.04	1.54	6.89	0.95
Y402L/L430A					0.95	57.65	3.19	1.97	0.86	8.23	1.24
G467V/L430A	0.38			0.44	1.33	72.05	3.07	6.34	0.90	1.15	1.24
Y402L/G467V/L430A					0.80	60.41	2.16	2.71	0.83	7.60	

Mutant name	α-farnesene	β-bisabolene	β-curcumene	Peak 2	β-sesquiphellandrene	α-bisabolene	nerolidol	Peak 4	α-bisabolol	farnesol
	9.64 min	9.73 min	9.8 min	9.89 min	10.02 min	10.32 min	10.66 min	11.27 min	13.22 min	13.59 min
WT	3.84	0.99	2.24	1.62						
Y402L	22.42			0.58		0.90		1.44		
G467V	3.47		15.80	1.45	3.38	0.76				
L430A	4.46		2.68		3.63					
Y402L/G467V	17.48	4.43	11.61	2.86		1.17				
Y402L/L430A	18.40	4.19		0.37	1.45	0.67		0.82		
G467V/L430A	2.77		7.65	0.00	2.03					
Y402L/G467V/L430A	16.84	2.98	4.26	0.74		0.66				

Supplementary Table 8. Dominance of the Y402 position in different BFS mutant backgrounds
A subset of the BFS 6Å library was extracted to examine the effect of the 402 mutation on enzyme activity. For each mutant background, the left and right hand column groups shows kcat apparent in the absence or presence of the 402 mutations respectively, as well as the percentage cyclic products that are made. The original mutant identified from screening is shaded in light grey.

Mutant background	-402 (Y402)			+402 (Y402L)			% change in cyclic products
	k_{cat} apparent		Cyclic products	k_{cat} apparent		Cyclic products	
	min^{-1}	% BFS-WT activity	%	min^{-1}	% BFS-WT activity	%	
**1.03**	0.60	2.02	7	0.98	3.29	31	24
**1.16**	1.19	3.48	2	1.72	5.02	40	38
**3.03**	1.98	6.38	2	1.03	3.30	72	70
**4.12**	3.03	9.77	3	0.84	2.71	74	71
**7.11**	1.02	3.52	4	0.59	2.03	58	54
**8.15**	0.84	2.46	2	0.56	1.64	35	33
**WT**	37.45	100	1	24.10	77.14	78	77
**1.03**	0.60	2.02	7	0.98	3.29	31	24
**1.34**	0.88	2.47	6	1.27	4.08	71	65
**1.37**	1.56	5.00	5	1.07	3.69	37	32
**2.09**	1.74	4.00	2	1.87	5.99	69	67
**2.39**	7.61	21.49	2	8.32	23.49	87	85
**2.41**	3.04	8.57	3	6.22	17.55	44	41
**2.61**	2.74	7.72	8	8.69	24.53	75	67
**3.26**	1.07	3.43	5	1.70	3.92	77	72
**7.02o**	3.28	9.25	2	1.52	4.28	44	42
**7.10**	1.84	5.94	2	2.88	9.29	47	45
**7.27o**	13.21	37.30	2	3.43	9.67	54	52
**7.30o**	0.90	2.88	6	1.12	3.59	74	68

Supplementary Table 9. Dominance of the second-site suppression position (V467) in different BFS Y402L mutant backgrounds

A subset of the BFS 6Å library in the Y402L background was extracted to examine the effect of the 467 mutation on enzyme activity. For each mutant background, the left and right hand column groups shows kcat apparent in the absence or presence of the 467 mutations respectively, as well as the percentage cyclic products that are made. The original mutant identified from screening is shaded in light grey.

Mutant background	-467 (Y402L)			+467 (Y402L/V467G)			% change in cyclic products
	k_{cat} apparent		Cyclic products	k_{cat} apparent		Cyclic products	
	min^{-1}	% BFS-WT activity	%	min^{-1}	% BFS-WT activity	%	
**2.61**	8.69	24.53	75	0.82	2.65	16	58
**2.09**	1.87	5.99	69	3.41	7.86	5	64
**WT**	32.97	77.69	77	0.38	0.90	14	63
**7.02o**	1.52	4.28	44	0.73	2.36	11	33
**7.27o**	3.43	9.67	54	1.18	3.81	8	46
**7.10**	2.88	9.29	47	2.05	6.62	2	44
**1.03**	0.98	3.29	31	0.30	1.01	14	17
**2.22**	2.94	9.86	68	0.78	2.62	19	49
**1.34**	1.27	4.08	71	0.29	0.97	22	49
**5.24**	0.44	1.50	56	0.44	1.52	22	34
**2.28**	0.57	1.85	34	0.91	2.98	5	29
**1.25**	10.93	25.18	66	1.03	2.37	11	55
**2.46**	0.64	2.09	33	0.54	1.77	10	24
**3.10**	4.44	10.23	77	0.32	0.75	13	65
**2.37**	1.67	3.84	62	1.50	3.46	6	56

Supplementary Table 10. Dominance of the reactivation position (Y430) in different BFS Y402L/V467G mutant backgrounds
A subset of the BFS 6Å library in the Y402L/V467G background was extracted to examine the effect of the 430 mutation on enzyme activity. For each mutant background, the left and right hand column groups shows kcat apparent in the absence or presence of the 430 mutation respectively, as well as the percentage cyclic products that are made. The original mutant identified from screening is shaded in light grey.

Mutant background	-430 (Y402L/V467G)			+430 (Y402L/V467G/Y430A)			% change in cyclic products
	k_{cat} apparent		Cyclic products	k_{cat} apparent		Cyclic products	
	min^{-1}	% BFS-WT activity	%	min^{-1}	% BFS-WT activity	%	
**2.28**	0.91	2.98	5	1.30	4.26	31	26
**7.27**	0.49	1.60	6	0.54	1.77	25	19
**2.46**	0.54	1.77	10	0.80	2.62	37	27
**2.37**	1.50	3.46	6	1.50	4.91	28	23
**3.10**	0.32	0.75	13	1.86	6.00	39	26
**WT**	0.38	0.90	14	0.00	1.08	55	41
**3.36**	1.66	5.34	11	3.19	10.72	45	34
**1.37**	1.23	3.92	9	1.07	3.69	37	28

Supplementary Table 11. Kinetic measurements for *A. annua* BFS and *C. junos* BFS M3 mutant libraries

k_{cat} apparent measurements for mutants from the *A. annua* BFS and *C. junos* BFS M3 library. Values for each mutant are expressed as a percentage of the *C. junos* BFS-WT and/or the *A. annua* BFS-WT activity to allow for comparison.

A. annua (E)-β-farnesene synthase	k_{cat} apparent (min^{-1})	% *A. annua* BFS-WT activity	% cyclic products	Protein purification
WT	4.435	100	2.0	a
Y402L	3.297	74.34	73.6	a
V467G	0.215	4.85	12.4	a
Y430A	1.120	25.26	2.5	a
Y402L/V467G	0.027	0.61	15.8	a
Y402L/Y430A	1.914	43.16	86.8	a
V467G/Y430A	0.189	4.25	5.8	a
Y402L/V467G/Y430A	0.162	6.20[1]	51.6	b

C. junos (E)-β-farnesene synthase[3]	k_{cat} apparent (min^{-1})	% *C. junos* BFS-WT activity	% *A. annua* BFS-WT activity	% cyclic products	Protein purification
WT	0.587	100	15.70	14.6	c
Y402L	0.449	76.56	12.02	19.2	c
G467V	0.339	81.33[2]	12.77[2]	55.9	d
L430A	0.087	14.79	2.32	18.8	c
Y402L/G467V	0.215	36.66	5.75	39.4	c
Y402L/L430A	0.228	38.87	6.10	24.0	c
G467V/L430A	0.195	33.25	5.22	25.2	c
Y402L/G467V/L430A	0.183	31.12	4.88	22.8	c

[1] % activity quantified again *A. annua* BFS-WT from protein purification b (2.615 min^{-1})

[2] % activity quantified again *A. annua* BFS-WT from protein purification d (2.655 min^{-1})

[3] Residues are numbered according to *A. annua* BFS. The corresponding residue numbers in *C.junos* EBFS are 387 (402), 453 (467), 415 (430)

Supplementary Table 12. Measures of ruggedness and epistasis for *A. annua* and *C. junos* landscape

The source code for the analysis and visualisation was implemented in the Python programming language[5,6,7]. There is higher amount of epistasis present in the *C. junos* landscape, F_{sum} = 0.423 in comparison to *A. annua* with F_{sum} = 0. 263 which suggests that the *C. junos* landscape is highly rugged. The total amount of pairwise interactions, F_2 is also higher in *C. junos*, though it has a relatively lower net main effect F_1. The Fourier analysis and the linear model fitting presume that the mutations interact additively in the absence of epistasis. We have also implemented a deviation measure if the mutational effect is multiplicative as defined in Østman et al [8]. The dominant pairwise epistasis was, $\epsilon_{402,467}$ = -1.461 in *A. annua* and $\epsilon_{467,430}$ = -0.458 for *C. junos*. A notable triple epistasis for *A. annua* was $\epsilon_{402,467,430}$ = -1.043 and for *C. junos* was $\epsilon_{402,467,430}$ = -0.618.

	Mutation	*A. annua* BFS	*C. junos* BFS
Relative fitness	WT	1.000	1.000
	402	36.800	1.315
	467	6.200	3.829
	430	1.250	1.288
	402/467	7.900	2.699
	402/430	43.400	1.644
	467/430	2.900	1.726
	402/467/430	25.800	1.562
Coefficients (basis: 0,1)	WT	1.000	1.000
	402	35.800	0.315
	467	5.200	2.829
	430	0.250	0.288
	402/467	-34.100	-1.445
	402/430	6.350	0.041
	467/430	-3.550	-2.390
	402/467/430	14.850	0.925
Coefficients (basis: -1,+1)	WT	15.656	1.883
	402	12.819	-0.078
	467	-4.956	0.571
	430	2.681	-0.328
	402/467	-6.669	-0.246
	402/430	3.444	0.126
	467/430	0.969	-0.482
	402/467/430	1.856	0.116
F1		0.764	0.577
F2		0.223	0.405
F3		0.013	0.018
Fsum (=F2+F3)		0.236	0.423

Supplementary Table 13. Retention time and ion pairs used for Met-Idea analysis

ion (m/z)	RT (min)	MZ tolerance	RT tolerance	Product
119	7.765	0.2	0.017	sesquithujene
93	7.765	0.2	0.017	sesquithujene
69	7.765	0.2	0.017	sesquithujene
77	7.765	0.2	0.017	sesquithujene
105	7.765	0.2	0.017	sesquithujene
85	7.765	0.2	0.017	sesquithujene
119	8.519	0.2	0.017	α-exo-bergamotene
93	8.519	0.2	0.017	α-exo-bergamotene
107	8.519	0.2	0.017	α-exo-bergamotene
69	8.519	0.2	0.017	α-exo-bergamotene
79	8.519	0.2	0.017	α-exo-bergamotene
161	8.519	0.2	0.017	α-exo-bergamotene
69	8.629	0.2	0.017	Peak 1
93	8.629	0.2	0.017	Peak 1
161	8.629	0.2	0.017	Peak 1
77	8.629	0.2	0.017	Peak 1
133	8.629	0.2	0.017	Peak 1
120	8.629	0.2	0.017	Peak 1
69	8.754	0.2	0.017	(E)-β-farnesene
93	8.754	0.2	0.017	(E)-β-farnesene
133	8.754	0.2	0.017	(E)-β-farnesene
79	8.754	0.2	0.017	(E)-β-farnesene
120	8.754	0.2	0.017	(E)-β-farnesene
161	8.754	0.2	0.017	(E)-β-farnesene
119	8.997	0.2	0.017	putative amorphadiene isomer
93	8.997	0.2	0.017	putative amorphadiene isomer
189	8.997	0.2	0.017	putative amorphadiene isomer
79	8.997	0.2	0.017	putative amorphadiene isomer
105	8.997	0.2	0.017	putative amorphadiene isomer
204	8.997	0.2	0.017	putative amorphadiene isomer
119	9.241	0.2	0.017	γ-curcumene
93	9.241	0.2	0.017	γ-curcumene
204	9.241	0.2	0.017	γ-curcumene
105	9.241	0.2	0.017	γ-curcumene
79	9.241	0.2	0.017	γ-curcumene
134	9.241	0.2	0.017	γ-curcumene
132	9.286	0.2	0.017	ar-curcumene
202	9.286	0.2	0.017	ar-curcumene
145	9.286	0.2	0.017	ar-curcumene
93	9.389	0.2	0.017	cis-α-bergamotene
119	9.389	0.2	0.017	cis-α-bergamotene
107	9.389	0.2	0.017	cis-α-bergamotene
69	9.389	0.2	0.017	cis-α-bergamotene
79	9.389	0.2	0.017	cis-α-bergamotene
161	9.389	0.2	0.017	cis-α-bergamotene
119	9.493	0.2	0.017	zingiberene
93	9.493	0.2	0.017	zingiberene
77	9.493	0.2	0.017	zingiberene
69	9.493	0.2	0.017	zingiberene
105	9.493	0.2	0.017	zingiberene
204	9.493	0.2	0.017	zingiberene

Supplementary Table 13 continued

ion (m/z)	RT (min)	MZ tolerance	RT tolerance	Product
93	9.642	0.2	0.017	α-farnesene
107	9.642	0.2	0.017	α-farnesene
69	9.642	0.2	0.017	α-farnesene
119	9.642	0.2	0.017	α-farnesene
79	9.642	0.2	0.017	α-farnesene
123	9.642	0.2	0.017	α-farnesene
79	9.731	0.2	0.017	α-farnesene
109	9.731	0.2	0.017	α-farnesene
105	9.795	0.2	0.017	α-farnesene
93	9.891	0.2	0.017	Peak 2
119	9.891	0.2	0.017	Peak 2
107	9.891	0.2	0.017	Peak 2
204	9.891	0.2	0.017	Peak 2
134	9.891	0.2	0.017	Peak 2
69	10.019	0.2	0.017	β-sesquiphellandrene
133	10.019	0.2	0.017	β-sesquiphellandrene
161	10.019	0.2	0.017	β-sesquiphellandrene
109	10.019	0.2	0.017	β-sesquiphellandrene
204	10.019	0.2	0.017	β-sesquiphellandrene
93	10.317	0.2	0.017	α-bisabolene
119	10.317	0.2	0.017	α-bisabolene
80	10.317	0.2	0.017	α-bisabolene
109	10.317	0.2	0.017	α-bisabolene
204	10.317	0.2	0.017	α-bisabolene
67	10.317	0.2	0.017	α-bisabolene
69	10.658	0.2	0.017	nerolidol
93	10.658	0.2	0.017	nerolidol
107	10.658	0.2	0.017	nerolidol
81	10.658	0.2	0.017	nerolidol
136	10.658	0.2	0.017	nerolidol
161	10.658	0.2	0.017	nerolidol
135	11.272	0.2	0.017	Peak 4
107	11.272	0.2	0.017	Peak 4
93	11.272	0.2	0.017	Peak 4
204	11.272	0.2	0.017	Peak 4
77	11.272	0.2	0.017	Peak 4
119	11.272	0.2	0.017	Peak 4
109	13.224	0.2	0.017	α-bisabolol
119	13.224	0.2	0.017	α-bisabolol
69	13.224	0.2	0.017	α-bisabolol
93	13.224	0.2	0.017	α-bisabolol
204	13.224	0.2	0.017	α-bisabolol
161	13.224	0.2	0.017	α-bisabolol
69	13.591	0.2	0.017	farnesol
93	13.591	0.2	0.017	farnesol
81	13.591	0.2	0.017	farnesol
107	13.591	0.2	0.017	farnesol
136	13.591	0.2	0.017	farnesol
121	13.591	0.2	0.017	farnesol

Supplementary references

1. Liu, H. & Naismith, J. H. An efficient one-step site-directed deletion, insertion, single and multiple-site plasmid mutagenesis protocol. *BMC Biotechnol* **8**, 91, (2008).
2. Juteau, F., Masotti, V., Bessi, J. M., Dherbomez, M. & Viano, J. Antibacterial and antioxidant activities of Artemisia annua essential oil. *Fitoterapia* **73**, 532-535, (2002).
3. Goel, D., Goel, R., Singh, V., Ali, M., Mallavarapu G. R., Kumar, S. Composition of the essential oil from the root of Artemisia annua. *J. Nat. Med.* **61**, 458-461, (2007)
4. Lopes-Lutz, D., Alviano, D. S., Alviano, C. S. & Kolodziejczyk, P. P. Screening of chemical composition, antimicrobial and antioxidant activities of Artemisia essential oils. *Phytochemistry* **69**, 1732-1738, (2008).
5. Python Language Reference v. 2.7 (Available at http://www.python.org).
6. Pedregosa, F. *et al.* Scikit-learn: Machine Learning in Python. *J. Mach. Learn. Res.* **12**, 2825-2830, (2011).
7. Hagberg, A. A., Schult, D. A. & Swart, P. J. in *Proceedings of the 7th Python in Science Conference (SciPy2008).* (eds G. Varoquaux, T Vaught, & J Millman) 11-15.
8. Ostman, B., Hintze, A. & Adami, C. Impact of epistasis and pleiotropy on evolutionary adaptation. *Proceedings. Biological sciences / The Royal Society* **279**, 247-256, (2012).
